# Prism[5]arene Iminosugar Bioconjugates Are Potent Multivalent Inhibitors and Mutation Specific Pharmacological Chaperones of Glucocerebrosidase (GCase) Activity

**DOI:** 10.1021/acs.bioconjchem.6c00273

**Published:** 2026-08-03

**Authors:** Anna Palmieri, Francesca Clemente, Maria Manuela Ignoto, Fabio Buonsenso, Miriana Attanasio, Margherita De Rosa, Carmen Talotta, Paolo Della Sala, Rocco del Regno, Veronica Iuliano, Amelia Morrone, Francesca Cardona, Andrea Goti, Camilla Matassini, Carmine Gaeta

**Affiliations:** † Laboratory of Supramolecular Chemistry, Dipartimento di Chimica e Biologia “A. Zambelli”, Università di Salerno, Via Giovanni Paolo II 132, 84084 Fisciano, Salerno, Italy; ‡ Dipartimento di Chimica “Ugo Schiff”, Università degli Studi di Firenze, via della Lastruccia 3-13, 50019 Sesto Fiorentino, FI, Italy; § Laboratory of Molecular Biology of Neurometabolic Diseases, Neuroscience Department, Meyer Children’s Hospital, IRCCS, Viale Pieraccini 24, 50139 Firenze, Italy; ∥ Department of Neurosciences, Psychology, Drug Research and Child Health, 9300 University of Florence, Viale Pieraccini 24, 50139 Firenze, Italy

## Abstract

When trihydroxypiperidine (PIP) or DAB-1 pyrrolidine (PYR) iminosugars are multimerized on the prism[5]­arene scaffold, they generate highly efficient multivalent inhibitors of GCase. Binding trihydroxypiperidine to the rims of prism[5]­arene (**PrS­[5]** ^***PIP***^) results in a multivalent derivative that is more effective than others reported in the literature with the same bioactive unit. This efficacy is attributed to the role of prism[5]­arene, whose barrel-like shape exposes an external π-surface that enables van der Waals contacts and other secondary interactions with the enzyme, thereby enhancing affinity for GCase through dimer stabilization. The high efficiency of **PrS­[5]** ^***PIP***^ is supported by *in silico* calculations that showed how the crowding of the bioactive units on the rims of the macrocycle promotes a rearrangement allowing the active site to accommodate two trihydroxypiperidine units, which engage in hydrogen bonding with the catalytically active Glu residues. Due to their inhibitory potency, these prismarene derivatives have been tested as pharmacological chaperones in intact cell lines from Gaucher patients carrying different mutations. The results indicated that the **PrS­[5]** ^***PIP***^ is more effective toward the N370S mutation, while the DAB-1 pyrrolidine iminosugar **PrS­[5]** ^***PYR***^ unexpectedly enhances L444P mutant GCase activity. This suggests that the type of iminosugar linked to the prismarene scaffold influences GCase activity rescue in a mutation-dependent manner and opens the way for the future development of increasingly efficient pharmacological chaperones.

## Graphical abstract

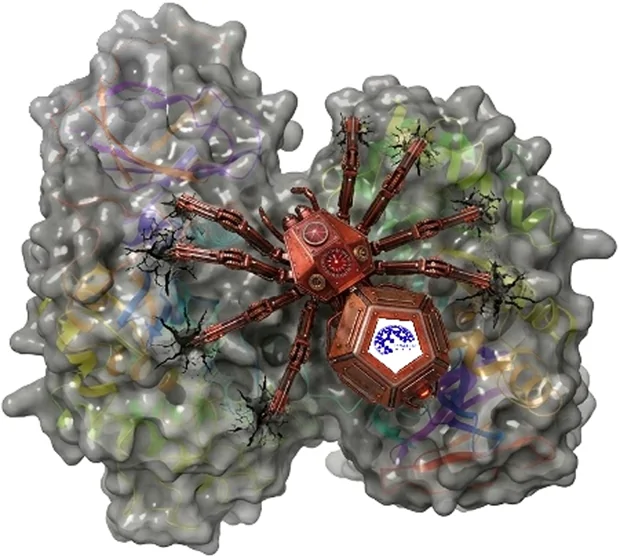

## Introduction

In recent years, considerable effort has been dedicated to the synthesis and investigation of biomimetic macrocyclic scaffolds featuring deep cavities and functionalizable rims.^1–6^ Among these, prism[5]­arene^1,7^ is a planar chiral macrocycle composed of 2,6-dialkoxynaphthalene units bridged by methylene groups. Prism[5]­arene exhibits unique structural, chiral^1,8–10^ and conformational features^1^ which make it a highly promising and specific supramolecular host.^1,7–20^ The prism[5]­arene possesses a deep π-electron-rich aromatic cavity capable of accommodating both cationic^1,7–20^ and neutral guests^12,19^ and features two oxygenated rims amenable to functionalization. In this context, prism[5]­arene derivatives have found applications in various fields of supramolecular chemistry, including molecular^1,7–20^ and chiral recognition,^8,9^ supramolecular catalysis,^17^ and use as supramolecular herbicides.^18^ Additionally, prismarenes exhibit intriguing properties for trapping organic molecules in the solid state, such as in systems for capturing volatile organic compounds (VOCs)^19^ or encapsulating dibromoalkanes within their cavity in the solid state.^20^ To date, no information has been reported on the application of prismarenes in the biological field. However, it has been recently reported that the prism[5]­arene scaffold exhibits excellent biocompatibility.^14^ In the biological field, the multivalent effect^21^ refers to the phenomenon where a compound possessing multiple bioactive units (multivalent ligand) exhibits increased bioactivity compared to the sum of its monovalent counterparts when interacting with specific target receptors. This phenomenon has been thoroughly explored in the study of carbohydrate-lectin interactions^22,23^ and, more recently, in enzyme inhibition, particularly in the interactions of glycomimetics with carbohydrate-active enzymes such as glycosidases.^24–26^ Among these glycomimetics, iminosugars, nitrogen-containing monosaccharide analogues have garnered the most research attention.^27^ Consequently, numerous multivalent iminosugar derivatives have been synthesized by attaching these nitrogenated glycomimetics to diverse macrocyclic scaffolds, including porphyrins,^28^ cyclodextrins,^29^ resorcinarenes,^30^ cyclopeptoids^31^ and gold nanoparticles.^32^ Different glycosidases have been addressed as biological targets, including α-mannosidase,^26^ α-fucosidase,^33,34^ and lysosomal glycosidases involved in lysosomal storage disorders,^35^ such as α-galactosidase,^36^ α-glucosidase,^37^ β-glucocerebrosidase (GCase),^38–43^ and *N*-acetylgalactosamine-6-sulfatase (GALNS).^44–47^

Gaucher disease is the most common lysosomal storage disorder caused by mutations in the *GBA1* gene, which compromises the stability of the native conformation of β-glucocerebrosidase (GCase, Figure 1, top),^48,49^ leading to its accelerated degradation and reduced enzymatic activity. GCase is essential for the breakdown of the lipids glucosylceramide (GlcCer) and glucosylsphingosine (GlcSph). Clinically, the disease is characterized by the pathological accumulation of these substrates within the tissues, resulting in a spectrum of phenotypes that depends on the specific *GBA1* mutation.^48^

**Figure 1:**
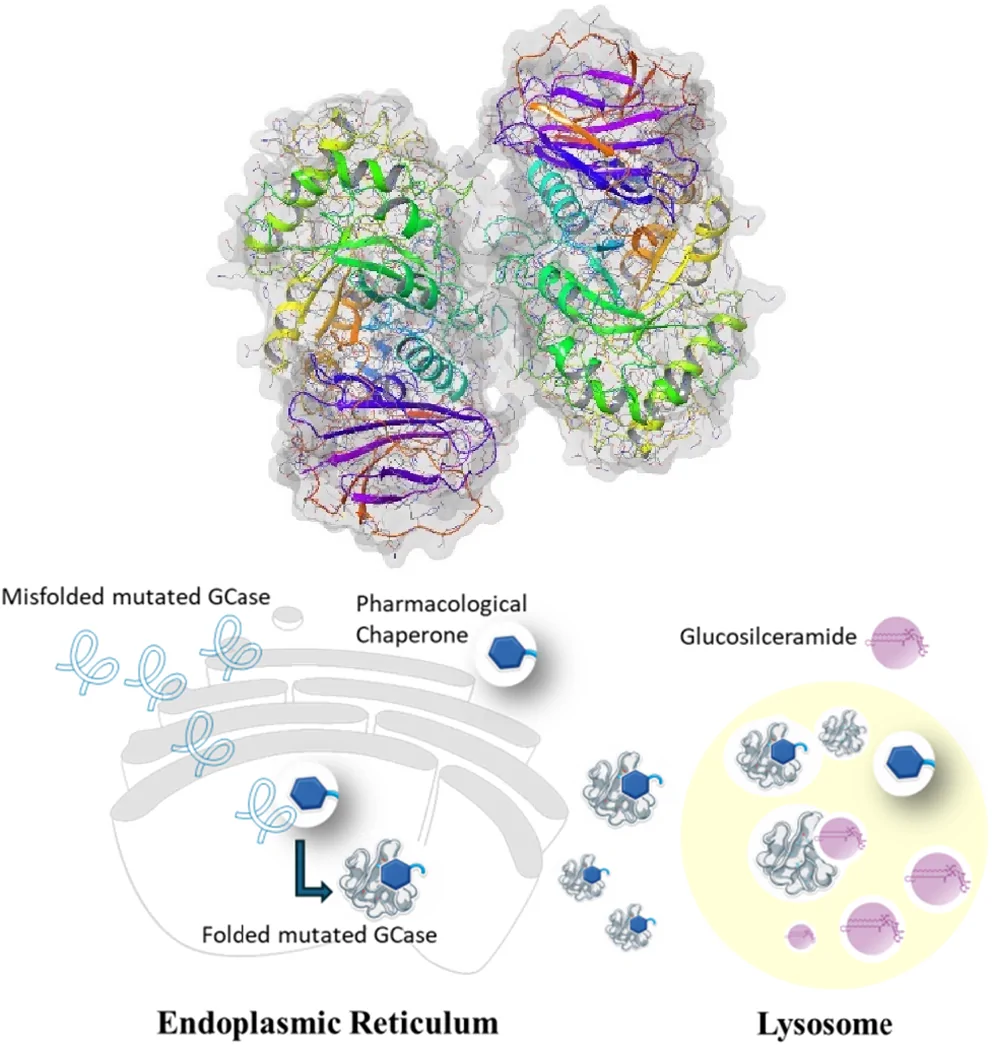
(Top) Crystal structure of the dimeric JZ-5029-modified Glucocerebrosidase (GCase).^49^ (Bottom) The mechanism of a pharmacological chaperone (PC) involves three main stages. In the Endoplasmic Reticulum (ER), the PC binds to a misfolded mutant enzyme to stabilize its correct conformation (left). Once stabilized, the enzyme-chaperone complex is transported to the lysosome (middle). Finally, within the lysosomal environment, the chaperone dissociates from the enzyme, allowing the rescued, functional enzyme to process its natural substrates (right).

N370S and L444P are the most common mutations, typically linked to the non-neuropathic (milder) and neuropathic forms of the disease, respectively. Moreover, *GBA1* mutations are among the most common genetic risk factors for Parkinson’s disease,^50–52^ promoting the accumulation of α-synuclein.^53^ One therapeutic approach for Gaucher disease involves the use of pharmacological chaperones (PCs, Figure 1, bottom).^38,54,55^ This strategy exploits reversible enzyme inhibitors to stabilize and improve the residual hydrolytic activity of deficient enzymes when administered at subinhibitory concentrations (Figure 1). By stabilizing the mutant protein, PCs reduce endoplasmic reticulum–associated degradation and facilitate trafficking to the lysosome, where GCase can efficiently degrade stored substrates (Figure 1). Importantly, even modest increases in enzyme activity can lead to substantial improvements in disease symptoms.

To date, a few multivalent pharmacological chaperones able to rescue mutant GCase activity have been reported, featuring both piperidine^37–40^ and pyrrolidine^41^ iminosugars as bioactive units. In this regard, some of us recently^43^ reported a trivalent dendrimeric trihydroxypiperidine derivative as a potent modulator of GCase. The results underscored the crucial role of the central flat benzene ring on which three bioactive units were multimerized. The central aromatic scaffold is essential for ensuring affinity for the target enzyme through secondary interactions.^43^

Pyrrolo­[2,3-*b*]­pyrazine GCase activators have previously been reported to enhance substrate metabolism within lysosomes in a cellular assay binding a novel pocket of the human GCase dimer interface, inducing its dimerization.^49^ The crystal structure of the activator-GCase complex highlighted the critical role of π···π and van der Waals interactions in stabilizing the complex. The compound specifically binds to the pocket through a π···π stacking interaction involving the central pyrrolo­[2,3-*b*]­pyrazine or 4,7-diazaindole ring, with the side chain of amino acids. This interaction is further supported by additional van der Waals interactions between the activators and the side chains of amino acids in the active site. These examples^43,49^ demonstrate that in the context of GCase modulation, the scaffold that binds the bioactive unit does not merely play a passive structural role; its function is crucial in determining secondary chemical interactions (π···π, C–H···π, and van der Waals) with the enzyme, which are necessary to stabilize the complex with GCase.

Based on these considerations, we envisioned exploiting the structural features of the prism[5] arene macrocycle to enhance the inhibitory activity of piperidine and pyrrolidine iminosugars toward GCase. Prism[5]­arene offers large, barrel-shaped 3D-aromatic surfaces formed by naphthalene rings, which may establish secondary interactions with the protein. Furthermore, due to its synthetic versatility, prism[5]­arene can facilitate the multimerization of 10 bioactive units, providing high biological valency while also strategically positioning the bioactive units on opposite rims. This spatial orientation enables the bioconjugated prism[5]­arene to effectively bridge interactions between distant regions of the GCase protein.

## Results and Discussion

### Bioconjugation of Prism[5]­arenes with 3,4,5-Trihydroxypiperidine (PIP) and DAB-1 Pyrrolidine (PYR) Iminosugars

The multimerization strategy is delineated in Scheme 1 and provided the bioconjugation of prism[5]­arene with 3,4,5-trihydroxypiperidine (PIP) and with DAB-1 pyrrolidine (PYR) iminosugar derivatives via copper­(I)-catalyzed azide–alkyne cycloaddition (CuAAC).

The synthesis of perhydroxylated prism[5]­arene (**PrS­[5]** ^***H***^, Scheme 1) was achieved following the procedure previously reported by some of us.^56^ The synthesis of the new alkyne-terminated prism[5]­arene scaffold, **PrS­[5]** ^***Propargyl***^, was performed by treating **PrS­[5]** ^***H***^ in dry DMF in the presence of propargyl bromide-TIPS (Scheme 1), along with excess sodium carbonate and 18-crown-6. After workup, the crude reaction product was treated with tetrabutylammonium fluoride (TBAF) in THF to afford **PrS­[5]** ^***Propargyl***^ in 27% yield. The alkyne-terminated scaffold **PrS­[5]** ^***Propargyl***^ was subsequently reacted via CuAAC with azide-functionalized **PIPN**_**3**_ ^57^ or **PYRN**_**3**_ ^44^ derivatives (see Scheme 1), whose synthesis had been previously reported by some of us. After that, dialysis was conducted over 3 days using a 1000 Da cutoff membrane to remove salt traces.

**Scheme 1:**
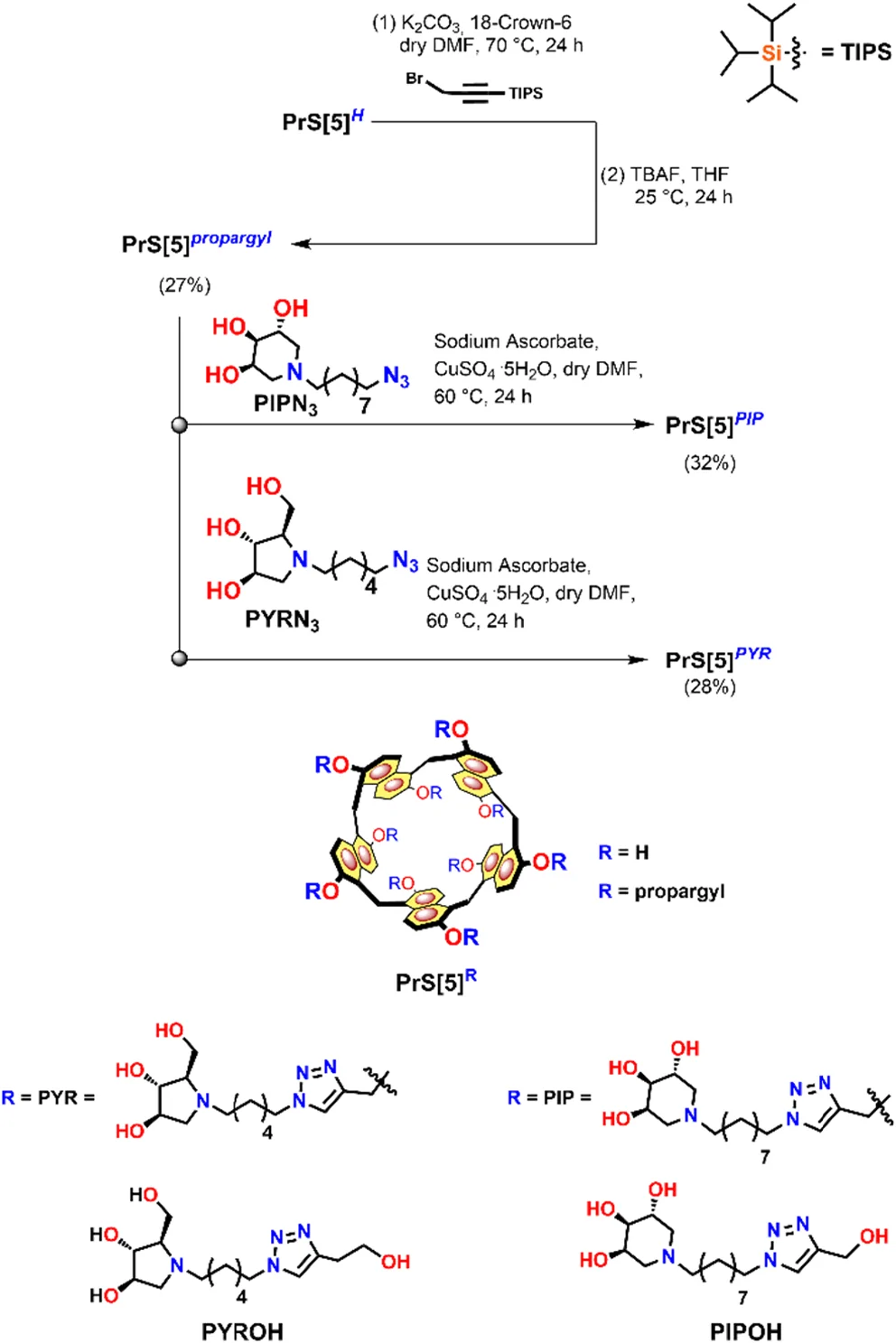
Multimerization of Prism[5]­arene **PrS­[5]** **^*R*^** to Form Bioconjugates **PrS­[5]*^PIP^*** and **PrS­[5]^*PYR*^**

The bioconjugate **PrS­[5]** ^***PIP***^ and **PrS­[5]** ^***PYR***^ products were obtained with yields ranging from 28% (**PrS­[5]** ^***PYR***^) to 32% (**PrS­[5]** ^***PIP***^), which are acceptable considering the multiple conjugation steps involved. Compounds **PrS­[5]** ^***PIP***^ and **PrS­[5]** ^***PYR***^ were characterized by 1D and 2D NMR and HR mass spectra (see Supporting Information). HR-MALDI FT-ICR mass spectrum of **PrS­[5]** ^***PIP***^ showed the presence of a molecular ion peak at 4244.5887 *m*/*z* corresponding to the molecular formula C_225_H_340_N_40_O_40_ (calculated: 4244.5801) and consistent with its structure. HR-MALDI FT-ICR mass spectrum of **PrS­[5]** ^***PYR***^ showed the presence of a molecular ion peak at 3864.2677 *m*/*z* corresponding to the molecular formula C_195_H _280_KN_40_O_40_ and consistent with its structure.

### Biological Studies

Decavalent iminosugars **PrS­[5]** ^***PIP***^ and **PrS­[5]** ^***PYR***^ were first tested as GCase inhibitors to probe their affinity with the target enzyme. When screened toward GCase from leukocytes isolated from healthy donors (controls) at 0.5 mM concentration^58^ **PrS­[5]** **^*PIP*^** and **PrS­[5]** ^***PYR***^ showed 96% and 85% inhibition, respectively (Table 1). **PIPOH** and **PYROH** (Scheme 1) have been chosen as appropriate monovalent reference compound for **PrS­[5]** ^***PIP***^ and **PrS­[5]** ^***PYR***^, respectively. They were synthesized by CuAAC of **PIPN**_**3**_ ^57^ and **PYRN**_**3**_ ^44^ with propargyl alcohol and 3-butyn-1-ol, respectively.

**Table 1:** Inhibitory Activity of Decavalent **PrS­[5]*^PIP^*** and **PrS­[5]^*PYR*^** and Their Corresponding Monovalent Reference Compounds towards GCase Enzyme

entry	compound	% inhibition^a^	IC_50_ (μM)	rp/n^b^
1	**PrS[5]** ^***PIP***^	96	1.4 ± 0.2^c^	36
			2.0 ± 0.1^d^	30
2	**PrS[5]** ^***PYR***^	85	100 ± 20^c^	3.6
			30 ± 3^d^	16
3	**PIPOH**	45^e^	500 ± 20^e^	
			600 ± 60^d^	
4	**PYROH**	14	3600 ± 200^c^	
			4900 ± 500^d^	

a Percentage of GCase (from human leukocytes extracts) inhibition at 0.5 mM inhibitor concentration.

b Rp/n were calculated as follows: the IC_50_ of the monovalent reference compound is divided by the IC_50_ of the multivalent compound (rp) and then divided by ten (valency, n).

c IC_50_ with GCase from human leukocytes extracts.

d IC_50_ with commercially avadlable VPRIV (rhGCase).

e Ref 57.

The IC_50_ values of compounds **PrS­[5]** ^***PIP***^ and **PrS­[5]** ^***PYR***^ against GCase were determined by measuring the initial hydrolysis rate with 4-methylumbelliferyl-β-d-glucopyranoside and fitting the data obtained with an appropriate equation (see Supporting Information). While the decavalent **PrS­[5]** ^***PYR***^ behaved as a modest inhibitor (IC_50_ = 100 μM), **PrS­[5]** ^***PIP***^ showed an IC_50_ value 2 orders of magnitude lower (IC_50_ = 1.4 μM), resulting a more potent inhibitor than the trivalent dendrimeric trihydroxypiperidine analogue previously reported by some of us (IC_50_ = 7 μM).^57^ The lower IC_50_ of **PrS­[5]** ^***PIP***^ compared to **PrS­[5]** ^***PYR***^ can be ascribed to the higher affinity of the *N*-nonyl-trihydroxypiperidine warhead for GCase enzyme with respect to the *N*-hexyl-pyrrolidine one,^58^ as confirmed by the inhibition data of **PIPOH** and **PYROH** (Table 1). To confirm the affinity of **PrS­[5]** ^***PIP***^ and **PrS­[5]** ^***PYR***^ for GCase and exclude off-target interactions, the IC_50_ values of the two compounds were also determined toward the commercially available wild-type recombinant GCase VPRIV (rhGCase VPRIV). While a very close value was obtained for **PrS­[5]** ^***PIP***^ (IC_50_ = 2.0 μM *vs* IC_50_ = 1.4 μM), compound **PrS­[5]** ^***PYR***^ showed higher affinity for the isolated recombinant enzyme (IC_50_ = 30.0 μM *vs* IC_50_ = 100 μM), suggesting that in the more complex leukocytes extract environment the binding to GCase may compete with other lysosomal enzymes.

By comparing the inhibitory activity of **PrS­[5]** ^***PIP***^ and **PrS­[5]** ^***PYR***^ with the corresponding monovalent reference compounds (Entries 3 and 4, Table 1), the occurrence of a multivalent effect emerges for both prismarene bioconjugates. Indeed, positive values of relative potency (rp) per number of bioactive unit (n)^59^ are obtained for the decavalent iminosugars toward both GCase from leukocytes extracts and rhGCase (Table 1). Nevertheless, while modest rp/n values are detected for **PrS­[5]** ^***PYR***^, significantly higher and noteworthy values are observed for **PrS­[5]** ^***PIP***^. To evaluate the intrinsic inhibitory activity of the prismarene scaffold, **PrS­[5]** ^***H***^ and its methyl analogue **PrS­[5]** ^***Me***^ were tested against both leukocyte-derived human wild-type GCase and rhGCase VPRIV (see Supporting Information, Figure S24). **PrS­[5]** ^***H***^ poorly inhibited both GCase sources, producing approximately 50–60% inhibition at 1 mM. The more lipophilic **PrS­[5]** ^***Me***^ showed a similar level of inhibition only against recombinant GCase (VPRIV, 61% at 1 mM), while it was inactive toward leukocyte-derived GCase. These data indicate that conjugation of iminosugars to the prismarene scaffold is required to achieve the enhanced inhibitory potency observed for the decavalent **PrS­[5]** ^***PIP***^ and **PrS­[5]** ^***PYR***^.

Since in our experience GCase inhibitors with IC_50_ values in the micromolar range (2–100 μM)^57,60,61^ may also act as good PCs, the ability of **PrS­[5]** ^***PIP***^ and **PrS­[5]** ^***PYR***^ to enhance mutant GCase activity in intact cell lines was assayed by coincubating different concentrations of the compounds with fibroblasts derived from Gaucher patients bearing the N370S/RecNcil or the homozygous L444P mutations following a well-established procedure.^61,62^ Notably, decavalent **PrS­[5]** ^***PIP***^ was able to enhance GCase activity by 30% at 50 nM concentration after 4-day incubation in fibroblasts from Gaucher patients bearing the N370S/RecNcil mutations (Figure 2a), while no activity enhancement was observed in cell lines bearing the neuropathic L444P mutation (see Supporting Information). Even more interestingly, **PrS­[5]** ^***PYR***^, completely ineffective in fibroblasts bearing the N370S/RecNcil mutations (see Supporting Information), enhanced GCase activity by 40% at 1.0 μM in fibroblasts bearing the homozygous L444P mutation, which is more refractory to even the most promising PCs such as isofagomine and ambroxol (Figure 2b).

**Figure 2:**
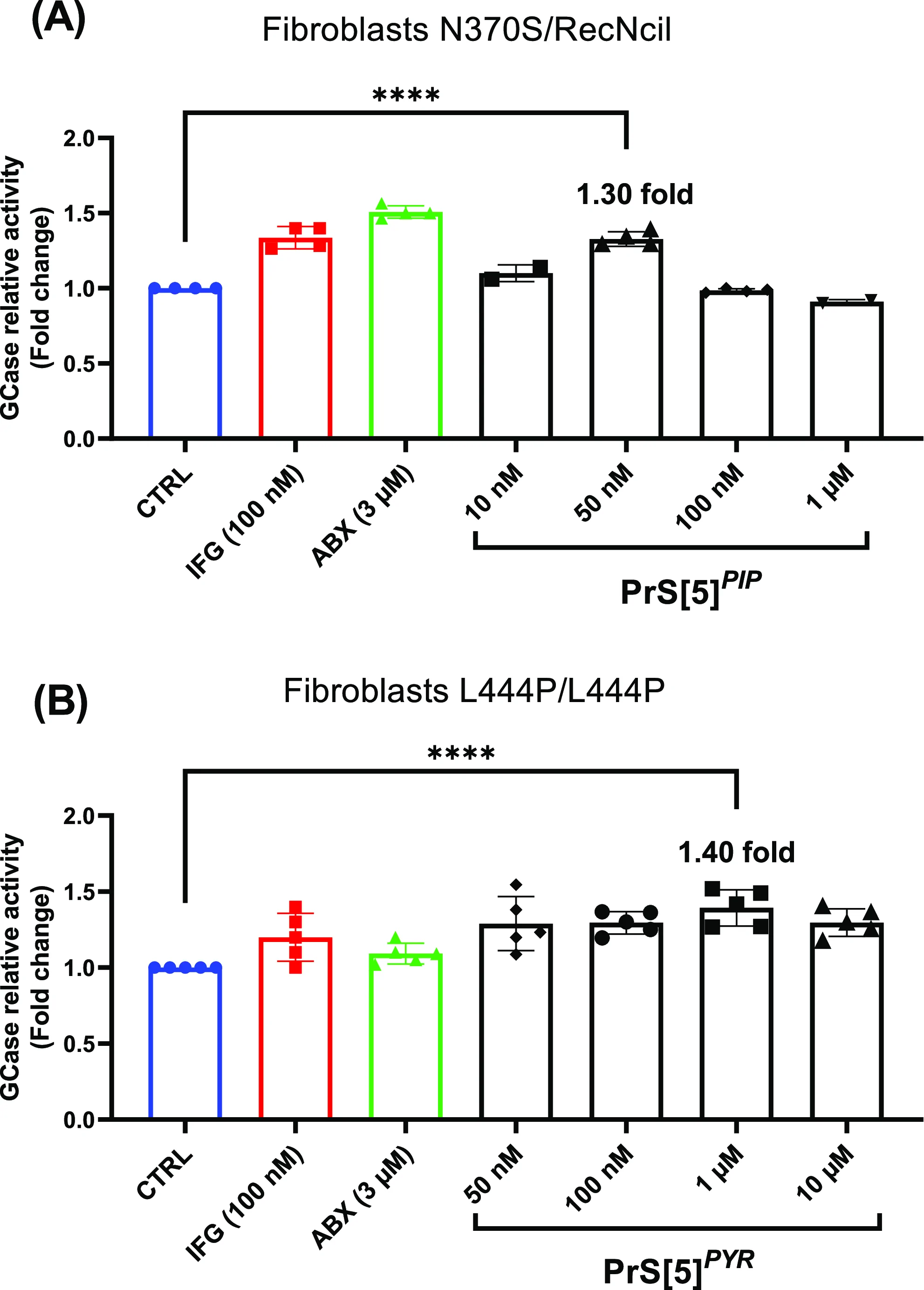
GCase relative activity is reported as the ratio between the GCase activity measured in human fibroblasts derived from (A) GD patients with N370S/RecNcil mutations after 4 days of incubation with compound **PrS­[5]** ^***PIP***^ (black bars) and the corresponding untreated cells (CTRL; blue bar), using IFG (100 nM; red bar) and ABX (3 μM; green bar) as comparison; (B) GD patients with L444P/L444P mutations after 4 days of incubation with compound **PrS­[5]** ^***PYR***^ (black bars) and the corresponding untreated cells (CTRL, blue bar), using IFG (100 nM; red bar) and ABX (3 μM, green bar) as comparison.

The overall data show that, for these decavalent ligands, the nature of the iminosugar covalently linked to the prismarene scaffold drives the rescue of the GCase activity, depending on the harboring mutation: the trihydroxypiperidine iminosugar **PrS­[5]** ^***PIP***^ is more effective toward the N370S mutation, while the DAB-1 pyrrolidine iminosugar **PrS­[5]** ^***PYR***^ unexpectedly enhances L444P mutant GCase, highlighting a mutation-dependent selectivity.

### *In Silico* Studies

With these results in hand, computational studies were performed to gain deeper insights into the possible binding modes of prismarene multivalent clusters with GCase and the role of the prismarene scaffold. For this purpose, we chose to work on the derivative **PrS­[5]** ^***PIP***^ (Figure 3) that shows the most promising results in terms of inhibitory capacity and the greatest multivalent effect.

**Figure 3:**
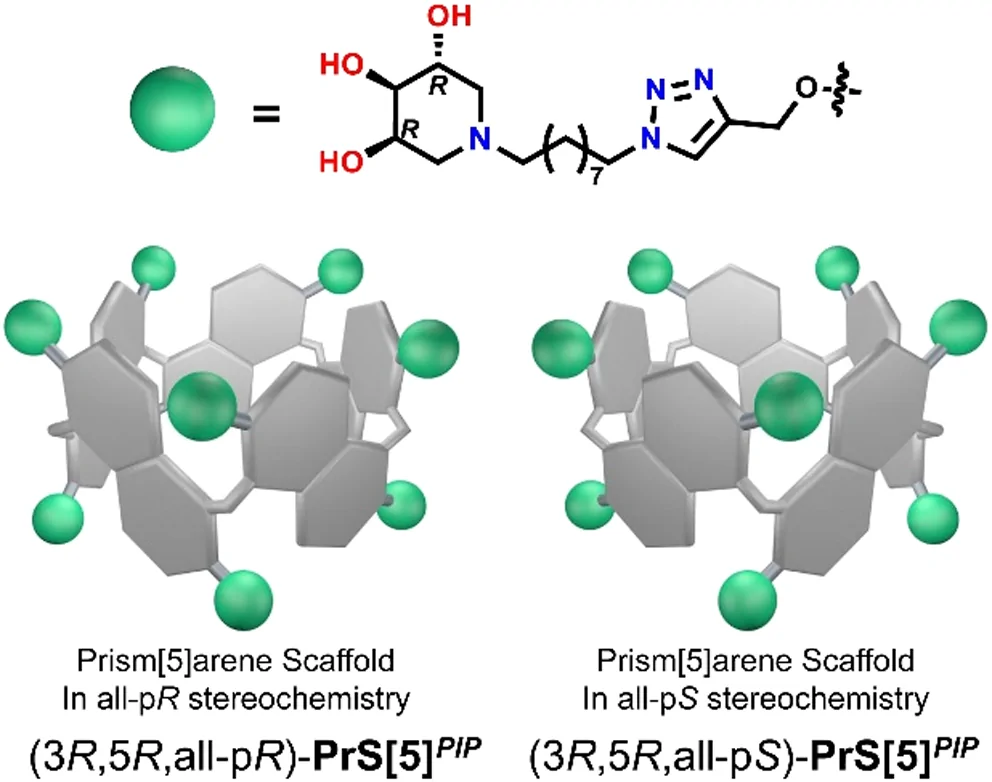
Two diastereoisomers of the derivative **PrS­[5]** ^***PIP***^ investigated in this work.

However, computational investigations of very large molecular systems, such as those reported in this work, are not trivial, and the combination of complementary approaches can provide deeper insight into ligand-substrate interactions. For this reason, both molecular docking and molecular dynamics (MD) simulations were employed using AutoDock VINA (implemented in YASARA) and the YASARA software package, respectively.

As is well-known, prism[5]­arene is a planar chiral macrocycle that exists in two enantiomeric forms, all-p*R* and all-p*S* (Figure 3).^8,9^ The multimerization of chiral enantiopure **PIP-N**_**3**_ in Scheme 1 was carried out using the racemic mixture of **PrS­[5]** ^***Propargyl***^, resulting in a mixture of two diastereoisomers, (3*R*,5*R*,all-p*S*)-**PrS­[5]** ^***PIP***^ and (3*R*,5*R*,all-p*R*)-**PrS­[5]** ^***PIP***^ (Figure 3), which differ only in the stereochemistry of the all-p*R*/all-p*S* configurations of the prism[5]­arene scaffold. The mixture of the two diastereoisomers was then used for biological tests. Thus, the purpose of this computational investigation is to examine the binding modes of both (3*R*,5*R*,all-p*S*)-**PrS­[5]** ^***PIP***^ and (3*R*,5*R*,all-p*R*)-**PrS­[5]** ^***PIP***^ to GCase, and to explore the differences in the complexation behavior through docking and MD simulations (Figures 4 and 5).

**Figure 4:**
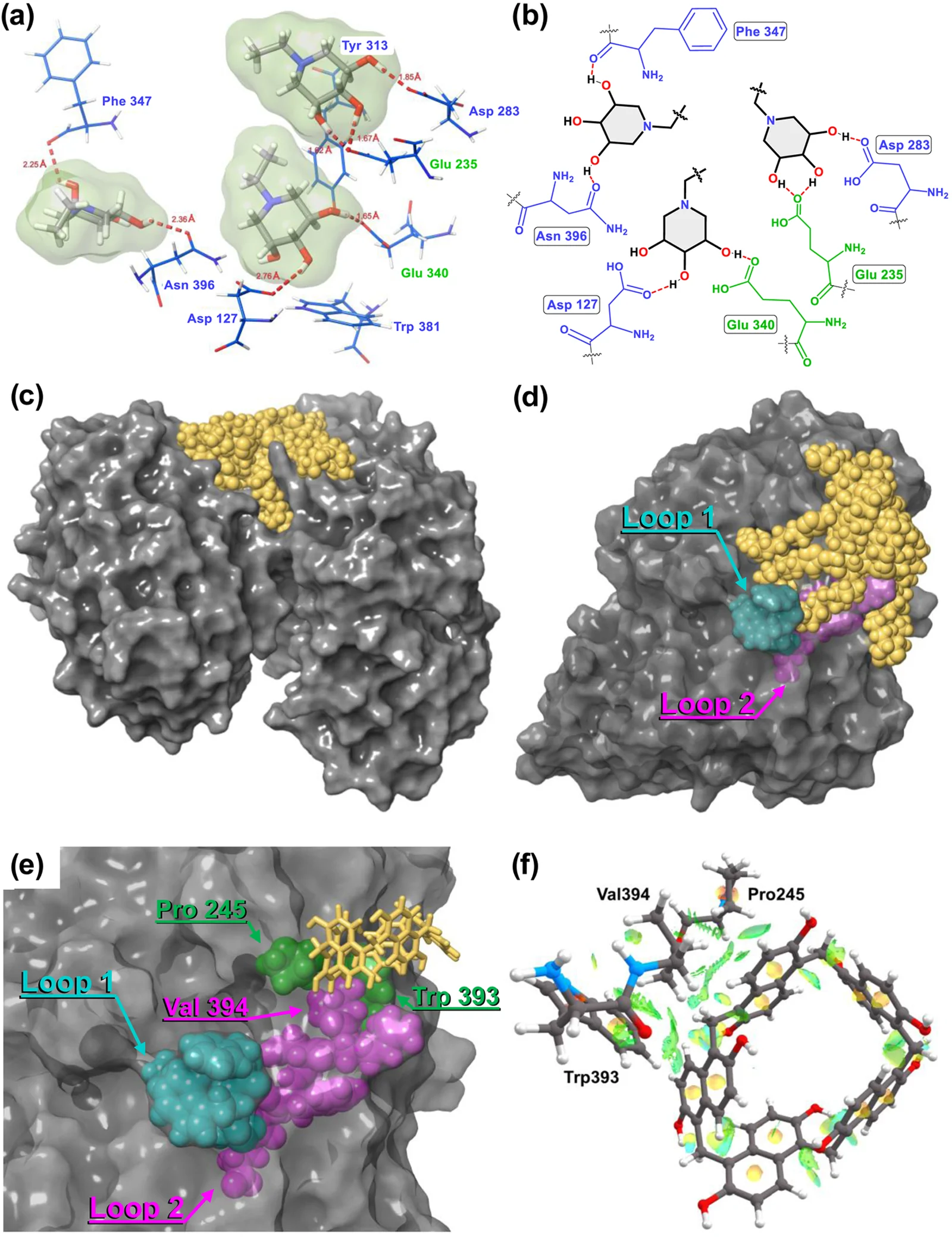
(a) The GCase active site shown in a tube representation, with three PIP units (capped sticks) bound to the active site as obtained from MD simulations; the alkyl chains are omitted for clarity. (b) Chemical representation of the hydrogen-bonding interactions involved in stabilizing the PIP units within the GCase active site. (c, d) 3D model of the binding mode of **PrS­[5]** ^***PIP***^ with GCase; in (d), one of the two monomeric units is removed for clarity, and loop 1 and loop 2 are highlighted in cyan and magenta, respectively. (e) 3D model of the binding mode of the macrocyclic core with the enzyme surface (residue Val394 belongs to loop 2). (f) Gradient RDG isosurfaces (0.30) from NCI analysis based on the promolecular density, highlighting the interactions between the prismarene scaffold and enzyme residues responsible for stabilization above the active site. In both panels (e) and (f), the ten pendants units of **PrS­[5]** ^***PIP***^ are not shown for clarity.

**Figure 5:**
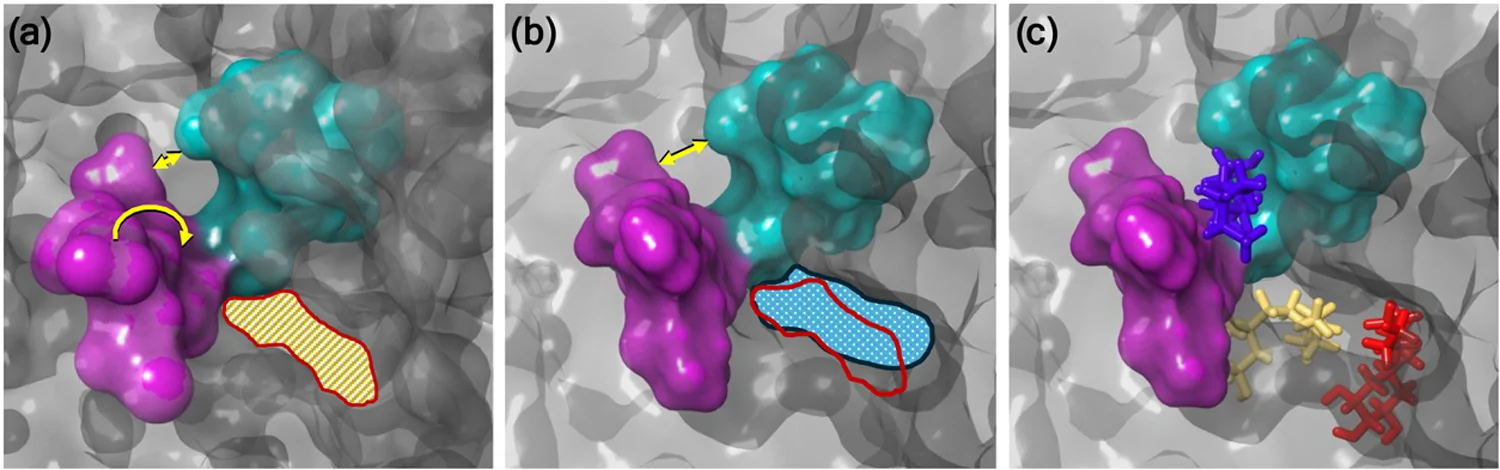
(a) Surface representation of GCase illustrating the conformational change before complexation with **PrS­[5]** ^***PIP***^, and (b) after complexation with the binding cavity prior to binding with **PrS­[5]** ^***PIP***^ highlighted in red. (c) Open conformation in which the PIP units are bound to the active site and interact with loop 1 (cyan) and loop 2 (magenta). In panels (a) and (b) the dashed areas highlight the variation in the size of the cavity providing access to the active site.

GCase exists in a concentration-dependent monomer–dimer equilibrium (Figure 1, top),^49^ and its dimerization is thought to be essential for enzymatic activation, resulting in a more catalytically efficient and stable form under both neutral and lysosomal pH conditions (pH 5.2).^48^ Our investigations were carried out on the human dimer X-ray structure (PDB: 6t13)^49^ of GCase, **(GC)**_**2**_. As reported in the literature, the active-site residues of GCase are located within the TIM-barrel^63^ domain and include the catalytically important residues Glu235 and Glu340 (Figures 4a,b),^64^ with Glu235 playing a particularly significant role in the binding of inhibitors and modulators. In detail, previous studies have shown that Glu340 is the active-site nucleophile and Glu235 serves as the general acid/base during the degradation of glucosylceramide.^65^ It has been demonstrated that catalytic activity of GCase was completely inhibited by covalent binding of inhibitors to Glu340.^65^

Structural analysis of human acid-β-glucosidase (GCase)^64^ reveals that the entrance to the active site is flanked by two flexible surface loops (Figure 5, colored portions): loop 1 (residues 345–349) and loop 2 (residues 394–399).^64^ These loops function as a regulatory lid, existing in a dynamic equilibrium between an open and a closed conformation.^64^ In the open conformation, observed in the crystal structure of GCase covalently bound to the inhibitor conduritol-B-epoxide (CBE),^64^ the active site remains accessible for substrate binding. Conversely, in the closed conformation, the side chains of specific residues—notably Asn396 and Phe397 within loop 2—swing over the active site entrance, physically restricting access.^66^ While the binding of ligands does not appear to induce a global conformational change in the enzyme, it specifically influences the orientation of these loops.

The AutoDock VINA simulations^66,67^ were first carried out to predict the binding modes of (3*R*,5*R*,all-p*S*)-**PrS­[5]** ^***PIP***^ and (3*R*,5*R*,all-p*R*)-**PrS­[5]** ^***PIP***^. In this initial screening, 38 and 40 final poses were saved for the two diastereoisomers. Among the 15 best-ranked poses, the binding energies differed by only 0.70 kcal·mol^–1^ for (3*R*,5*R*,all-p*S*)-**PrS­[5]** ^***PIP***^ and 0.34 kcal·mol^–1^ for (3*R*,5*R*,all-p*R*)-**PrS­[5]** ^***PIP***^ (see Supporting Information). These exhibited comparable contact surface areas between ligand and enzyme, ranging from 1779 to 2058 Å^2^ for (3*R*,5*R*,all-p*R*)-**PrS­[5]** ^***PIP***^ and from 1742 to 2338 Å^2^ for (3*R*,5*R*,all-p*S*)-**PrS­[5]** ^***PIP***^ (see Tables S1 and S2).

Notably, the energy difference between the best-scored poses of the two diastereoisomers was only 0.82 kcal·mol^–1^. Considering the intrinsic accuracy limits of the AutoDock VINA scoring function,^66^ typically estimated to be on the order of 2–3 kcal·mol^–1^ for binding affinity predictions,^65^ this difference cannot be regarded as significant. A Boltzmann analysis at room temperature clarified that both binding modes would be substantially populated, supporting the possible coexistence of all the found poses (considering both diastereoisomers) at the same time.

However, the docking investigation provided valuable clustering information for both (3*R*,5*R*,all-p*S*)-**PrS­[5]** ^***PIP***^ **@(GC)**_**2**_ and (3*R*,5*R*,all-p*R*)-**PrS­[5]** ^***PIP***^ **@(GC)**_**2**_ complexes, revealing a strong preference for binding at the enzyme active site. The (3*R*,5*R*,all-p*R*)-**PrS­[5]** ^***PIP***^ **@(GC)**_**2**_ exhibited a binding mode in 12 of the 15 best-ranked poses (Figure S28c), where the multivalent prismarene was positioned near the residue Trp393 (on the protein surface, Figure 4e,f). Interestingly two 3,4,5-trihydroxypiperidine (PIP) units extend into the active site (vide infra and Figure S34a,b). This represents, to our knowledge, the first evidence of the simultaneous occupancy of the GCase catalytic enzymatic site with two bioactive units. A similar clustering pattern was observed for (3*R*,5*R*,all-p*S*)-**PrS­[5]** ^***PIP***^ **@(GC)**_**2**_, with 13 out of 15 poses showing the insertion of the PIP units into the active site. At this point, the calculations highlighted the contribution of the prismarene scaffold (Figure 4f). As is known, prismarene features large aromatic walls, and the docking study suggested a pairing of these walls with surface protein residues Arg353, Pro245, Val394, and Trp393 (Figures 4f and S33a), which are near the active site (vide infra), in 20 out of the 30 best-ranked poses across both (3*R*,5*R*,all-p*S*)-**PrS­[5]** ^***PIP***^ **@(GC)**_**2**_ and (3*R*,5*R*,all-p*R*)-**PrS­[5]** ^***PIP***^ **@(GC)**_**2**_ complexes (Figure 4f). The docking results indicated a clear convergence of poses toward a common binding arrangement, suggesting a well-defined molecular recognition pattern dominated by multivalent interactions within active site. This convergence also justified the selection of the top-ranked poses (one for each complex) for subsequent molecular dynamics simulations, which were conducted to further investigate the stability and dynamics of these complexes. To perform MD calculations, the systems were placed in a 100 × 100 × 100 Å cubic box and simulated for 10 ns using the AMBER14 force field. The superposition of the **PrS­[5]** ^***PIP***^ structures before (as obtained by docking calculations) and after the MD simulations (Figure S31) showed low root-mean-square (RMS) values for the prismarene scaffold (considering only carbon and oxygen atoms), namely 0.23 Å and 0.16 Å for the (3*R*,5*R*,all-p*S*)-**PrS­[5]** ^***PIP***^ **@(GC)**_**2**_ and (3*R*,5*R*,all-p*R*)-**PrS­[5]** ^***PIP***^ **@(GC)**_**2**_, respectively.

The interactions between the macrocyclic scaffold in both (3*R*,5*R*,all-p*S*)-**PrS­[5]** ^***PIP***^ **@(GC)**_**2**_ and (3*R*,5*R*,all-p*R*)-**PrS­[5]** ^***PIP***^ **@(GC)**_**2**_ complexes and the enzymatic surface (observed in the docking studies – Figure S32) were verified by the analysis of the MD trajectory. Indeed, persistent pairing between the prismarene scaffold and the protein was observed throughout the entire simulation (consistent with the low RMS values of the macrocyclic core). In detail, strong van der Waals interactions (Figure 4f) were calculated between Trp393, Val394, and Pro245 and the aromatic walls of the prismarene scaffold for both (3*R*,5*R*,all-p*S*)-**PrS­[5]** ^***PIP***^ **@(GC)**_**2**_ and (3*R*,5*R*,all-p*R*)-**PrS­[5]** ^***PIP***^ **@(GC)**_**2**_ complexes (Figure 4f). Noncovalent interactions (NCI)^68^ analysis (Figures 4f and S33a) revealed pronounced π···π interactions between Trp393 and the prismarene wall, together with strong van der Waals and C–H···π contacts involving the CH­(CH_3_)_2_ group of Val394 (Figures 4f and S34). This result highlights that the prismarene serves not only as a structural scaffold for the multimerization of biologically active initiators but, due to its extensive aromatic wall, can also provide secondary chemical interactions with the protein, strong enough to stabilize the complex and enhance the strength of the ligand-protein interaction.

Analysis of the minimum-energy structure of the (3*R*,5*R*,all-p*R*)-**PrS­[5]** ^***PIP***^ **@(GC)**_**2**_ (Figures 4 and 5) as obtained by MD simulation indicates that two 3,4,5-trihydroxypiperidine (PIP) units are located within the active site of one of the monomers of the dimeric form of GCase (Figures 4a,b and 5c), while the active site of the other monomer remains unoccupied. Specifically, a hydrogen bond was detected between an OH group of one PIP unit and the active-site nucleophile Glu340, with an OH···O=C distance of 1.65 Å (Figure 4a,b). A second PIP unit can occupy the active site and engage in double hydrogen bonding involving two OH groups of PIP with the C=O group of Glu235 (distances of 1.62 Å and 1.67 Å, OH···O=C, Figures 4a,b and 5c), which serves as the general acid/base during the degradation of glucosylceramide. The two PIP units were held in place by extensive H-bonding interactions with Asp283, and Asp127. The alkyl chains of these PIP units, reminiscent of the two lipophilic chains of the enzyme natural substrates, establish van der Waals contacts with active-site residues, such as Tyr313, Trp348, Gln284, and Phe246. A third PIP unit in Figures 4a,b and 5c (in blue) establishes a hydrogen bonding interactions with the residues Phe347 (2.25 Å, OH···O=C), and Asn396 (2.36 Å, OH···O=C). The corresponding alkyl chain is positioned between Tyr244 and Pro245, in proximity to the triazole groups and Leu241. Similar results were observed for (3*R*,5*R*,all-p*S*)-**PrS­[5]** ^***PIP***^ **@(GC)**_**2**_ (see details in the Supporting Information). In addition to these interactions, other PIP units, located on the rims of the **PrS­[5]** ^***PIP***^ macrocycle, establish hydrogen bonds with amino acids on the surface of the other monomer, thereby sealing the entire dimeric structure. The positioning of two PIP units inside the active site (Figure 5c yellow and red) to establish hydrogen bonding interactions with the catalytically relevant Glu235 and Glu340 is likely facilitated by the conformational movements of the loops surrounding the active site, which open the active site for the entry of the two PIP units. Indeed, as can be observed in Figure 5a,b, the surface around the active site and the two loops exhibit structural variations from a closed (Figure 5a) to an open (Figure 5b) form when comparing the two images in Figure 5a,b, which correspond to the enzyme in its free state and bound to **PrS­[5]** ^***PIP***^.

Furthermore, the number of H-bond interactions between the protein and the ligand was monitored over the course of the simulation. The number of protein–ligand hydrogen bonds ranged from 10 to 26 in the case of the (3*R*,5*R*,all-p*R*)-**PrS­[5]** ^***PIP***^ **@(GC)**_**2**_ complex, whereas a lower average value (approximately 14 hydrogen bonds) was observed for (3*R*,5*R*,all-p*S*)-**PrS­[5]** ^***PIP***^ **@(GC)**_**2**_ (Figure S33).

Importantly, the multivalent binding arrangement remained stable throughout the entire simulation, with the two pendant units persistently engaged within the active site, supporting a robust multivalent recognition mechanism and in excellent accordance with the experimental results. This stability is likely further reinforced by the intrinsic rigidity of the prismarene scaffold, which preorganizes the functional units and promotes sustained interactions with the enzymatic pocket.

## Conclusions

In conclusion, we reported herein the synthesis and characterization of prism[5]­arenes bioconjugated to ten units of 3,4,5-trihydroxypiperidine (PIP) and DAB-1 pyrrolidine (PYR) iminosugars, respectively. Enhanced inhibition of GCase with respect to the monovalent counterpart was observed for both multivalent architectures, but the prism[5]­arene decorated with PIP iminosugars revealed higher affinity for the enzyme.

*In silico* studies performed on the PIP derivatives supported the crucial role of the central aromatic prismarene scaffold in enhancing enzyme interactions, resulting in increased inhibitory potency and showing a significant multivalent effect.

In particular, MD simulations of the PIP derivatives showed multiple secondary interactions on the protein surface facilitated by the rigid prismarene scaffold, which stabilized the dimeric enzyme. Notably, a simultaneous double occupancy of the catalytic site of a monomer by two trihydroxypiperidine units was observed, without affecting the catalytic site of the other monomer.

Due to their inhibitory properties on GCase, the prismarene derivatives were further investigated as pharmacological chaperones to restore enzymatic activity in mutant GCase. In fibroblasts from Gaucher patients, **PrS­[5]** ^***PIP***^ demonstrated a marked enhancement of GCase activity in cells with the N370S mutation, while **PrS­[5]** ^***PYR***^ was effective against the L444P mutation, indicating a selective, mutation-dependent efficacy. Ultimately, these results pave the way for the design of new multivalent modulators for GCase, where the extensive π-electron surface of the central scaffold and the high valency of spatially close substituents can generate increasingly potent inhibitors of GCase.

## Supplementary Material



## Notes

Detailed synthetic procedures for the bioconjugation of prism[5]­arenes, copies of 1D and 2D NMR spectra, high-resolution mass spectra, biochemical characterization, chaperoning activity assays, *in silico* calculations, additional figures and tables (PDF)

## References

[1] Della
Sala P., Del Regno R., Talotta C., Capobianco A., Hickey N., Geremia S., De Rosa M., Spinella A., Soriente A., Spinella A., Neri P., Gaeta C. (2020). Prismarenes:
A New Class of Macrocyclic Hosts Obtained by Templation in a Thermodynamically
Controlled Synthesis. J. Am. Chem. Soc..

[2] Li J., Zhou H. Y., Han Y., Chen C. F. (2021). Saucer­[n]­arenes:
Synthesis, Structure, Complexation, and Guest-Induced Circularly Polarized
Luminescence Property. Angew. Chem., Int. Ed..

[3] Han X.-N., Han Y., Chen C.-F. (2020). Pagoda­[4]­Arene
and *i*-Pagoda­[4]­Arene. J. Am.
Chem. Soc..

[4] Kato K., Kaneda T., Ohtani S., Ogoshi T. (2023). Per-Arylation of Pillar­[n]­arenes:
An Effective Tool to Modify the Properties of Macrocycles. J. Am. Chem. Soc..

[5] Chang R., Ye H., Dong X., Cao X.-Y., Sue A. C.-H. (2025). Biomimetic Metal–Organic
Nanotubular Host for Straight-ChainFatty Acids Recognition. J. Am. Chem. Soc..

[6] Peng Y., Tan M.-L., Ma K.-X., Tong S., Wang M.-X. (2025). Large N-Doped
Zigzag Hydrocarbon Belts from Calixarenes: Synthesis, Structure and
Properties. Angew. Chem., Int. Ed..

[7] Della
Sala P., Del Regno R., Di Marino L., Calabrese C., Palo C., Geremia S., Talotta C., Geremia S., Hickey N., Capobianco A., Neri P., Gaeta C. (2021). An intramolecularly
self-templated synthesis of macrocycles: self-filling effects on the
formation of prismarenes. Chem. Sci..

[8] Della
Sala P., Talotta C., De Rosa M., Superchi S., Santoro E., Geremia S., Hickey N., Fusè M., Abbate S., Mazzeo G., Longhi G., Gaeta C. (2025). Introducing
Prism­[4]­arene: A Macrocycle with Enantiomerically Resolvable Inherent
Chirality and Intriguing Chiroptical Properties. J. Am. Chem. Soc..

[9] Della
Sala P., Calice U., Iuliano V., Geremia S., Hickey N., Belviso S., Summa F. F., Monaco G., Gaeta C., Superchi S. (2024). Chirality Sensing of Cryptochiral Guests with Prism­[n]­arenes. Chem.—Eur. J..

[10] Liang X., Shen Y., Zhou D., Ji J., Wang H., Zhao T., Mori T., Wu W., Yang C. (2022). Chiroptical
induction with prism[5]­arene alkoxy-homologs. Chem. Commun..

[11] Zhang G., Li Z., Pan Z., Zhao D., Han C. (2023). A facile and ef-ficient
preparation of prism[6]­arene and its dual responsive complexation
with 1-adamantane ammonium tetrakis­[3,5-bis­(trifluoromethyl)-phenyl]­borate. New J. Chem..

[12] Del
Regno R., Della Sala P., Santonoceta G. D. G., Neri P., De Rosa M., Talotta C., Sgarlata C., De Simone A., Gaeta C. (2024). Under the Influence of Water: Molecular
Recognition of Organic Hydrophilic Molecules in Water with a Prismarene
Host Driven by Hydration Effects. Chem.—Eur.
J..

[13] Zhai C., Isaacs L. (2022). New Synthetic Route to Water-Soluble
Prism[5]­arene
Hosts and Their Molecular Recognition Properties. Chem.—Eur. J..

[14] Kaur G., Bazan-Bergamino E. A., Zhai C., Ashton S., Isaacs L. (2026). Molecular
recognition properties of water-soluble Prism[5]­arene towards drugs
of abuse. Supramol. Chem..

[15] Xie J., Xi Z., Yang Y., Zhang X., Yang Z., He M. (2023). Computational
investigation of the structures, properties, and host-guest chemistry
of prism­[n]­arenes. Int. J. Quantum Chem..

[16] Zhang G., Cheng C., Li Z., Zhao D., Han C. (2024). Charge-transfer
inclusion complex formation of the tropylium cation with prism[6]­arenes. Org. Biomol. Chem..

[17] Del
Regno R., Campanile G., Neri P., Talotta C., Rescifina A., Sgarlata C., Santonoceta G. D. G., Gaeta C., De Rosa M. (2025). *Carboxylato*-prism­[6]­arene
as a supramolecular catalyst in water: exploiting its deep hydrophobic
cavity for green oxidation of aromatic amines. Chem. Sci..

[18] Zhao K., Zeng L., Zhao J., Yang P., Nie J., Chang Y. (2023). Supra-herbicide based
on sunlight-opened macrocycle gate with reduced
toxicity. J. Ind. Eng. Chem..

[19] Pei D., Guo W., Liu P., Xue T., Meng X., Dhu X., Nie J., Cheng Y. (2022). Prism­[5]­arene-based
nonporous adaptive crystals for
the capture and detection of aromatic volatile organic compounds. Chem. Eng. J..

[20] Vinodh M., Abdeljaber N. O., Alipoura F. H., Al-Azemi T. F. (2025). Prism­[*n*]­arene-alkyl
dibromide (n = 5, 6) synergy: molecular affinity in
the solid state. CrystEngComm.

[21] Choi, S.-K. Synthetic Multivalent Molecules: Concepts and Biomedical Applications; John Wiley & Sons, Inc.: Hoboken, NJ, 2004.

[22] Wittmann V., Pieters R. J. (2013). Bridging Lectin Binding Sites by Multivalent Carbohydrates. Chem. Soc. Rev..

[23] González-Cuesta M., Ortiz Mellet C., García Fernández J. M. (2020). Carbohydrate
supramolecular chemistry: beyond the multivalent effect. Chem. Commun..

[24] Gouin S. G. (2014). Multivalent
Inhibitors for Carbohydrate- Processing Enzymes: Beyond the “Lock-and-Key”
Concept. Chem. - Eur. J..

[25] Matassini C., Parmeggiani C., Cardona F., Goti A. (2016). Are enzymes sensitive
to the multivalent effect? Emerging evidence with glycosidases. Tetrahedron Lett..

[26] Compain P. (2019). Multivalent
Effect in Glycosidase Inhibition: The End of the Beginning. Chem. Rec..

[27] Compain, P. ; Martin, O. R. Iminosugars: From Synthesis to Therapeutic Applications; Wiley: New York, 2007.

[28] Brissonnet Y., Ortiz Mellet C., Morandat S., Garcia Moreno M. I., Deniaud D., Matthews S. E., Vidal S., Šesták S., El Kirat K., Gouin S. G. (2013). Topological effects and binding modes
operating with multivalent iminosugar-based glycoclusters and mannosidases. J. Am. Chem. Soc..

[29] Rísquez-Cuadro R., Matsumoto R., Ortega-Caballero F., Nanba E., Higaki K., García Fernández J. M., Ortiz Mellet C. (2019). Pharmacological
Chaperones for the Treatment of α-Mannosidosis. J. Med. Chem..

[30] Della
Sala P., Vanni C., Talotta C., Di Marino L., Matassini C., Goti A., Neri P., Šesták S., Cardona F., Gaeta C. (2021). Multivalent resorcinarene clusters
decorated with DAB-1 inhitopes: targeting Golgi α-mannosidase
from Drosophila melanogaster. Org. Chem. Front..

[31] Howard E., Cousido-Siah A., Lepage M. L., Schneider J. P., Bodlenner A., Mitschler A., Meli A., Izzo I., Alvarez A., Podjarny A., Compain P. (2018). Structural Basis of
Outstanding Multivalent Effects in Jack Bean α-Mannosidase Inhibition. Angew. Chem., Int. Ed..

[32] Matassini C., Marradi M., Cardona F., Parmeggiani C., Robina I., Moreno-Vargas A. J., Penadés S., Goti A. (2015). Gold nanoparticles are suitable cores for building tunable iminosugar
multivalency. RSC Adv..

[33] Hottin A., Wright D. W., Moreno-Clavijo E., Moreno-Vargas A. J., Davies G. J., Behr J.-B. (2016). Exploring the divalent
effect in
fucosidase inhibition with stereoisomeric pyrrolidine dimers. Org. Biomol. Chem..

[34] Carmona A. T., Carrion-Jimenez S., Pingitore V., Moreno-Clavijo E., Robina I., Moreno-Vargas A. J. (2018). Harnessing pyrrolidine iminosugars
into dimeric structures for the rapid discovery of divalent glycosidase
inhibitors. Eur. J. Med. Chem..

[35] Platt F. M., d’Azzo A., Davidson B. L., Neufeld E. F., Tifft C. J. (2018). Lysosomal
storage diseases. Nat. Rev. Dis. Primers.

[36] Martínez-Bailén M., Carmona A. T., Cardona F., Matassini C., Goti A., Kubo M., Kato A., Robina I., Moreno-Vargas A. J. (2020). Synthesis of multimeric pyrrolidine
iminosugar inhibitors
of human -glucocerebrosidase and-galactosidase a: First example of
a multivalent enzyme activity enhancer for Fabry disease. Eur. J. Med. Chem..

[37] Decroocq C., Rodríguez-Lucena D., Ikeda K., Asano N., Compain P. (2012). Cyclodextrin-based iminosugar click
clusters: The first
examples of multivalent pharmacological chaperones for the treatment
of lysosomal storage disorders. ChemBioChem.

[38] Martínez-Bailén M., Clemente F., Matassini C., Cardona F. (2022). GCase enhancers: a
potential therapeutic option for Gaucher disease and other neurological
disorders. Pharmaceuticals.

[39] Clemente F., Davighi M. G., Matassini C., Cardona F., Goti A., Morrone A., Paoli P., Tejero T., Merino P., Cacciarini M. (2023). Light-triggered
Control of Glucocerebrosidase Inhibitors:
Towards Photoswitchable Pharmacological Chaperones. Chem. -Eur. J..

[40] Tran M. L., Borie-Guichot M., Garcia V., Oukhrib A., Genisson Y., Levade T., Ballereau S., Turrin C.-O., Dehoux C. (2023). Phosphorus
Dendrimers for Metal-Free Ligation: Design of Multivalent Pharmacological
Chaperones against Gaucher Disease. Chem. -Eur.
J..

[41] Borie-Guichot M., Tran L. M., Garcia V., Oukhrib A., Rodriguez F., Turrin C.-O., Levade T., Genisson Y., Ballereau S., Dehoux C. (2024). Multivalent pyrrolidines acting as
pharmacological
chaperones against Gaucher disease. Bioorg.
Chem..

[42] Buco F., Clemente F., Morrone A., Vanni C., Moya S. E., Cardona F., Goti A., Marradi M., Matassini C. (2025). Multivalent
GCase Enhancers: Synthesis and Evaluation of Glyco-Gold Nanoparticles
Decorated with Trihydroxypiperidine Iminosugars. Bioconjugate Chem..

[43] Davighi M. G., Clemente F., Morano A., Mangiavacchi F., Cardona F., Goti A., Paoli P., Morrone A., Nieto-Fabregat F., Marchetti R., Matassini C. (2026). Simultaneous
Grafting of 3,4,5-Trihydroxypiperidine Iminosugars onto Multivalent
Scaffolds via Double Reductive Amination Provides New GCase Inhibitors. Chem. -Eur. J..

[44] D’Adamio G., Matassini C., Parmeggiani C., Catarzi S., Morrone A., Goti A., Paoli P., Cardona F. (2016). Evidence for a multivalent
effect in inhibition of sulfatases involved in lysosomal storage disorders. RSC. Adv..

[45] Matassini C., D’Adamio G., Vanni C., Goti A., Cardona F. (2019). Studies for
the multimerization of DAB-1-based iminosugars through iteration of
the nitrone cycloaddition/ring-opening/allylation sequence. Eur. J. Org. Chem..

[46] Matassini C., Vanni C., Goti A., Morrone A., Marradi M., Cardona F. (2018). Multimerization of DAB-1 onto Au GNPs affords new potent
and selective *N*-acetylgalactosamine-6-sulfatase (GALNS)
inhibitors. Org. Biomol. Chem..

[47] Davighi M. G., Clemente F., D’Adamio G., Martínez-Bailén M., Morano A., Goti A., Morrone A., Matassini C., Cardona F. (2025). Exploring Multivalent
Architectures for Binding and
Stabilization of N-Acetylgalactosamine 6-Sulfatase. Molecules.

[48] Grabowski G. A. (2008). Phenotype,
diagnosis, and treatment of Gaucher’s disease. Lancet.

[49] Benz J., Rufer A. C., Huber S., Ehler A., Hug M., Topp A., Guba W., Hofmann E. C., Jagasia R., Rodríguez Sarmiento R. M. (2021). Novel β-Glucocerebrosidase
Activators That Bind to a New Pocket at a Dimer Interface and Induce
Dimerization. Angew. Chem., Int. Ed..

[50] Smith L., Schapira A. H. V. (2022). GBA Variants
and Parkinson Disease: Mechanisms and
Treatments. Cells.

[51] Hertz E., Chen Y., Sidransky E. (2024). Gaucher Disease Provides a Unique
Window into Parkinson Disease Pathogenesis. Nat. Rev. Neurol..

[52] Li Q., Bi Z., Liang W., Yin S., Li H., Liang Z., Wu M., Quan J., Li C. (2025). Effects of
glucocerebrosidase gene
variations on the risk of Parkinson’s disease dementia: a meta-analysis. Front Aging Neurosci..

[53] Kuo S.-H., Tasset I., Cheng M. M., Diaz A., Pan M.-K., Lieberman O. J., Hutten S. J., Alcalay R. N., Kim S., Ximénez-Embún P., Fan L., Kim D., Ko H. S., Yacoubian T., Kanter E., Liu L., Tang G., Muñoz J., Sardi S. P., Li A., Gan L., Cuervo A. M., Sulzer D. (2022). Mutant Glucocerebrosidase Impairs
α-Synuclein Degradation by Blockade of Chaperone-Mediated Autophagy. Sci. Adv..

[54] Sánchez-Fernández E. M., Garcia Fernandez J. M., Ortiz Mellet C. (2016). Glycomimetic-based pharmacological
chaperones for lysosomal storage disorders: lessons from Gaucher,
GM1-gangliosidosis and Fabry diseases. Chem.
Commun..

[55] Pereira D. M., Valentão P., Andrade P. B. (2018). Tuning protein folding in lysosomal
storage diseases: the chemistry behind pharmacological chaperones. Chem. Sci..

[56] Del
Regno R., Della Sala P., Picariello D., Talotta C., Spinella A., Neri P., Gaeta C. (2021). *per*-Hydroxylated Prism­[n]­arenes: Supramolecularly Assisted Demethylation
of Methoxy-Prism[5]­arene. Org. Lett..

[57] Vanni C., Clemente F., Paoli P., Morrone A., Matassini C., Goti A., Cardona F. (2022). 3,4,5-Trihydroxypiperidine
Based
Multivalent Glucocerebrosidase (GCase) Enhancers. ChemBioChem.

[58] Parmeggiani C., Catarzi S., Matassini C., D’Adamio G., Morrone A., Goti A., Paoli P., Cardona F. (2015). Human acid
β-glucosidase inhibition by carbohydrate derived iminosugars:
towards new pharmacological chaperones for Gaucher disease. ChemBioChem.

[59] Kanfar N., Bartolami E., Zelli R., Marra A., Winum J.-Y., Ulrich S., Dumy P. (2015). Emerging trends in enzyme inhibition
by multivalent nanoconstructs. Org. Biomol.
Chem..

[60] Clemente F., Matassini C., Faggi C., Giachetti S., Cresti C., Morrone A., Paoli P., Goti A., Martinez-Bailen M., Cardona F. (2020). Glucocerebrosidase (GCase) activity
modulation by 2-Alkyl trihydroxypiperidines: inhibition and pharmacological
chaperoning. Bioorg. Chem..

[61] Davighi M. G., Clemente F., Matassini C., Cacciarini M., Tanini D., Goti A., Morrone A., Paoli P., Cardona F. (2025). Acetal functionalized iminosugars
for targeting β-glucocerebrosidase
modulation. Eur. J. Med. Chem..

[62] Li H.-Y., Chen W.-A., Lin H.-Y., Tsai C.-W., Chiu Y.-T., Yun W.-Y., Lee N.-C., Chien Y.-H., Hwu W.-L., Cheng W.-C. (2024). A practical synthesis
of nitrone-derived C5a-functionalized
isofagomines as protein stabilizers to treat Gaucher disease. Commun. Chem..

[63] Dobert J. P., Schäfer J.-H., Dal Maso T., Ravindran P., Huard D. J. E., Socher E., Schildmeyer L. A., Lieberman R. L., Versées W., Moeller A., Zunke F., Arnold P. (2025). Cryo-TEM Structure
of β-Glucocerebrosidase in
Complex with Its Transporter LIMP-2. Nat. Commun..

[64] Lieberman R. L., Wustman B. A., Huertas P., Powe A. C., Pine C. W., Khanna R., Schlossmacher M. G., Ringe D., Petsko G. A. (2007). Structure of acid
b-glucosidase with
pharmacological chaperone provides insight into Gaucher disease. Nat. Chem. Biol..

[65] Premkumar L., Sawkar A. R., Boldin-Adamsky S., Toker L., Silman I., Kelly J. W., Futerman A. H., Sussman J. L. (2005). X-ray structure
of human acid-beta-glucosidase covalently bound to conduritol-B-epoxide.
Implications for Gaucher disease. J. Biol. Chem..

[66] Kitchen D. B., Decornez H., Furr J. R., Bajorath J. (2004). Docking and Scoring
in Virtual Screening for Drug Discovery: Methods and Applications. Nat. Rev. Drug Discovery.

[67] Trott O., Olson A. J. (2010). AutoDock Vina: Improving the Speed and Accuracy of
Docking with a New Scoring Function, Efficient Optimization, and Multithreading. J. Comput. Chem..

[68] Johnson E.
R., Keinan S., Mori-Sánchez P., Contreras-García J., Cohen A. J., Yang W. (2010). Revealing Noncovalent Interactions. J. Am.
Chem. Soc..

