# Supplementary material for: Prism[5]arene Iminosugar Bioconjugates Are Potent Multivalent Inhibitors and Mutation Specific Pharmacological Chaperones of Glucocerebrosidase (GCase) Activity
Source: Bioconjug Chem. 2026 Aug 3;37(9):1857–66. doi: 10.1021/acs.bioconjchem.6c00273 (PMC13587594; doi:10.1021/acs.bioconjchem.6c00273)
Supplement: Supplementary file 1 [file bc6c00273_si_001.pdf]

Supplementary Information

Prism[5]arene Iminosugar Bioconjugates Are Potent Multivalent Inhibitors and Mutation Specific Pharmacological Chaperones of Glucocerebrosidase (GCase) Activity

Anna Palmieri,[a,∫] Francesca Clemente,[b,∫] Maria Manuela Ignoto,[a] Fabio Buonsenso,[b] Miriana Attanasio,[a] Margherita De Rosa,[a] Carmen Talotta,[a] Paolo Della Sala,[a,*] Rocco del Regno,[a] Veronica Iuliano,[a] Amelia Morrone,[c,d] Francesca Cardona,[b] Andrea Goti,[b] Camilla Matassini,[b,*] Carmine Gaeta[a,*]

a Laboratory of Supramolecular Chemistry, Dipartimento di Chimica e Biologia "A. Zambelli", Università di Salerno, Via Giovanni Paolo II 132, 84084 Fisciano, Salerno, Italy. b Dipartimento di Chimica "Ugo Schiff", Università degli Studi di Firenze, via della Lastruccia 3-13, 50019 Sesto Fiorentino, FI, Italy. c Laboratory of Molecular Biology of Neurometabolic Diseases, Neuroscience Department, Meyer Children's Hospital, IRCCS, Viale Pieraccini 24, 50139 Firenze, Italy.d Department of Neurosciences, Psychology, Drug Research and Child Health, University of Florence, Viale Pieraccini 24, 50139 Firenze, Italy.

**Table of Contents**	**Pages**
General Experimental Details	**S2**
Procedure for the Synthesis of (3-Bromoprop-1-yn-1-yl)triisopropylsilane (propargyl bromide-TIPS)	**S2-S3**
General procedures for the Synthesis of the Bioconjugation of prism[5]arenes	**S3-S5**
Copies of 1D and 2D NMR Spectra and HR Mass Spectra	**S6-S20**
Biochemical Characterization	**S21-S26**
Chaperoning activity assays	**S27-S29**
In silico Calculation	**S30-S36**
References	**S37-S38**

General Experimental Details

All chemical reagents were used without further purification and were purchased from Merck, TCI, ABCR, and Fluorochem. Reaction temperatures were measured externally of reaction flask and the reaction progress were monitored by TLC on Merck silica gel (0.25 mm) and visualized by UV light at 254 nm, or by spraying with H_2SO_4-$Ce(SO_4)_2$.

NMR spectra were recorded on a Bruker Avance-600 [600 MHz (^1H) and 150 MHz (^{13}C)], and Avance-400 [400 MHz (^1H) and 100 MHz (^{13}C)]. Chemical shifts are reported relative to the residual solvent peak.[1] Standard pulse programs provided by the manufacturer were used for 2D COSY (cosygpqf), and 2D HSQC (hsqcedetgpsisp2.2) experiments. Structural assignments were made with additional information from gCOSY, and gHSQC experiments. HR MALDI mass spectra were recorded using a Bruker Solaris XR Fourier transform ion cyclotron resonance mass spectrometer equipped with a 7T refrigerated actively shielded superconducting magnet. All samples were recorded in MALDI mode (four laser shots were used for each scan) and were prepared by mixing 10 μL of analyte in dichloromethane or methanol (1 mg/mL) with 10 μL of a 2,5-dihydroxybenzoic acid solution (10 mg/mL in methanol) and the samples were ionized in positive ion mode. The mass spectra were calibrated externally using a methanolic solution of NaTFA as standard and linear calibration was applied.

Derivatives *per*-hydroxylated prism[5]arene (**PrS[5]*^H^***),[2] (3-Bromoprop-1-yn-1-yl)triisopropylsilane **(propargyl bromide-TIPS)**[3,4] (3*R*,5*R*)-1-(9-azidononyl)-3,4,5-trihydroxypiperidine (**PIPN$_3$**)[5] and **(**2*R*,3*R*,4*R*)-1-[6-azido)hexyl]-3-hydroxymethylhexahydro-1Hpyrrolidine-1,2-diol (**PYRN$_3$**)[6] were synthesized according to reported procedures, and their spectral data are consistent with those reported in the literature.

Synthesis of (3-Bromoprop-1-yn-1-yl)triisopropylsilane (propargyl bromide-TIPS)[3,4]

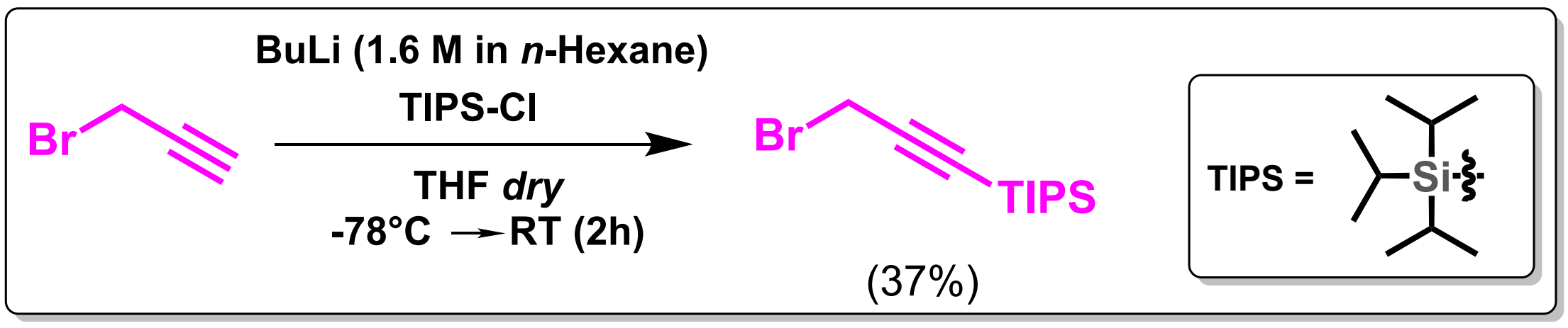

Under a nitrogen atmosphere, propargyl bromide (80 wt.% in toluene, 1.94 mL, 17.50 mmol, 1.0 equiv) was dissolved in dry THF (50 mL, 0.35 M). The reaction mixture was cooled to −78 °C, and *n*-BuLi (1.60 M in *n*-hexane, 10.90 mL, 17.50 mmol, 1.0 equiv) was added dropwise. After stirring for 10 min, triisopropylsilyl chloride (TIPS-Cl, 3.74 mL, 17.50 mmol, 1.0 equiv) was added dropwise. The reaction mixture was allowed to warm to room temperature and stirred for an additional 2 h. The reaction was quenched with a 10% aqueous NH_4Cl solution (100 mL) and diluted with CH_2Cl_2 (70 mL). The layers were separated, and the aqueous phase was extracted with CH_2Cl_2 (2 x 70 mL). The combined organic extracts were dried over anhydrous Na_2SO_4, filtered, and concentrated under reduced pressure. The crude was purified on silica gel - chromatographic column (*n*-

hexane) to afford propargyl bromide - TIPS in 37% yield (1.78 g, 6.47 mmol) as a colourless oil. The ^{1}H and ^{13}C NMR spectra agreed with those reported in the literature.[4]

Bioconjugation of Prism[5]arene with 3,4,5-Trihydroxypiperidine (PIP) and DAB-1 Pyrrolidine Iminosugar Derivative (PYR) via Copper(I)-Catalyzed Azide-Alkyne Cycloaddition (CuAAC)

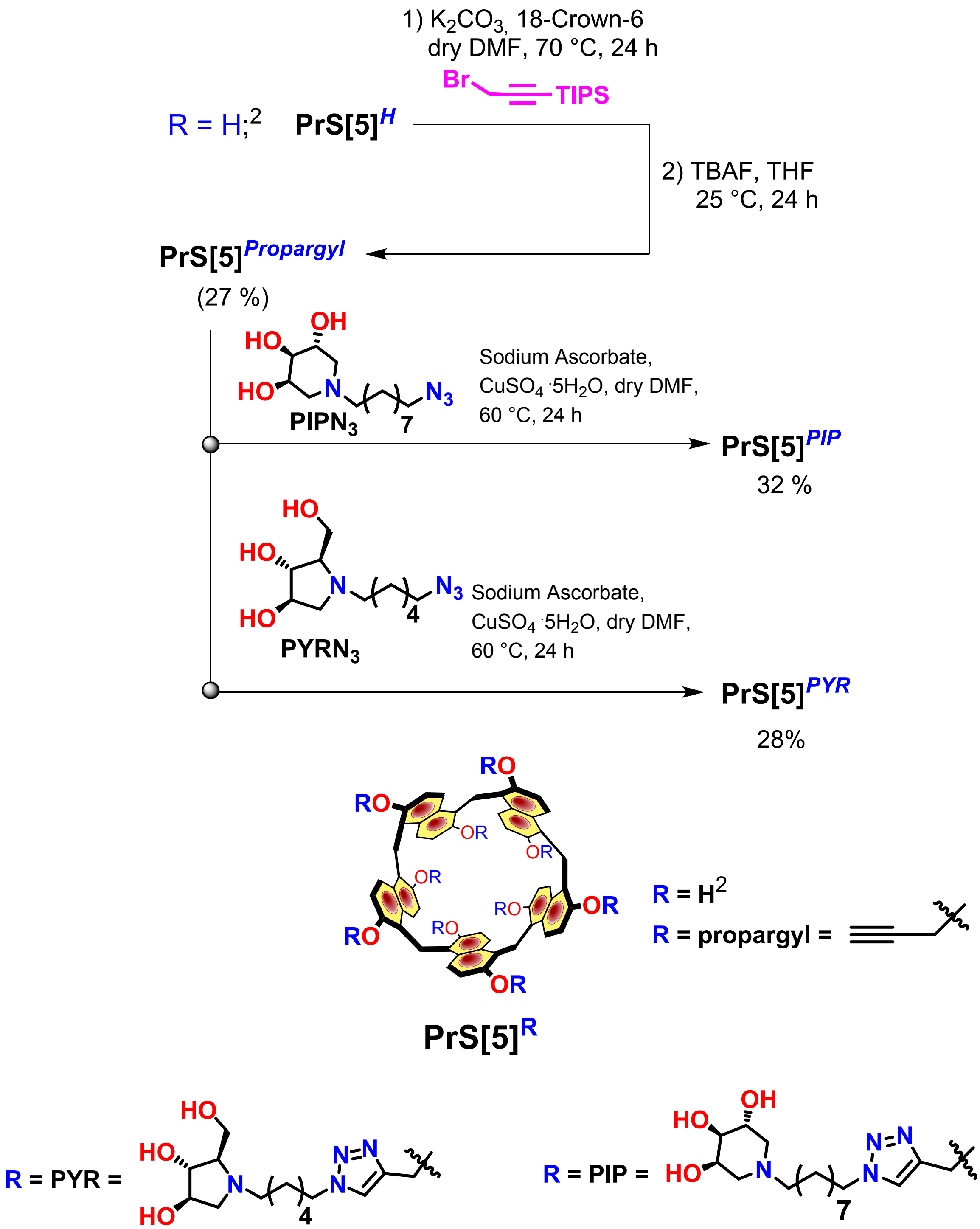

Synthesis of PrS[5]*Propargyl*: Under a nitrogen atmosphere, **PrS[5]*H*** (0.10 g, 0.12 mmol), anhydrous K_2CO_3 (0.49 g, 3.60 mmol, 30 equiv), and 18-crown-6 (0.95 g, 3.60 mmol, 30 equiv) were dissolved in dry DMF (12.80 mL, 9.10 mM). The reaction mixture was stirred at 70 °C for 1 h, then cooled to room temperature, and propargyl bromide-TIPS (1.32 g, 4.80 mmol, 40 equiv) was added. The reaction mixture was heated to 70 °C and stirred for 23 h. Then, the reaction mixture was diluted with H_2O (30 mL) and CH_2Cl_2 (15 mL) was added. The layers were separated, and the aqueous phase was extracted with CH_2Cl_2 (2 × 15 mL). The combined organic layers were dried using anhydrous Na_2SO_4, filtered, and concentrated under reduced pressure to afford a brown crude product, which was used in the next step without further purification. The crude material was dissolved in THF (130 mL), and TBAF (1.0 M in THF, 3.00 mL, 2.99 mmol) was added. The reaction mixture was stirred at room temperature for 24 h and then the reaction was quenched by addition of H_2O (100 mL) and CH_2Cl_2 (70 mL). The organic layer was separated, dried using anhydrous Na_2SO_4, filtered, and concentrated under reduced pressure. The crude product was purified by silica gel column chromatography (*n*-hexane/CH_2Cl_2 = 85:15) to afford **PrS[5]*Propargyl*** as a white solid (27% yield, 40.19 mg, 32.40 μmol). M.p.: > 308 °C dec. ^1H NMR (CD_2Cl_2, 600 MHz, 298 K): δ 7.98 (d, 10H, *J* = 9.2 Hz, Ar-H), 7.08 (d, 10H, *J* = 9.2 Hz, Ar-H), 4.74 (s, 10H, $ArCH_2Ar$), 4.43 (broad, 20H, -OC*H*$_2$C≡CH), 2.36 (s, 10H, -OCH_2C≡C*H*).$^{13}C\{1H\}$ NMR (CD_2Cl_2, 150 MHz, 298 K): δ 151.7, 130.1, 125.3, 124.7, 115.3, 80.0, 75.3, 57.4, 22.2. HRMS (FT-ICR MALDI) *m*/*z* $[M]^+$ calcd for $C_{86}H_{60}O_{10}$: 1240.4186; found: 1240.4142

Synthesis of PrS[5]*PIP*: PrS[5]*Propargyl* (8.00 mg, 6.45 μmol), **$PIPN_3$** (19.39 mg, 67.73 μmol, 10.5 equiv), and $CuSO_4\cdot 5\cdot H_2O$ (0.81 mg, 3.23 μmol, 0.5 equiv) were dissolved in DMF (200 μL, 32.25 mM) in a reaction vial. Sodium ascorbate (1.28 mg, 6.45 μmol, 1.0 equiv) was dissolved in H_2O (50 μL) and added to the reaction mixture. The resulting solution was stirred at 60 °C for 24 h. The solvent was removed under reduced pressure, and the crude product was purified by dialysis (MWCO 1000 Da) over 3 days to remove residual salts, affording **PrS[5]*PIP*** as a pale yellow solid (32% yield, 8.74 mg, 2.06 μmol). M.p.: > 315 °C dec. ^1H NMR (DMF-d_7, 600 MHz, 298 K): *δ* 8.54-8.42 (*overlapped*, Ar-*H*, and *H*-triazole, 20H), 7.35 (*broad*, Ar-*H*, 10H), 5.42-5.35 (*broad,* -OC*H*$_2$-triazole, 20H), 4.59-4.53 (*overlapped*, -ArC*H*$_2$Ar and -C*H*$_2$-triazole, 30H), 4.05 (overlapped to the signal of H_2O, -C*H*$_2$, 10H), 3.79 (overlapped to the signal of H_2O, -C*H*OH, 30H), 3.45 (*broad*, -C*H*$_2$*,* 10H), 2.68-2.61 (*broad*, -C*H*$_2$*,* 10H), 2.33 (*overlapped*, -C*H*$_2$*,* 30H), 1.97-1.87 (*broad*, -C*H*$_2$*,* 20H), 1.41 (*broad*, -C*H*$_2$*,* 20H), 1.26-1.19 (*overlapped*, -C*H*$_2$*,* 100H). $^{13}C\{^1H\}$ NMR (DMF-d_7, 150 MHz, 298 K): *δ* 150.9, 143.7, 129.9, 125.3, 124.8, 124.5, 114.6, 77.3, 73.7, 68.7, 67.6, 62.7, 57.8, 56.2, 50.0, 30.4, 28.9, 27.2, 26.6, 26.3, 21.0. HRMS (FT-ICR MALDI) m/z $[M]^+$ calcd for $C_{225}H_{340}N_{40}O_{40}^+$: 4244.5801; found: 4244.5887.

Synthesis of PrS[5]*PYR*: PrS[5]*Propargyl* (8.00 mg, 6.45 μmol, 1.0 equiv), **$PYRN_3$** (17.49 mg, 67.73 μmol, 10.5 equiv), and $CuSO_4\cdot 5\cdot H_2O$ (0.81 mg, 3.23 μmol, 0.5 equiv) were dissolved in DMF (200 μL, 32.25 mM) in a reaction vial. Sodium ascorbate (1.28 mg, 6.45 μmol, 1.0 equiv) was dissolved in H_2O (50 μL) and added to the reaction mixture. The resulting solution was stirred at 60 °C for 24 h. The solvent was removed under reduced pressure, and the crude product was purified by dialysis (MWCO 1000 Da) over 3 days to remove residual salts, affording **PrS[5]*PYR*** as a pale yellow solid (28% yield, 6.92 mg, 1.81 μmol). M.p.: > 305 °C dec. ^1H NMR (DMSO-d_6, 600 MHz, 298 K): *δ* 8.44-8.37 (*overlapped*, Ar-*H* and *H*-triazole, 20H), 7.29 (broad, Ar-*H*, 10H), 5.33 (*broad*, -OC*H*$_2$-triazole, 20H), 4.86 (*broad*, -$CHCH_2$O*H*, 10H), 4.73 (*broad*, -$CHCH_2$O*H*, 10H), 4.50-4.36 (*overlapped*, -ArC*H*$_2$Ar and -C*H*$_2$-triazole, 30H), 3.76 (*broad*, -C*H*OH, 10H), 3.65 (*broad*, -C*H*OH, 10H),

3.52-3.46 (*broad*, -CHCH_2OH, 10H), 3.35 (overlapped to the signal of H_2O, -CHCH_2OH, 10H), 2.80 (*broad*, -CH_2, 10H), 2.71 (*broad*, -CH_2, 10H), 2.46 (overlapped to the residual DMSO signal, -CH_2, 10H), 2.27 (*broad*, -C*H*OH, 10H), 2.17 (*broad*, -CH_2, 10H), 1.82 (*broad*, -CH_2, 20H), 1.29-1.23 (overlapped to the signal of grease, -CH_2, 60H). $^{13}C\{^1H\}$ NMR (DMSO-d_6, 150 MHz, 298 K): δ 150.5, 142.9, 129.6, 124.6, 123.2, 122.8, 115.2, 78.9, 75.6, 73.0, 62.6, 61.4, 59.3, 54.8, 49.4, 29.0, 27.6, 26.2, 25.8, 22.1. HRMS (FT-ICR MALDI) m/z $[M+K]^+$ calcd for $C_{195}H_{280}KN_{40}O_{40}^+$: 3864.0852; found: 3864.2677.

Copies of 1D and 2D NMR Spectra and HR Mass Spectra

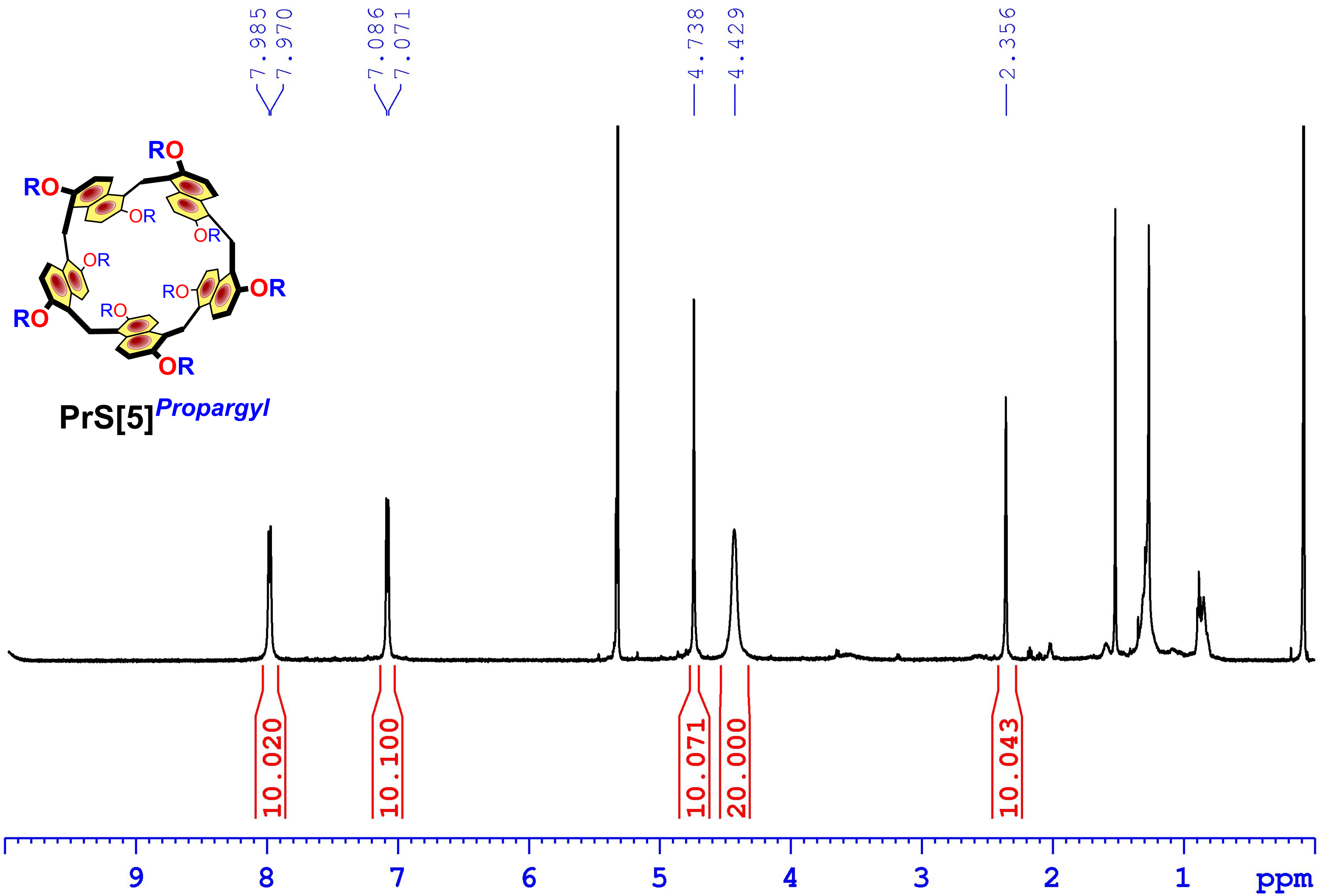

Figure S1: ^{1}H NMR spectrum of derivative **PrS[5]**^{Propargyl} (CD_2Cl_2, 600 MHz, 298 K).

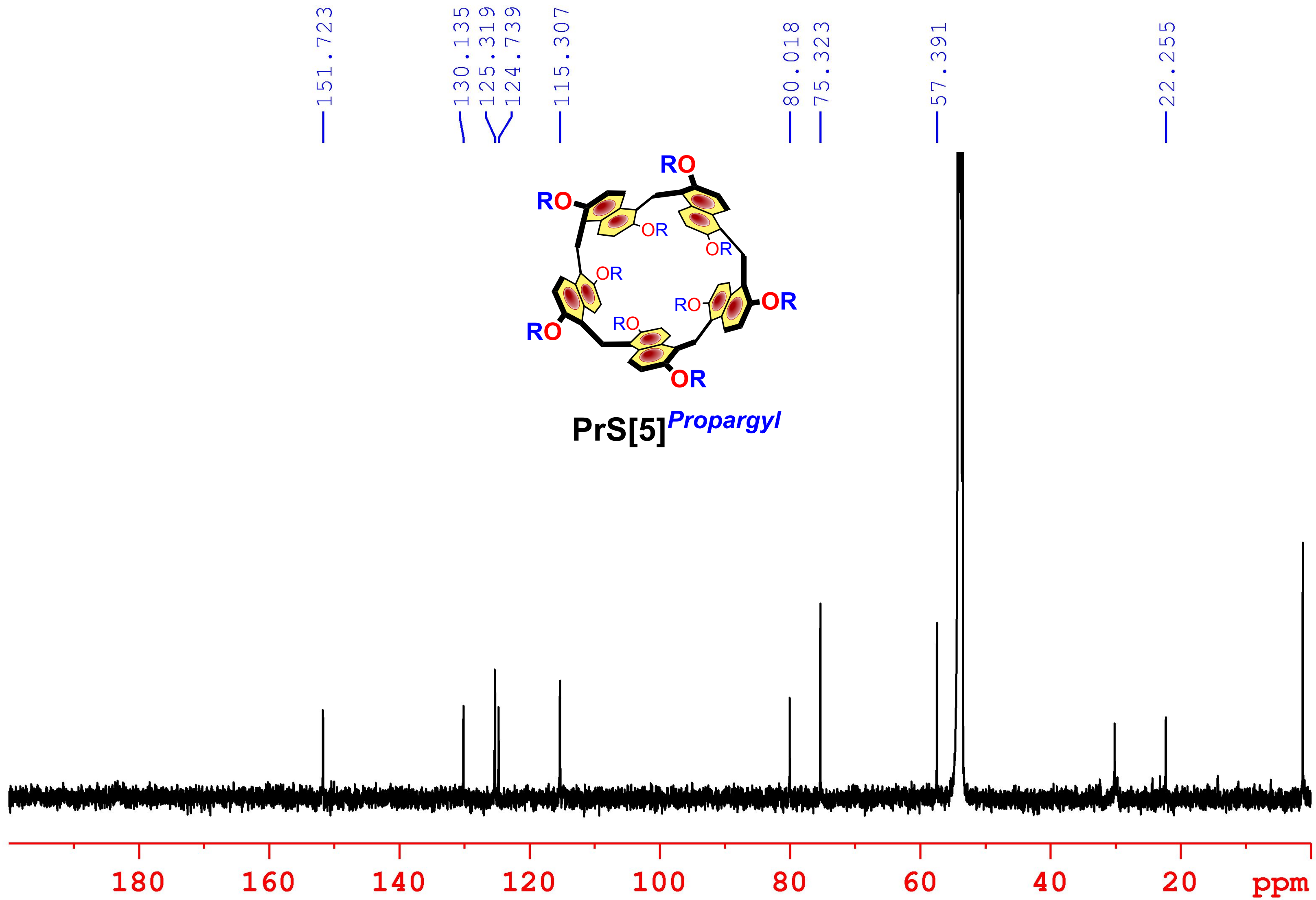

Figure S2: ^{13}C NMR spectrum of derivative **PrS[5]***Propargyl* (CD_2Cl_2, 150 MHz, 298 K).

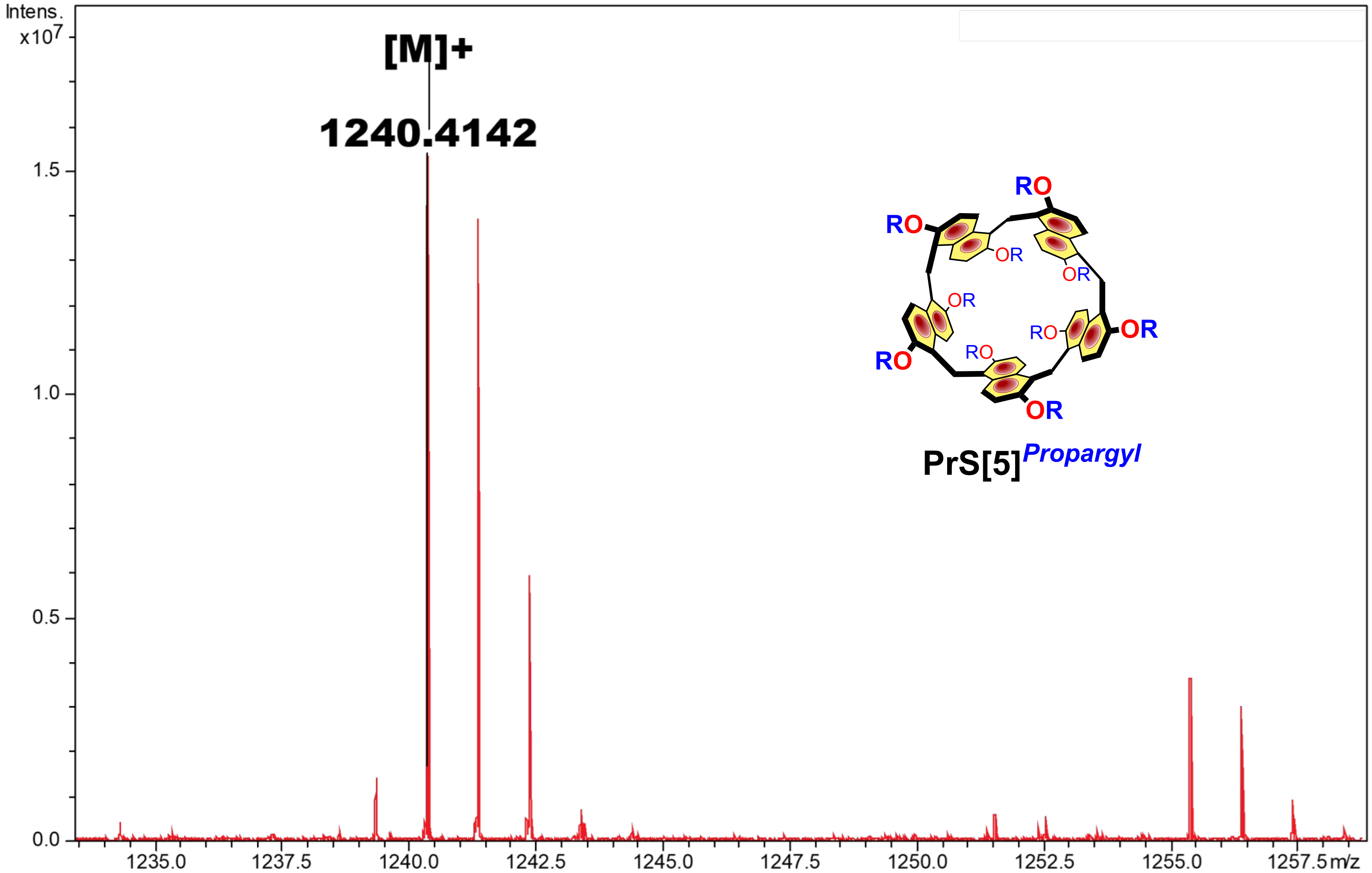

Figure S3: Significant portion of the HR MALDI FT-ICR mass spectrum of **PrS[5]*Propargyl*** [M]$^+$.

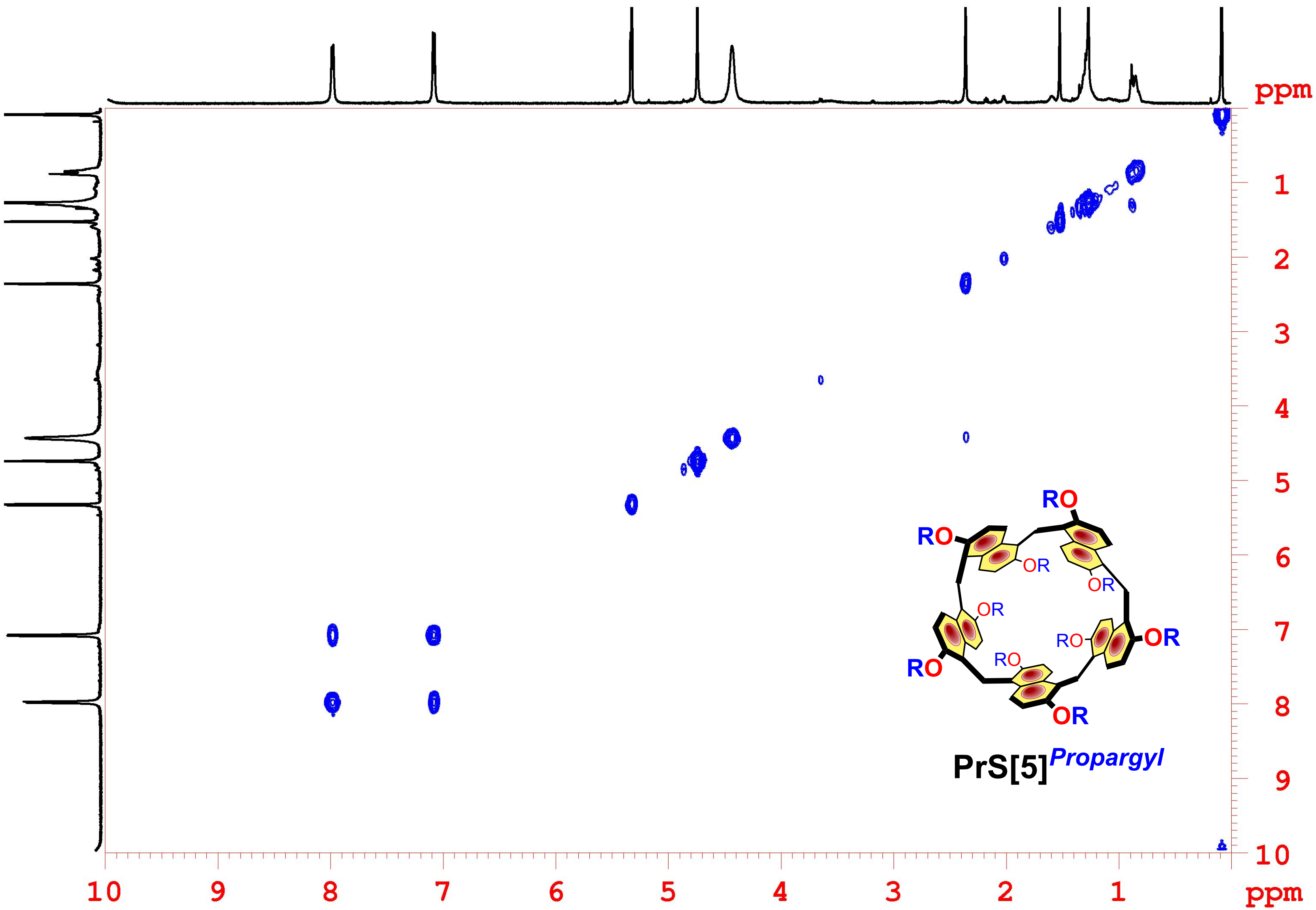

Figure S4: 2D-DQF COSY spectrum of derivative **PrS[5]**^{Propargyl} (CD_2Cl_2, 150 MHz, 298 K).

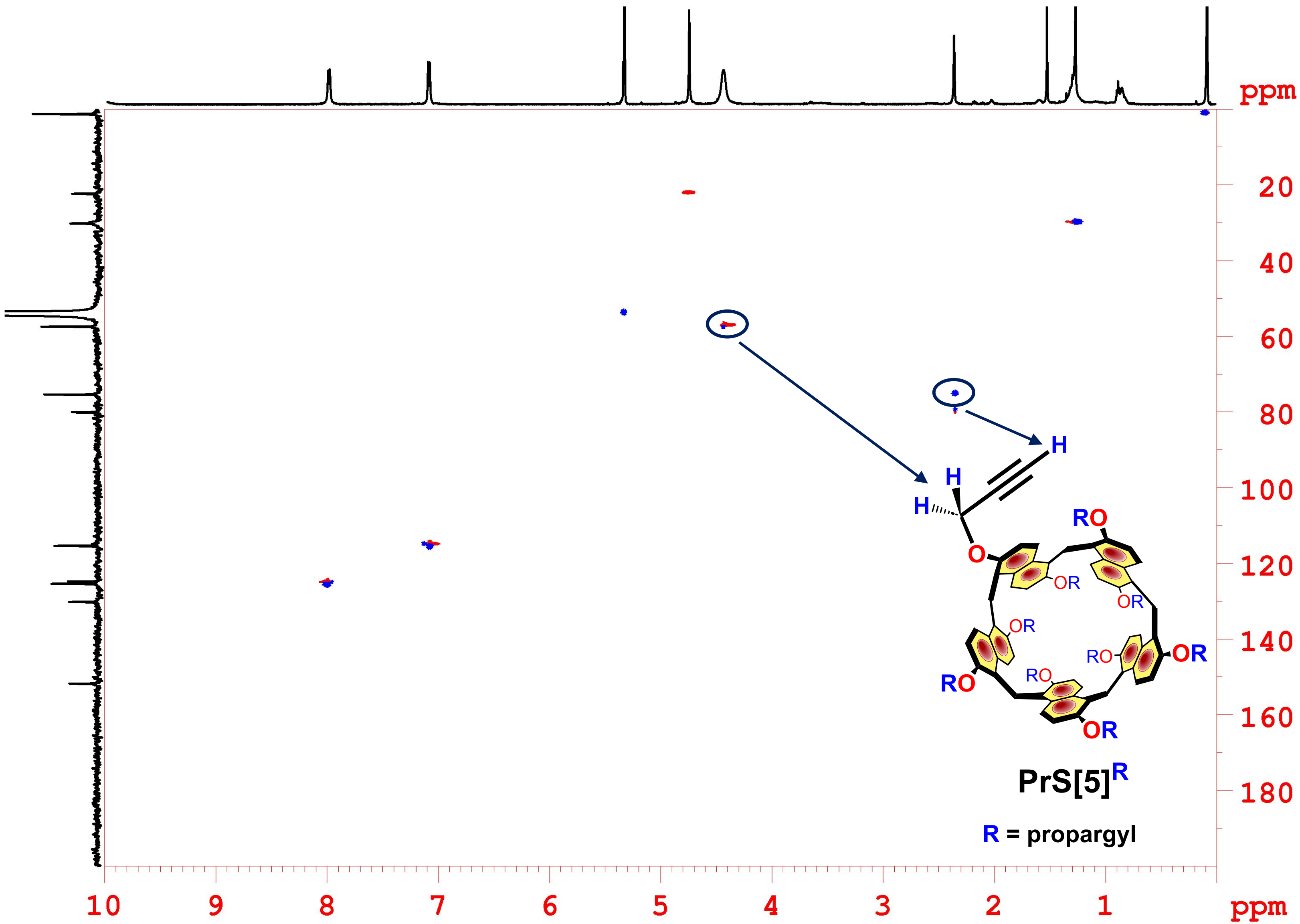

Figure S5: 2D-HSQC spectrum of derivative **PrS[5]**^{Propargyl} (CD_2Cl_2, 150 MHz, 298 K).

Copies of 1D and 2D NMR Spectra and HR Mass Spectra of PrS[5]^{PIP}

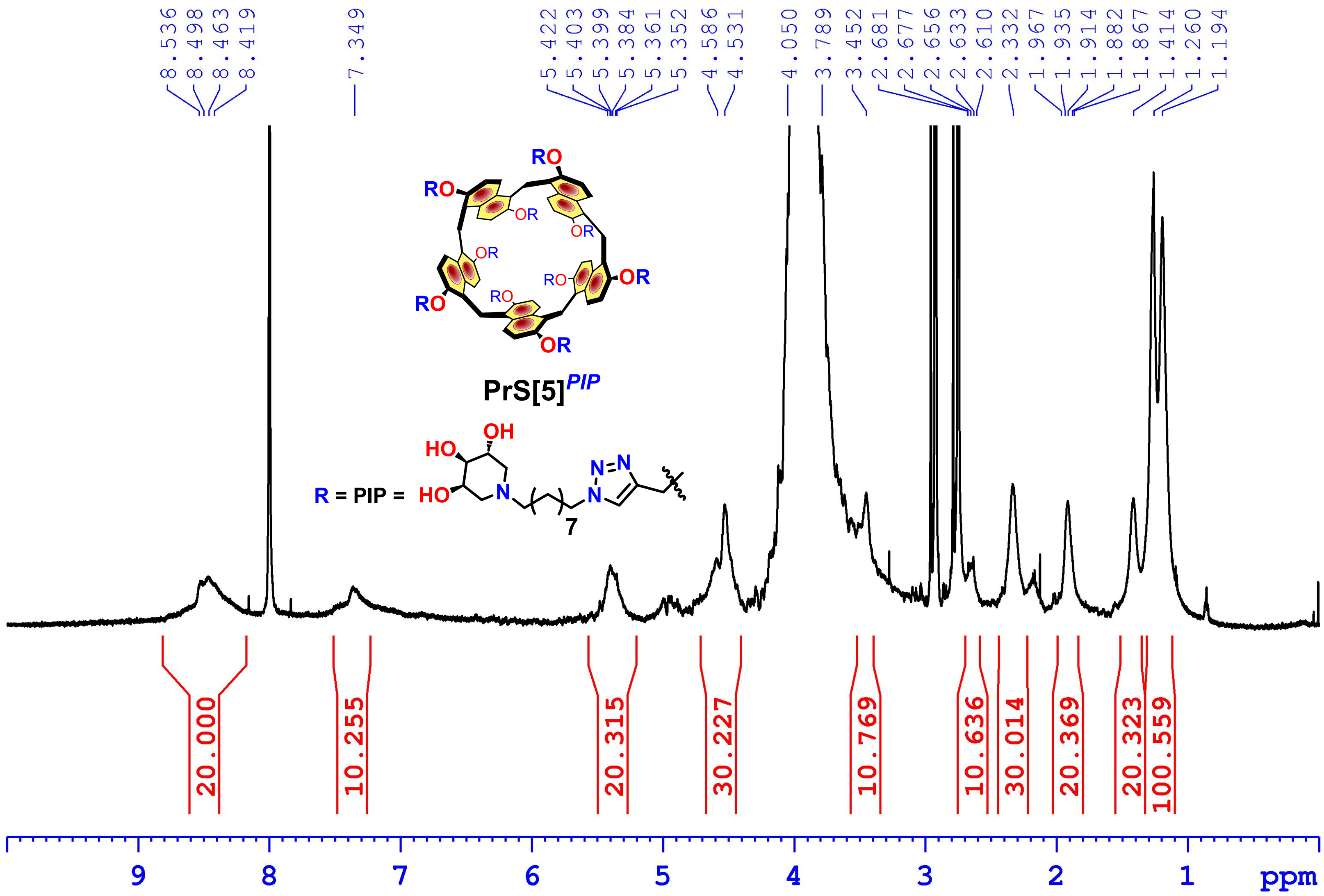

Figure S6: ^{1}H NMR spectrum of derivative **PrS[5]^{PIP}** (DMF-d_7, 600 MHz, 298 K).

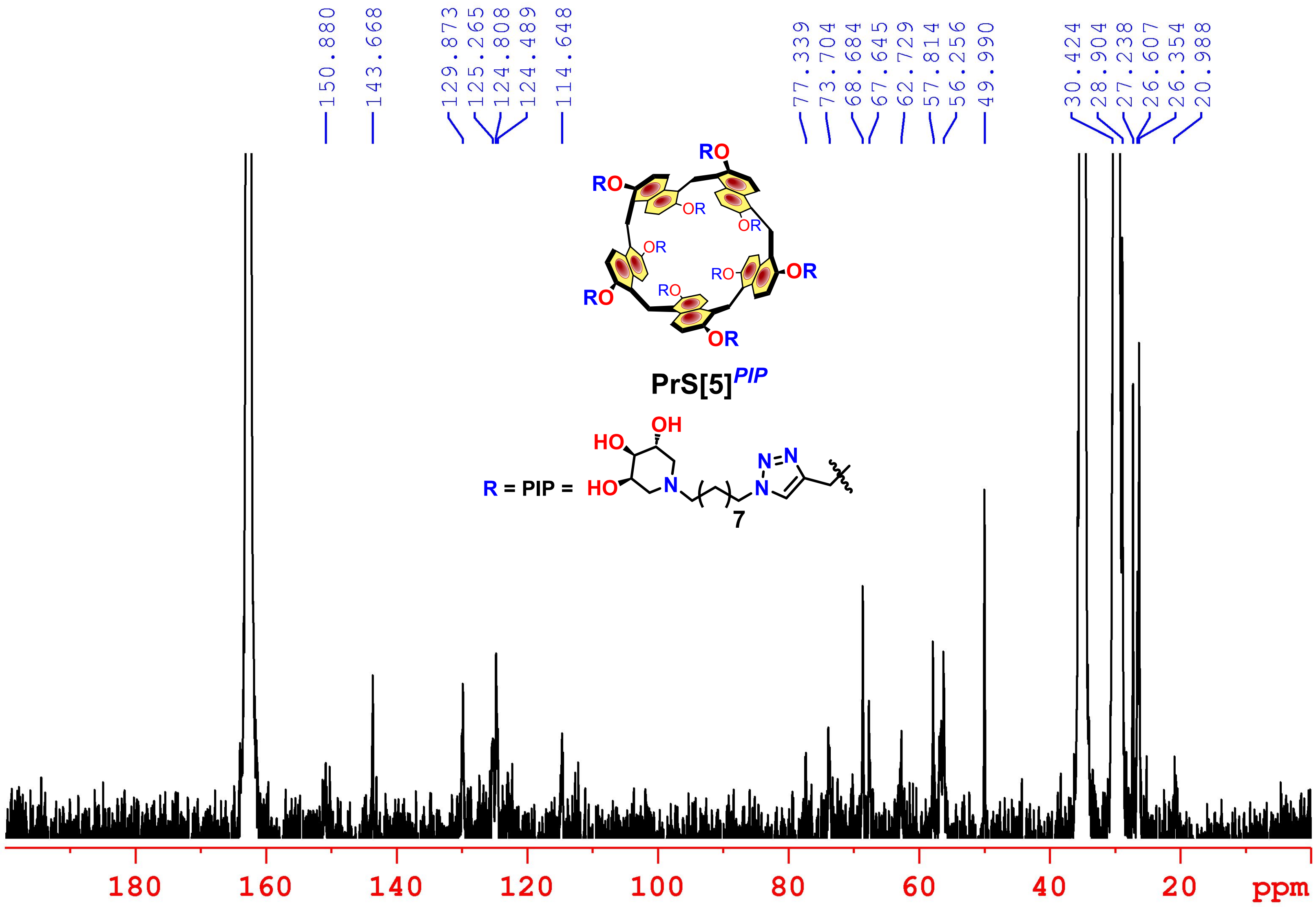

Figure S7: ^{13}C NMR spectrum of derivative **PrS[5]**^{PIP} (DMF-d_7, 150 MHz, 298 K).

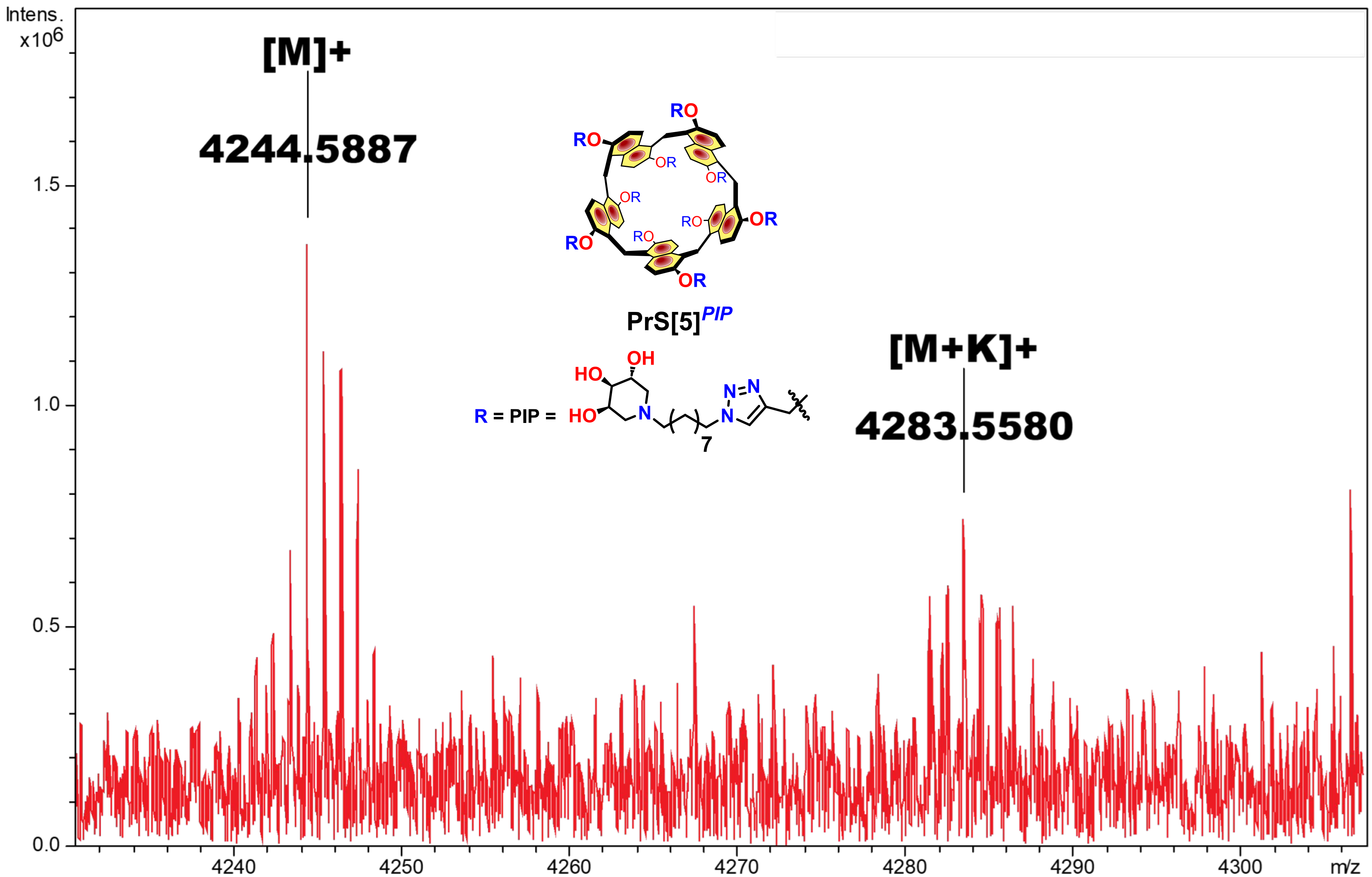

Figure S8: Significant portion of the HR MALDI FT-ICR mass spectrum of **PrS[5]^{PIP}** $[M]^+$ and $[M+K]^+$.

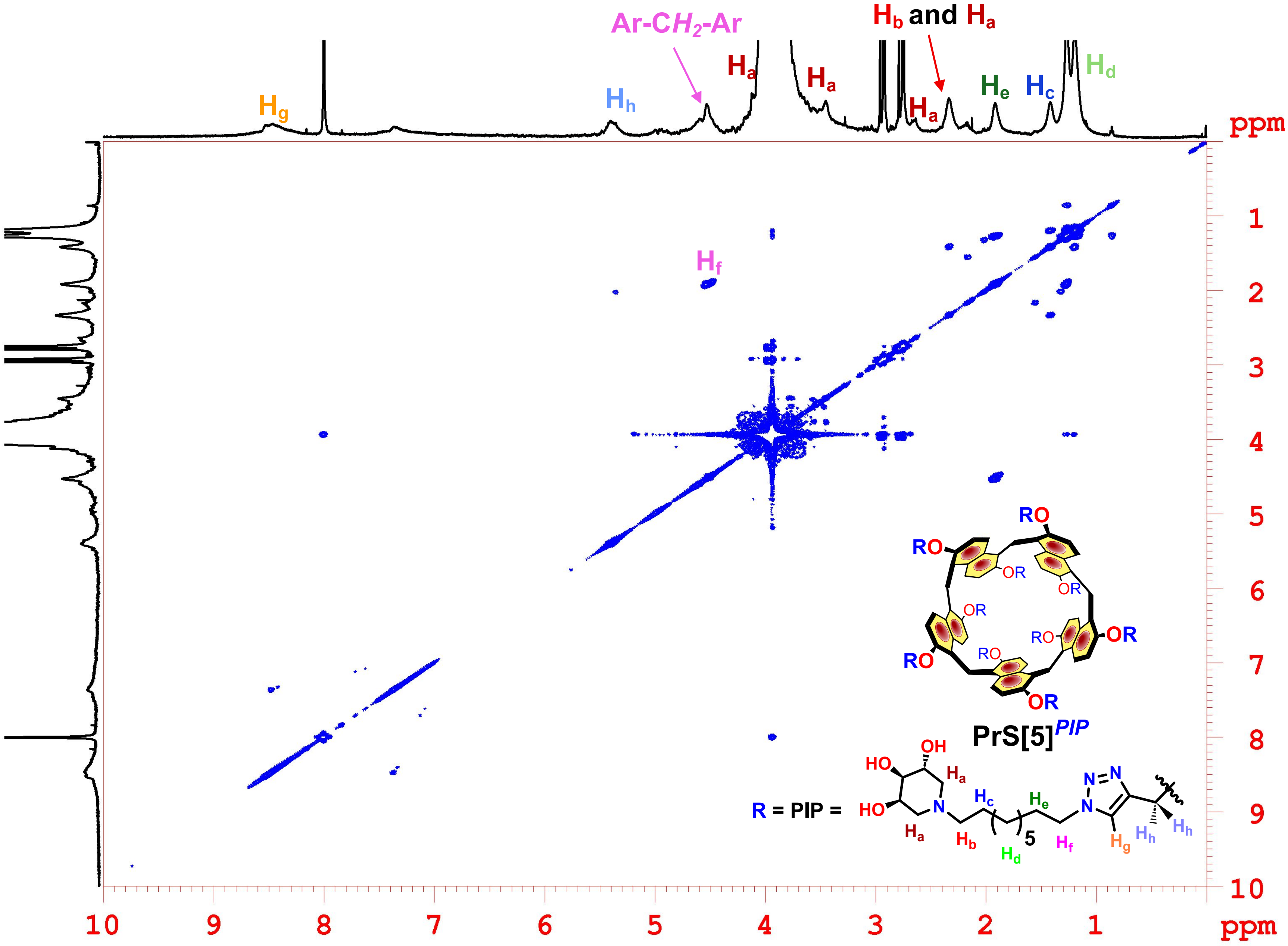

Figure S9: 2D-DQF COSY spectrum of derivative **PrS[5]**^{PIP} (DMF-d_7, 600 MHz, 298 K).

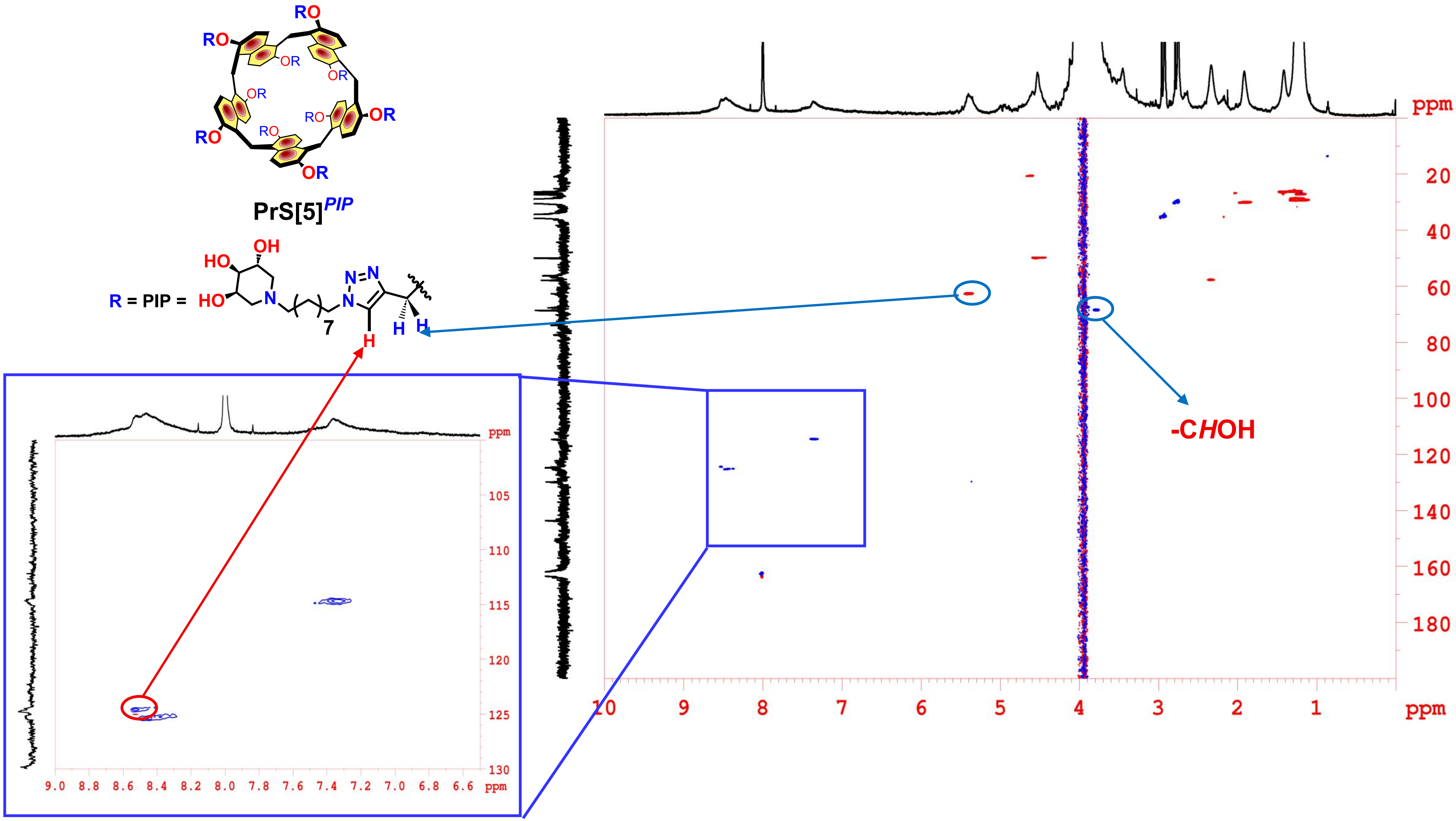

Figure S10: 2D-HSQC spectrum of derivative **PrS[5]**^{PIP} (DMF-d_7, 600 MHz, 298 K).

Copies of 1D and 2D NMR Spectra and HR Mass Spectra of PrS[5]^{PYR}

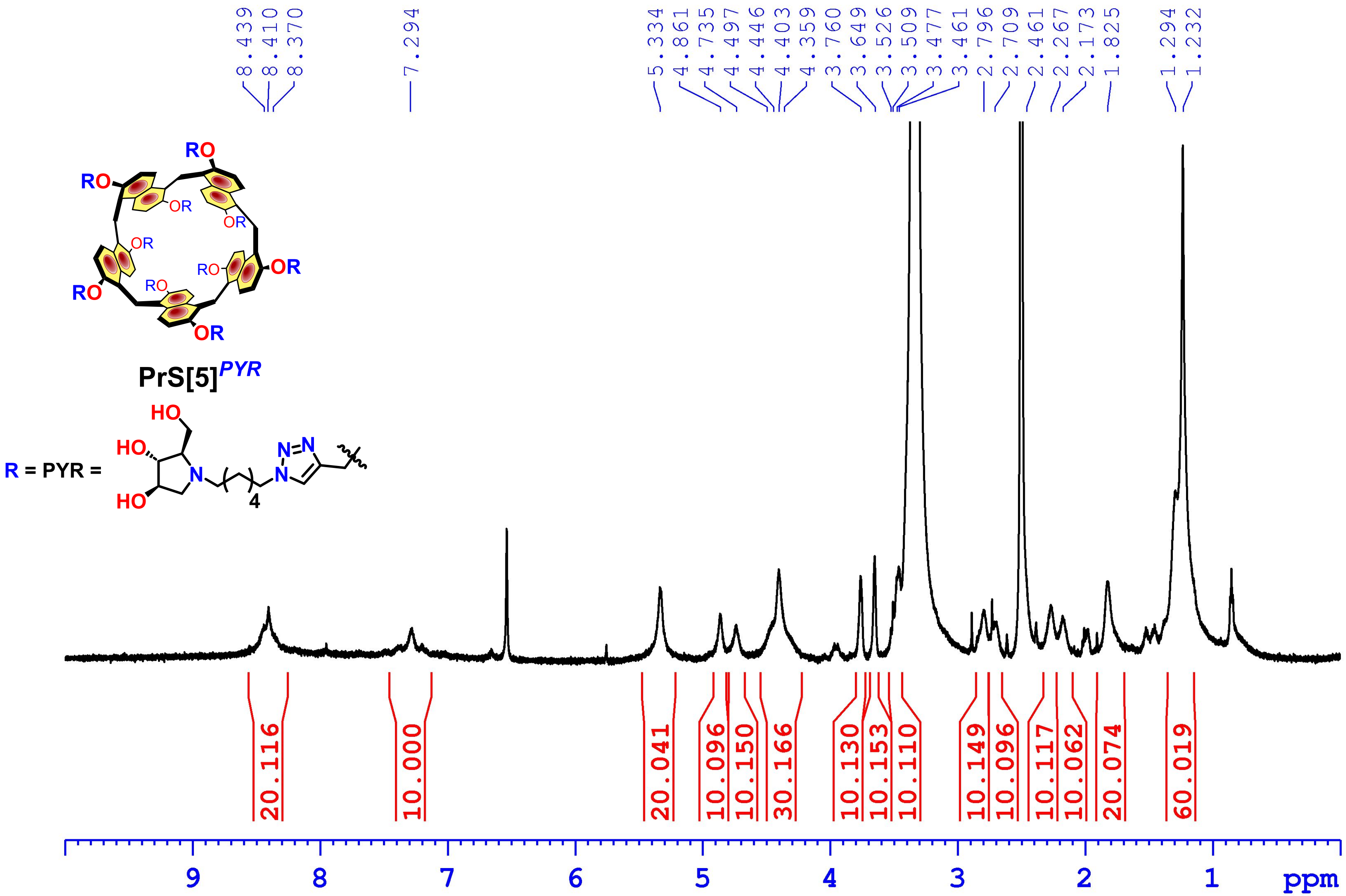

Figure S11: ^{1}H NMR spectrum of derivative **PrS[5]^{PYR}** (DMSO-d_6, 600 MHz, 298 K).

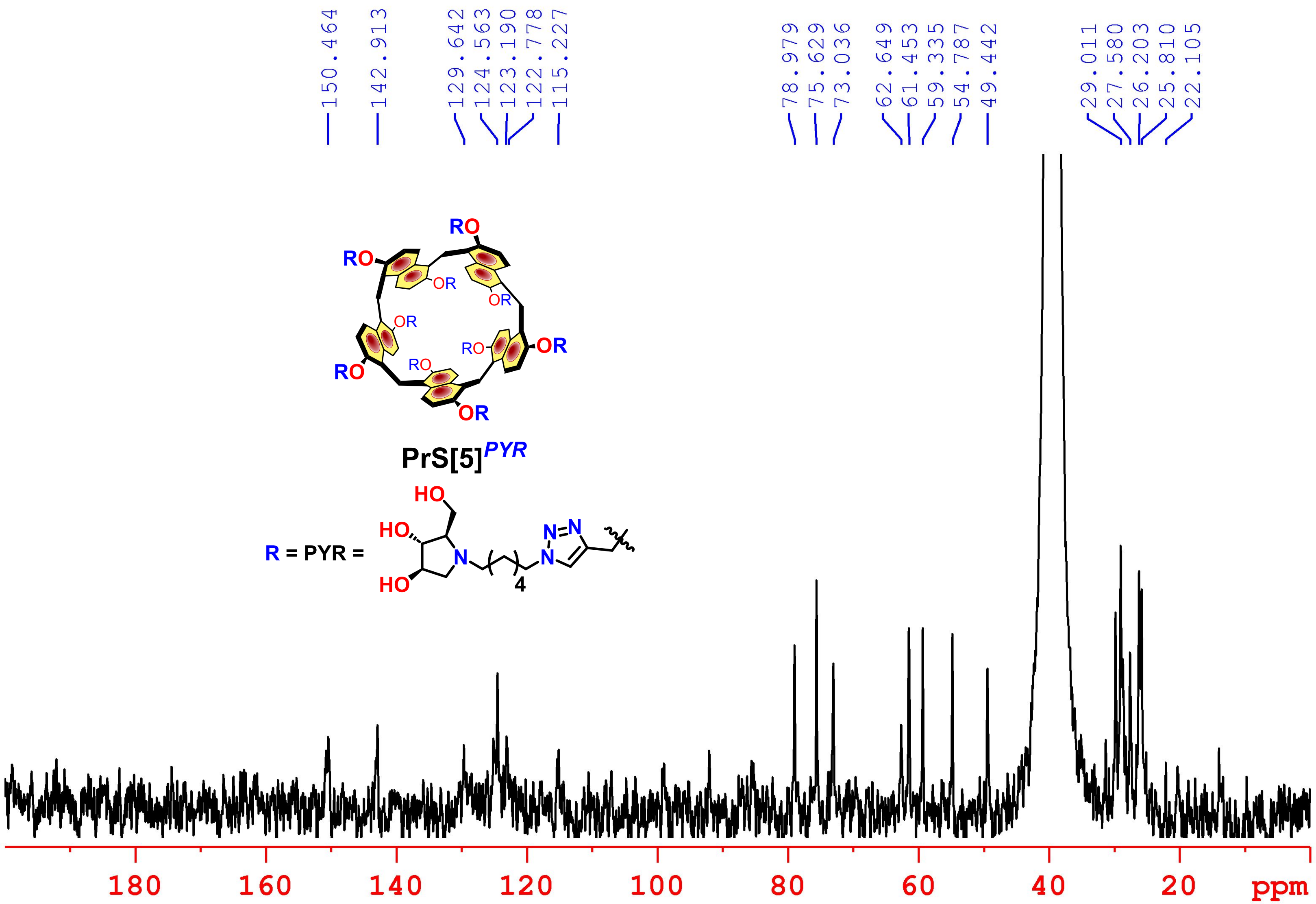

Figure S12: ^{13}C NMR spectrum of derivative **PrS[5]**^{PYR} (DMSO-d_6, 150 MHz, 298 K).

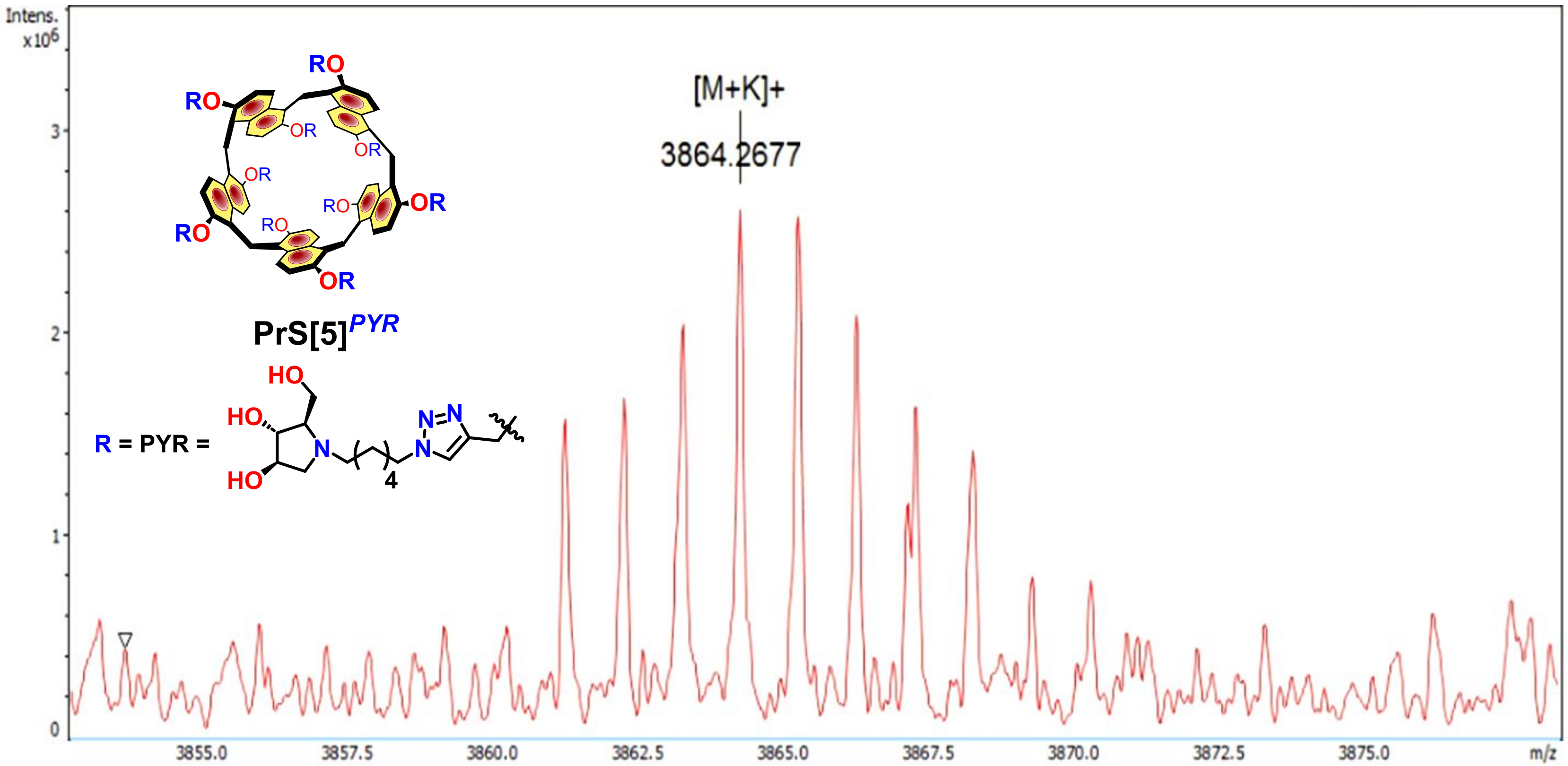

Figure S13: Significant portion of the HR MALDI FT-ICR mass spectrum of **PrS[5]**^{PYR} $[M+K]^+$.

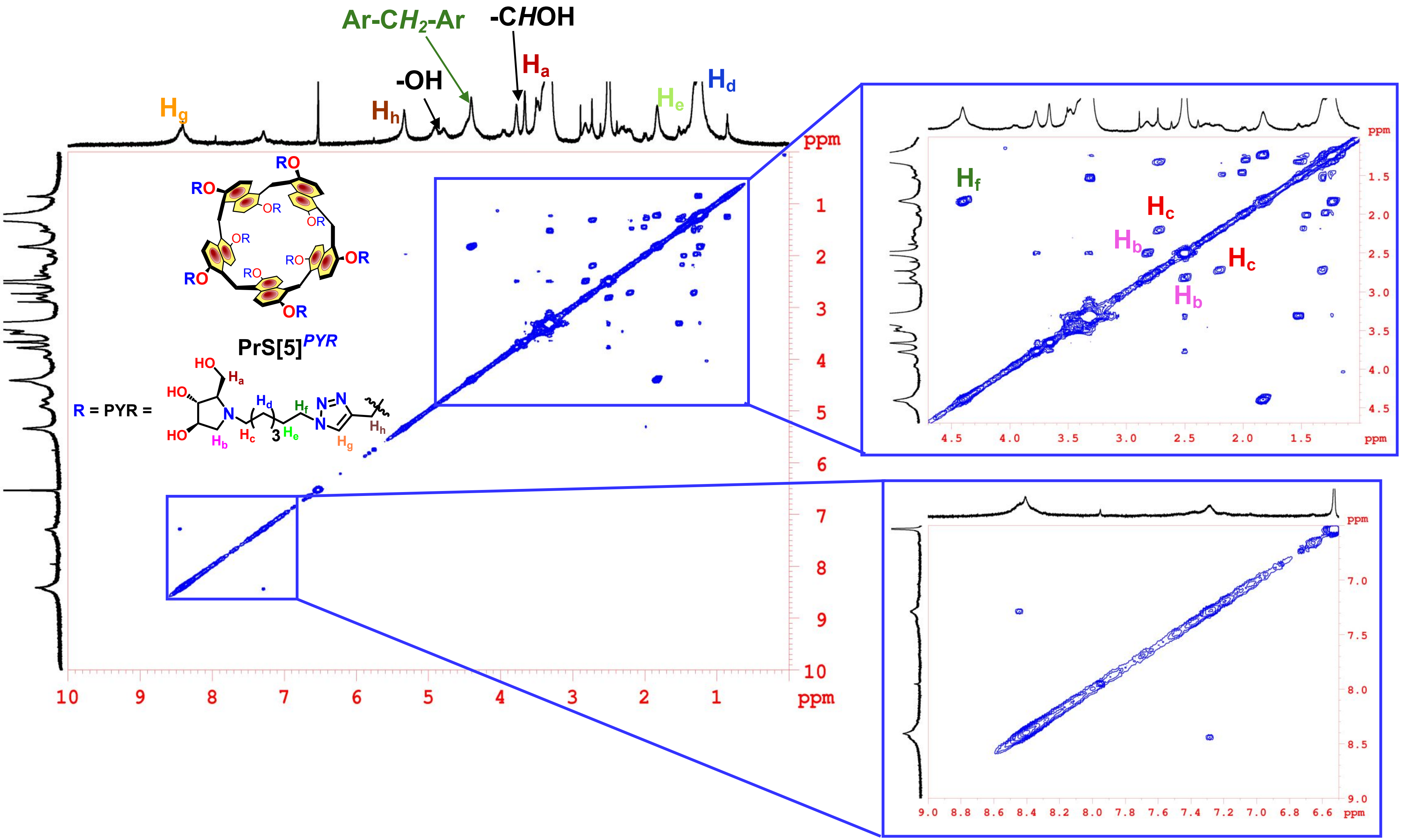

Figure S14: 2D-DQF COSY spectrum of derivative **PrS[5]^{PYR}** (DMSO-d_6, 600 MHz, 298 K).

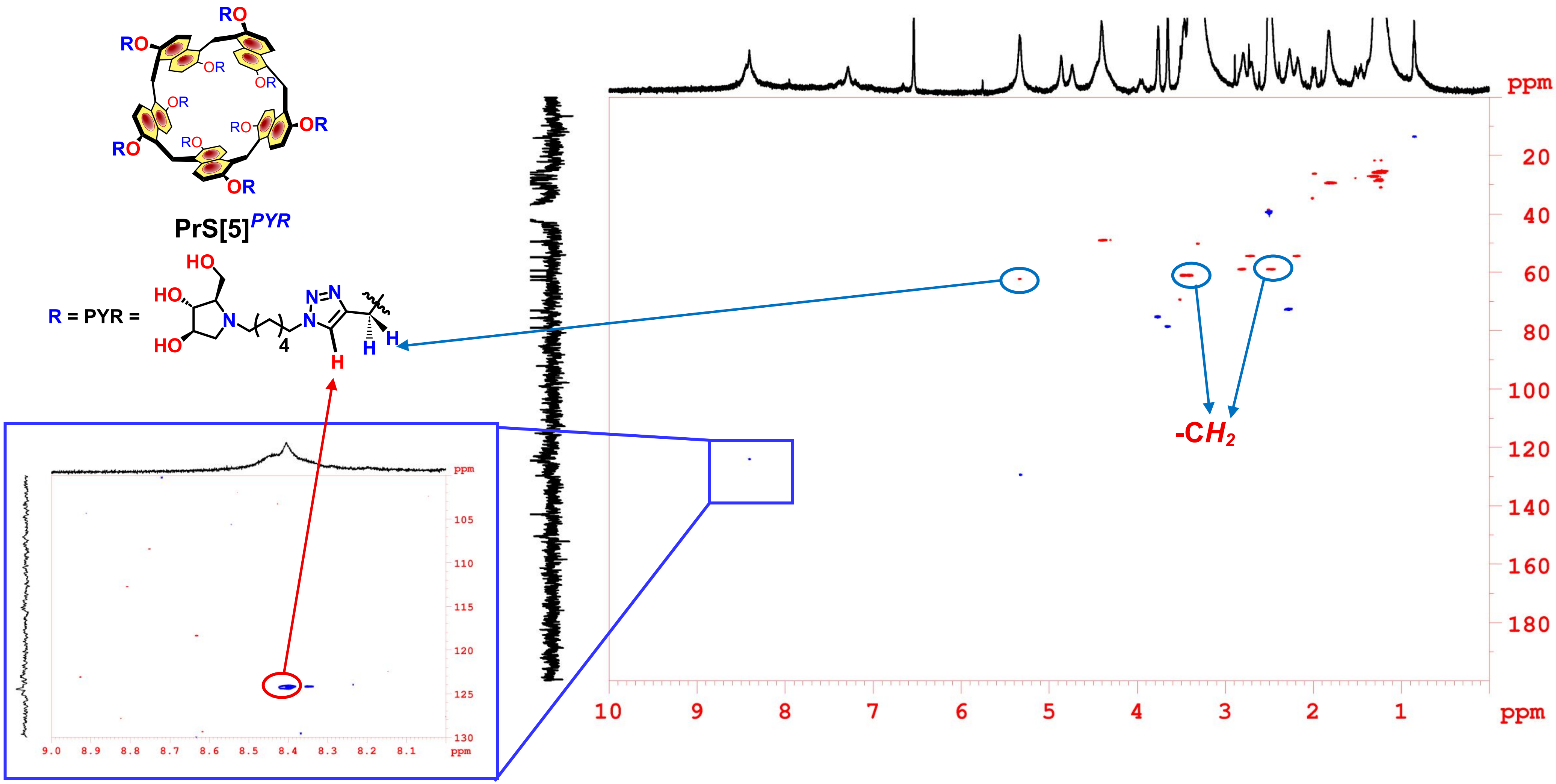

Figure S15: 2D-HSQC spectrum of derivative **PrS[5]**^{PYR} (DMSO-d_6, 600 MHz, 298 K).

Biochemical Characterization

Inhibitory activity towards human GCase from leukocyte homogenates.

All experiments on biological materials were performed in accordance with the ethical standards of the institutional research committee and with the 1964 Helsinki Declaration and its later amendments. In keeping with ethical guidelines, all blood and cell samples were obtained for storage and analysed only after written informed consent of the patients (and/or their family members) was obtained, using a form approved by the local Ethics Committee (Codice Protocollo: Lysolate “Late onset Lysosomal Storage Disorders (LSDs) in the differential diagnosis of neurodegenerative diseases: development of new diagnostic procedures and focus on potential pharmacological chaperones (PCs). Project ID code:16774_bio, 5 May 2020, Comitato Etico Regionale per la Sperimentazione Clinica della Regione Toscana, Area Vasta Centro, Florence, Italy). Control and patient samples were anonymized and used only for research purposes.

Compounds **PYROH, PrS[5]*PIP*** and **PrS[5]*PYR*** were screened towards GCase from leukocytes isolated from healthy donors (controls) at 0.5 mM concentration [7]. Isolated leukocytes were disrupted by sonication, and a micro BCA protein assay kit (Sigma-Aldrich) was used to determine the total protein amount for the enzymatic assay, according to the manufacturer’s instructions. Enzyme activity was measured in a flat-bottomed 96-well plate. Compounds solution (3 µL), 4.29 µg/µL leukocytes homogenate (7 µL) and substrate 4-methylumbelliferyl-β-D-glucopyranoside (3.33 mM, 20 µL, Sigma-Aldrich) in citrate/phosphate buffer (0.1 : 0.2, M/M, pH 5.8) containing sodium taurocholate (0.3%) and Triton X-100 (0.15%) at 37° C were incubated for 1 h. The reaction was stopped by the addition of sodium carbonate (200 µL; 0.5 M, pH 10.7) containing Triton X-100 (0.0025 %), and the fluorescence of 4-methylumbelliferone released by β-glucosidase activity was measured in SpectraMax M2 microplate reader (λ_{ex}=365 nm, λ_{em}=435 nm; Molecular Devices). Percentage GCase inhibition is given with respect to the control (without iminosugar). Data are mean±SD (n=3).

The reported procedure, used to determine GCase activity on fibroblast and leukocyte homogenates, is used and accredited for diagnostic purpose, performed at the Meyer Children Hospital, to confirm at biochemical level the diagnosis of Gaucher in suspicious patients [8] The reported procedure, used to determine GCase activity on fibroblast and leukocyte homogenates, is used and accredited for newborn screening performed by Meyer Children Hospital to identify the Gaucher patients. The experimental conditions established by C.P. Austin and colleagues allowed for the measurement of GCase activity at optimal levels, in particular thanks to the presence of sodium taurocholate (0.3%) and Triton X-100 (0.15%), which mimic its Saposin C cofactor[9].

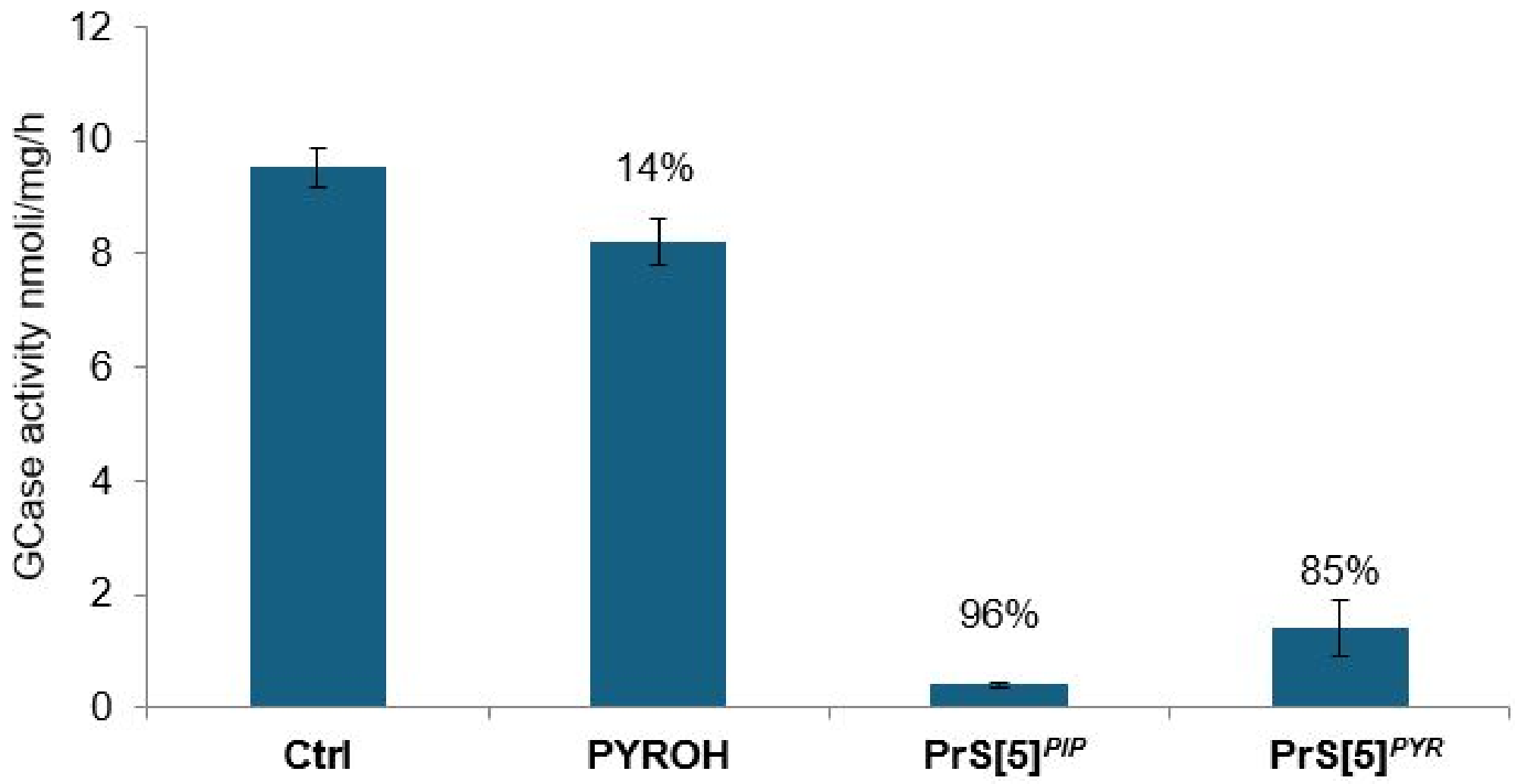

Figure S16: Activity of GCase derived from leukocyte homogenate in the presence of compounds **PYROH, PrS[5]*PIP*** and **PrS[5]*PYR*** at 0.5 mM. The corresponding calculated percentage of inhibition is indicated above each bar.

IC_{50} determination.

The IC_{50} values of compounds **PYROH, PrS[5]*PIP*** and **PrS[5]*PYR*** against GCase were determined by measuring the initial hydrolysis rate with 4-methylumbelliferyl-β-D-glucopyranoside (3.33 mM). Data obtained were fitted by using the appropriate Equation.

The IC_{50} values of inhibitors against GCase were determined by measuring the initial hydrolysis rate with 4-methylumbelliferyl-β-D- glucopyranoside (3.33 mM). Data obtained were fitted to the following equation using the Origin Microcal program.

$$\frac{Vi}{Vo} = \frac{Max - Min}{1 + \left(\frac{x}{IC_{50}}\right)^{slope}} + Min$$

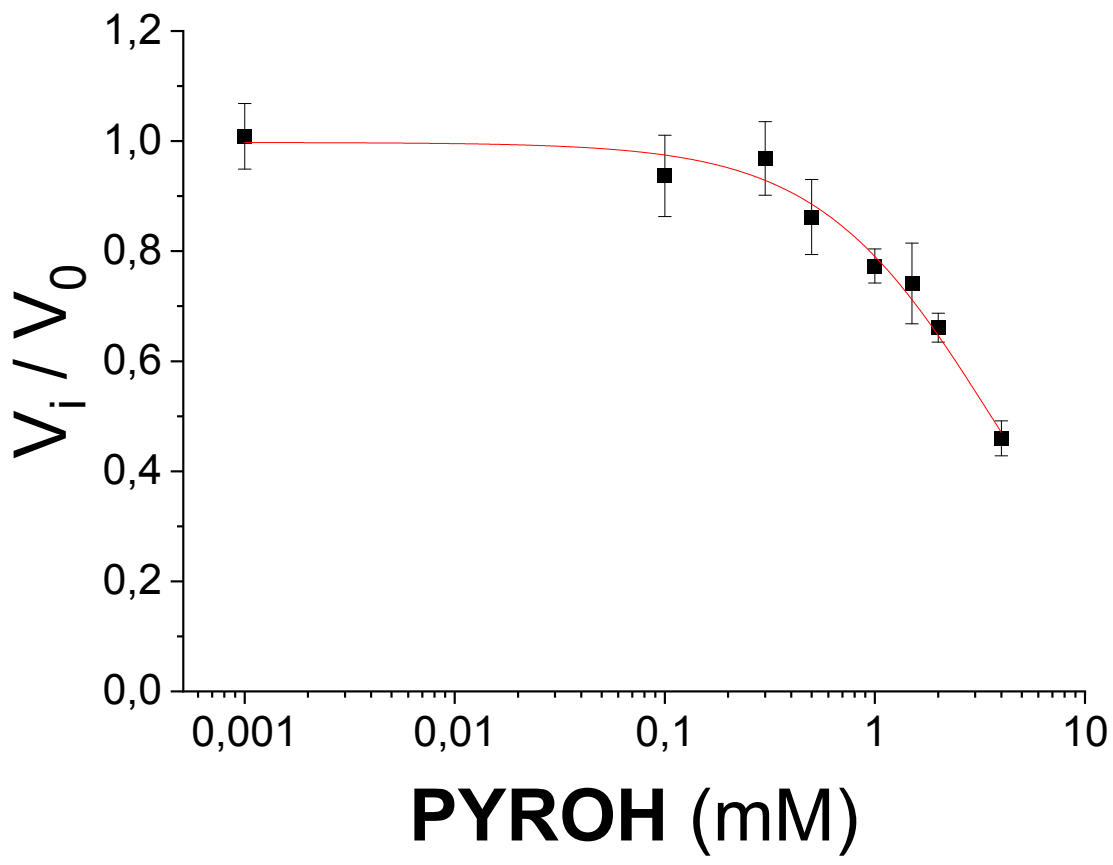

Figure S17: IC_{50} of compound **PYROH** value *vs* GCase derived from leukocyte homogenate= 3.6 ± 0.2 mM.

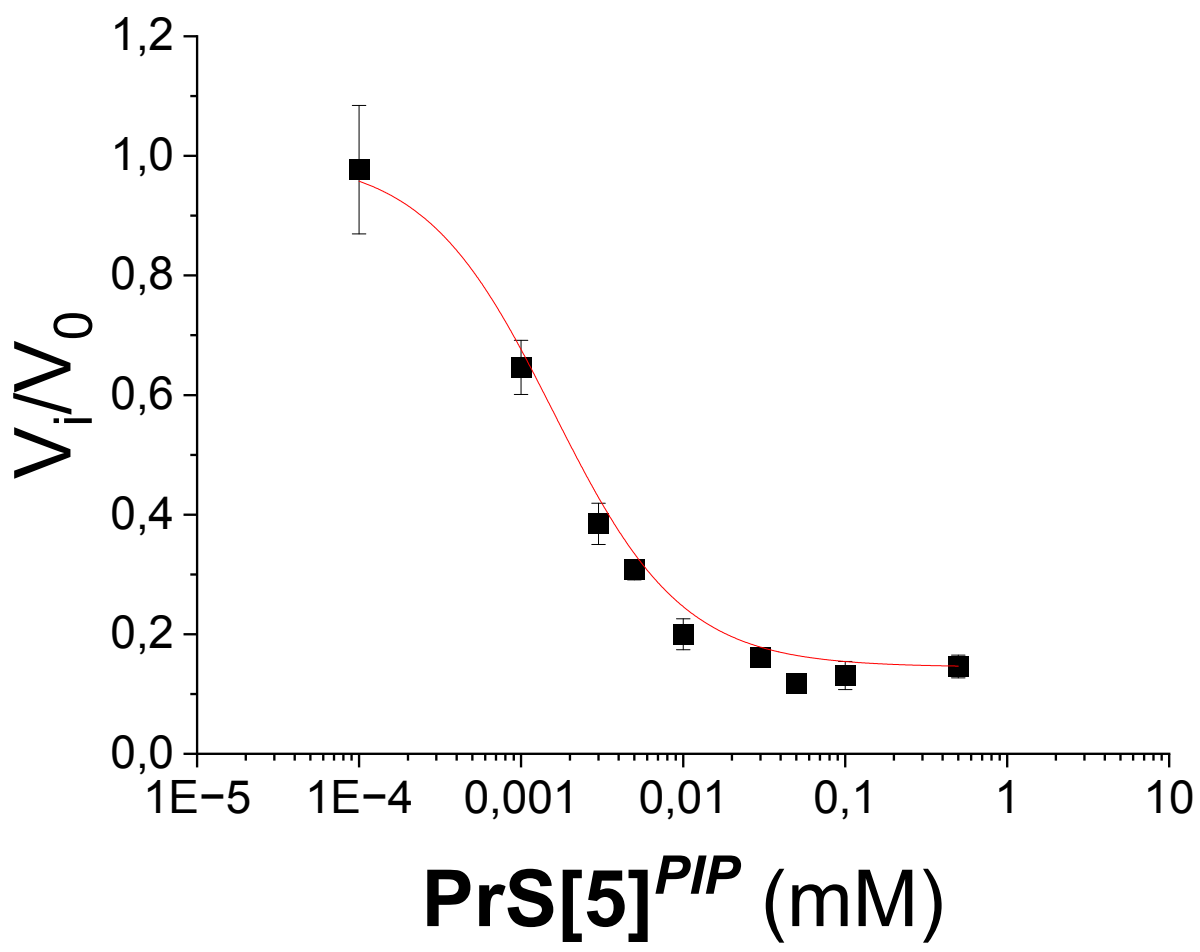

Figure S18: IC_{50} of compound **PrS[5]*PIP*** value *vs* GCase derived from leukocyte homogenate= 1.4 ± 0.2 μM.

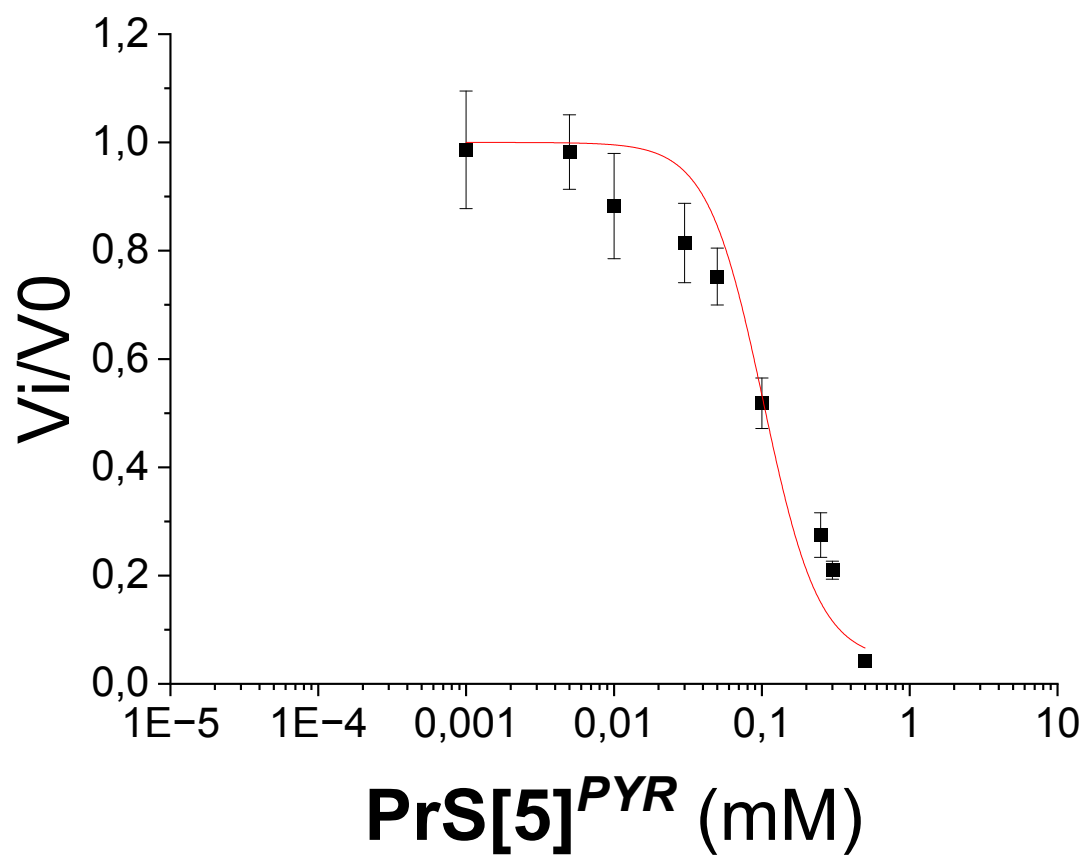

Figure S19: IC_{50} of compound **PrS[5]*PYR*** value *vs* GCase derived from leukocyte homogenate= 100 ± 20 μM.

Inhibitory activity towards human recombinant wild-type GCase.

The IC_{50} value of compounds **PIPOH**, **PYROH**, **PrS[5]**^{PIP} and **PrS[5]**^{PYR} against recombinant wild-type human GCase enzyme (VPRIV®, enzyme was diluted in bovine serum albumin (0.2%) at final concentration of 1.0 x 10^{-9} mg/mL) were determined by measuring the initial hydrolysis rate with 4-methylumbelliferyl-β-D-glucoside at different concentrations of compounds. A proper solution of compounds (3 µL), recombinant wild-type human GCase enzyme solution (7 µL) and substrate 4-methylumbelliferyl-β-D-glucoside (3.33 mM, 20 µL, Sigma–Aldrich) in citrate/phosphate buffer (0.1:0.2, M/M, pH 5.8) containing sodium taurocholate (0.3%) and Triton X-100 (0.15%) at 37 °C were incubated for 1 h. The reaction was stopped by addition of sodium carbonate (200 µL; 0.5M, pH 10.7) containing Triton X-100 (0.0025 %), and the fluorescence of 4-methylumbelliferone released by β-glucosidase activity was measured in SpectraMax M2 microplate reader (λ_{ex}=365 nm, λ_{em}=435 nm; Molecular Devices). Percentage GCase inhibition is given with respect to the control (without iminosugar). Data are mean ± SD (n=3). Data obtained were fitted accordingly to the above-mentioned equation and using the Origin Microcal program.

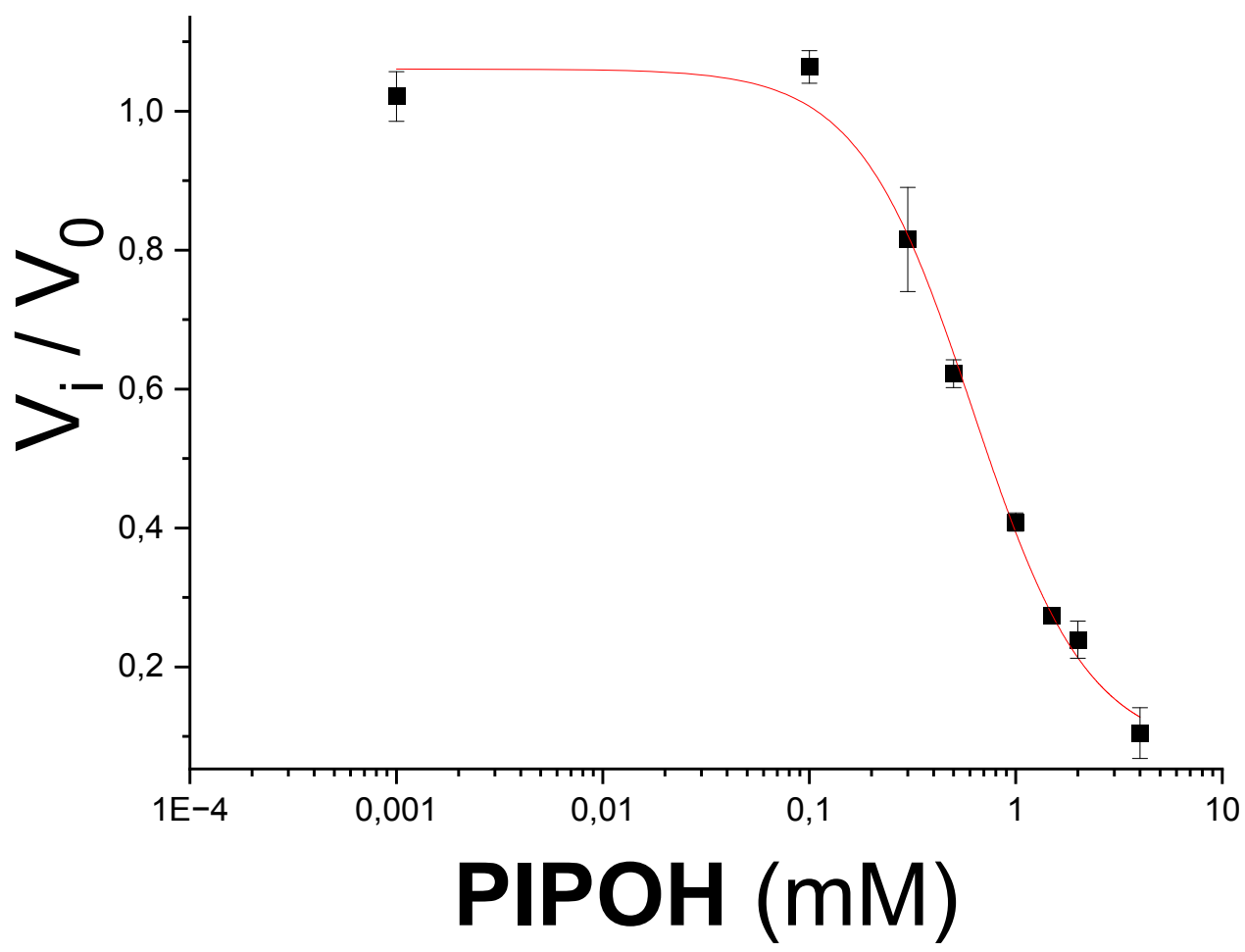

Figure S20: IC_{50} of compound **PIPOH** value *vs* recombinant GCase (*rh*-GCase)= 0.60 ± 0.06 mM.

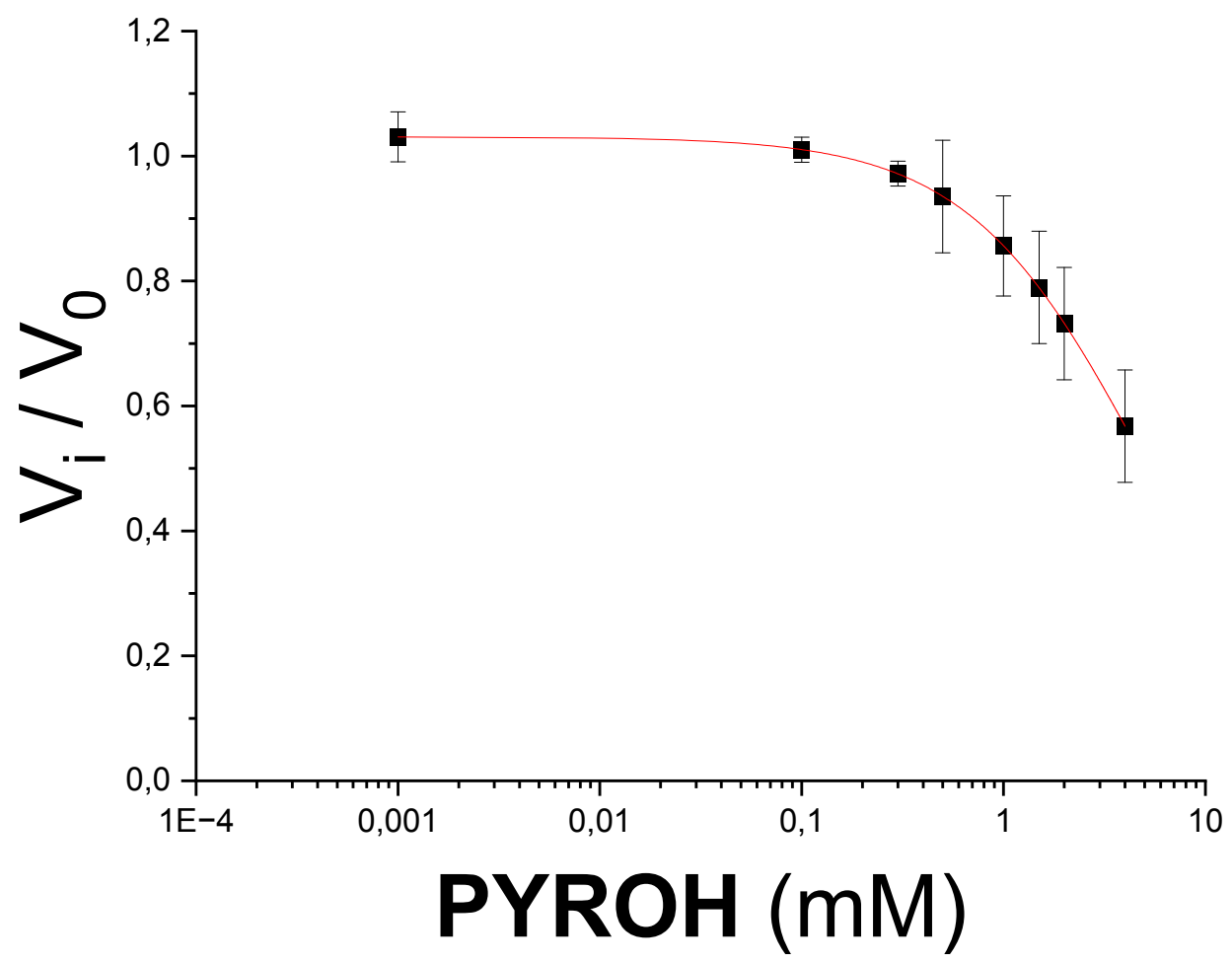

Figure S21: IC_{50} of compound **PYROH** value *vs* recombinant GCase (*rh*-GCase)= 4.9 ± 0.5 mM.

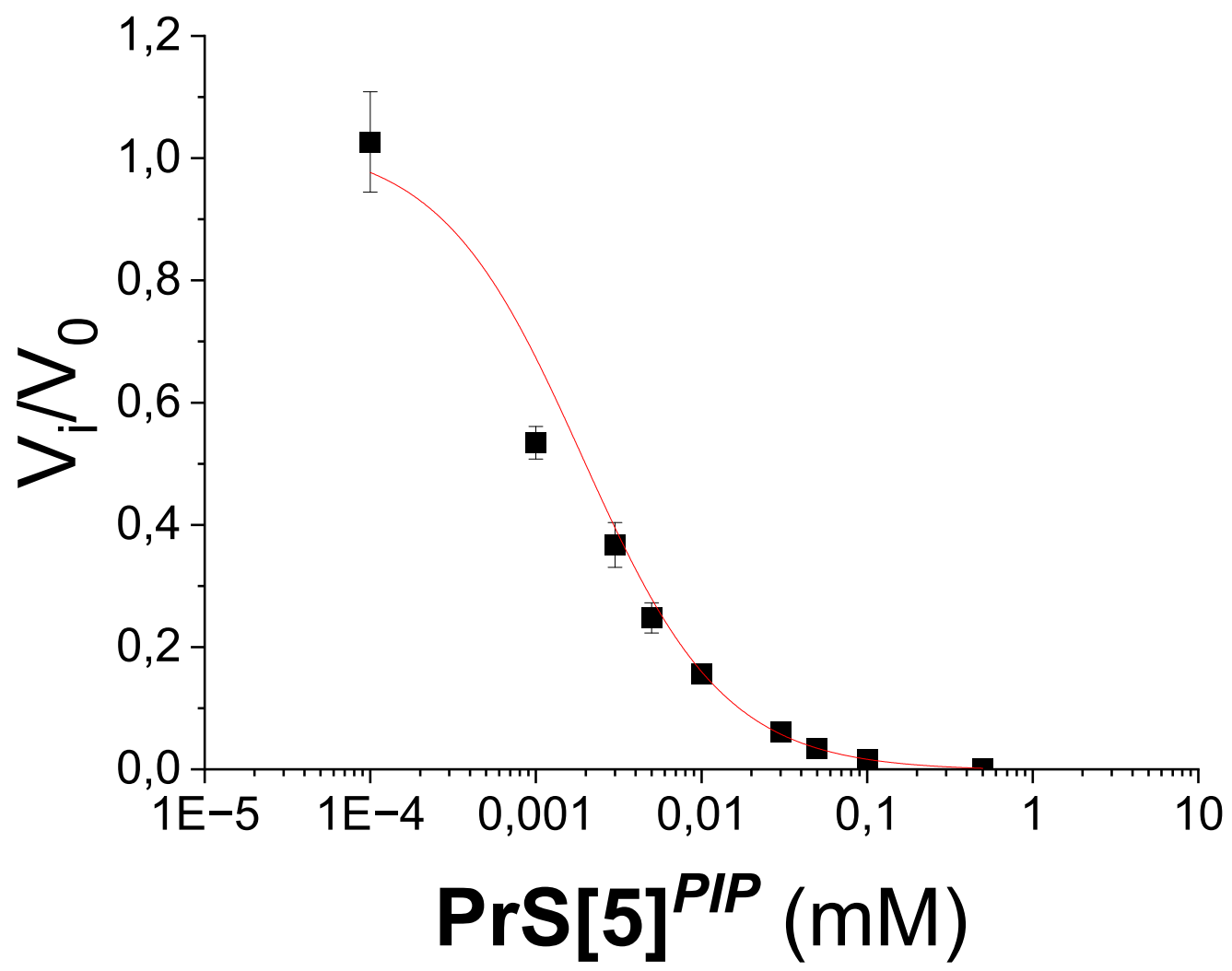

Figure S22: IC_{50} of compound **PrS[5]*PIP*** value *vs* recombinant GCase (*rh*-GCase)= 2.0 ± 0.1 μM.

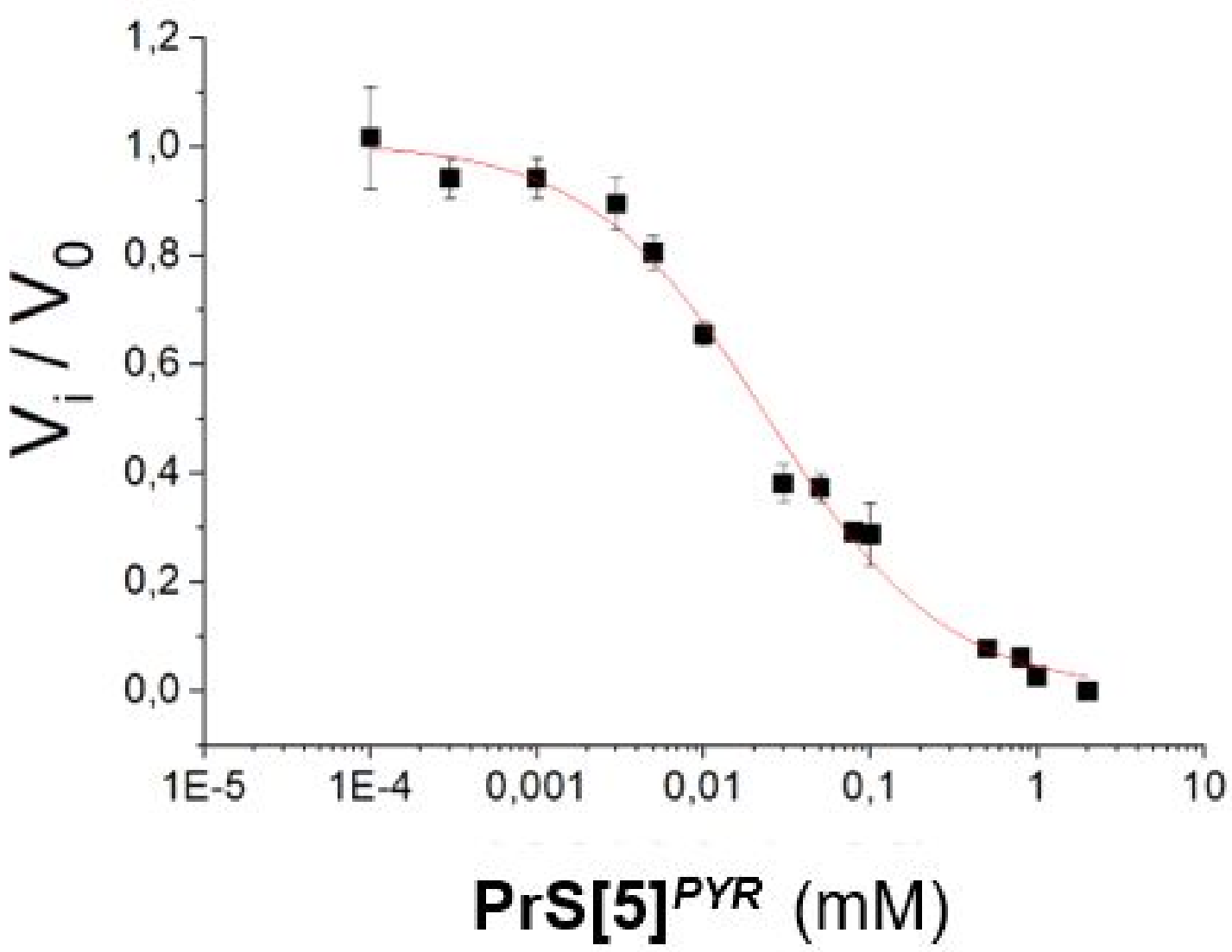

Figure S23: IC_{50} of compound **PrS[5]*PYR*** value *vs* recombinant GCase (*rh*-GCase)= 30 ± 3μM.

Assessment of the Prismarene Scaffold as a GCase Inhibitor

To assess the role of the scaffold itself to GCase inhibition, derivatives **PrS[5]^{H}** and its methyl analogue **PrS[5]^{Me}** were evaluated at 0.1 and 1 mM concentrations both towards human wild-type GCase from leukocyte homogenates and recombinant GCase (VPRIV®), following the protocols described above.

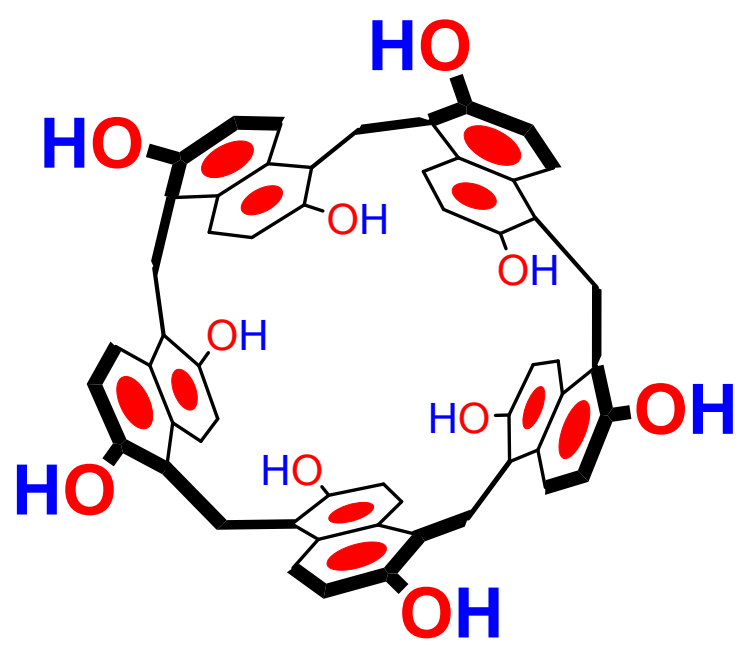

PrS[5]^{H}

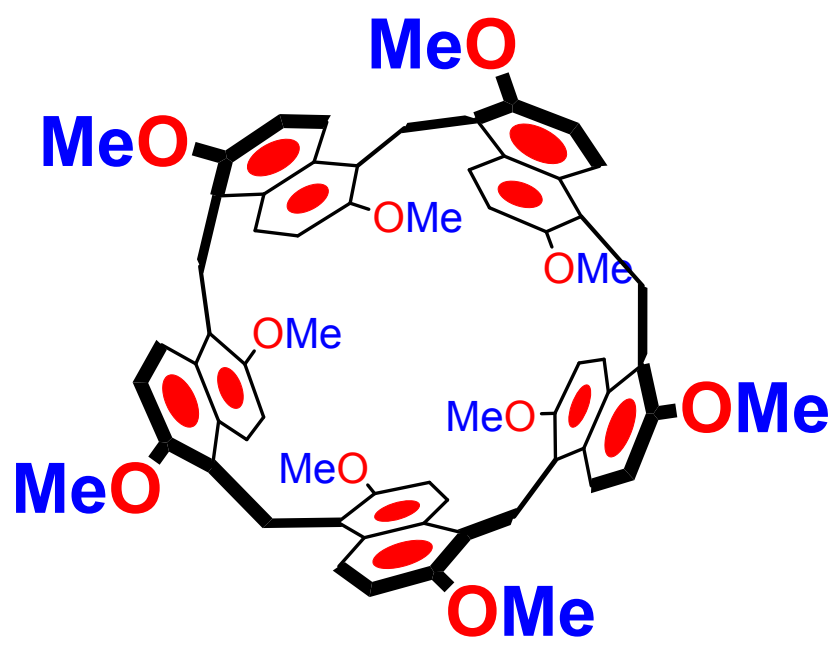

PrS[5]^{Me}

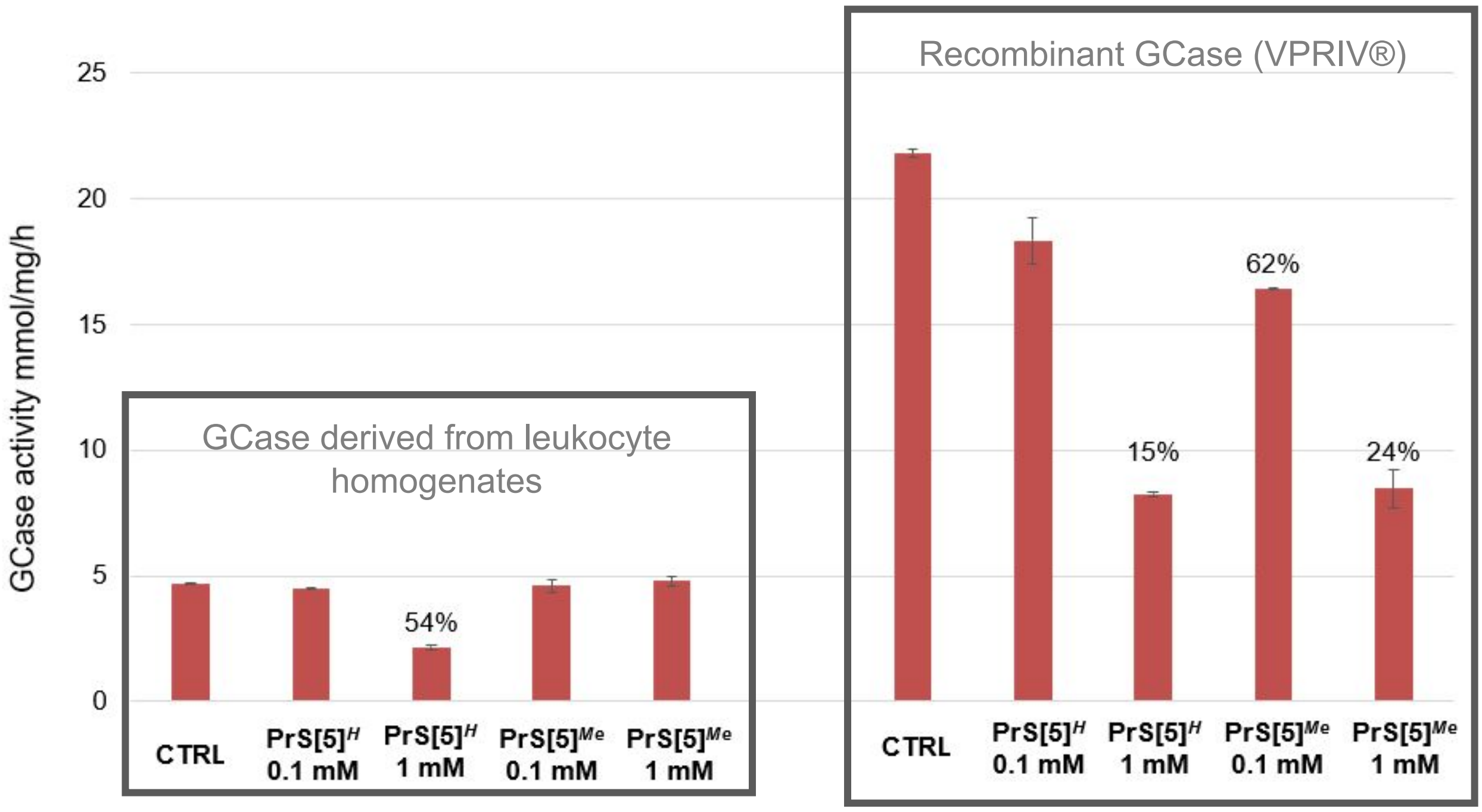

Figure S24: Activity of GCase derived from leukocyte homogenates (left) and recombinant GCase (VPRIV®) (right) in the presence of compounds **PrS[5]^{H}** and **PrS[5]^{Me}** at 0.1 and 1 mM concentrations. The corresponding calculated percentage of inhibition is indicated above each bar.

Chaperoning activity assays

Gaucher disease patients' cells were obtained from the "Cell line and DNA Biobank from patients affected by Genetic Diseases" (Gaslini Hospital, Genova, Italy)-Telethon Genetic Biobank Network). The cell passage number of fibroblasts is between 12 to 28.

Intact cell enzyme assay[10]

To determine the GCase activity in intact cells, 3000-4000 cells were plated into 96-well black plate with clear bottom in DMEM supplemented with fetal bovine serum (10%), penicillin/streptomycin (1%), and glutamine (1%) and incubated at 37°C with 5% CO_2 for 24 h. The medium was removed, and fresh medium containing the acetals or IFG or ABX (as a control) was added to the cells and left for 4 days. The enzyme activity assay was performed after removing the medium supplemented with compounds. The monolayers were washed with Dulbecco's PBS solution (Gibco pH 7.4). PBS (16 µL) and acetate buffer (0.2M, 16 µL, pH 4.0) were added to each well. The reaction was started by the addition of 4-methylumbelliferyl β-D-glucopyranoside (4 mM, 25 µL, Sigma) to each well, followed by incubation at 37 °C for 2 h. The reaction was stopped by lysing the cells with glycine buffer (0.2M, 200 µL, pH 10.8). The fluorescence was also measured by the release of 4-methylumbelliferone with an excitation wavelength of 355 nm and an emission wavelength of 460 nm (SpectraMax M5, Molecular Devices). The fluorescence of untreated cells was compared with those treated with compounds. The GCase enzyme activity data in CTRL fibroblasts were expressed as mean ± standard deviation (n=8), while in treated fibroblasts the data were expressed as mean ± standard deviation (n=5). Under these experimental conditions, the activity of non-lysosomal β-glucosidase was shown to be negligible (typically around 5%) at pH 4. This was confirmed by J. W. Kelly and co-workers using chonduritol B-epoxide (CBE), an irreversible inhibitor that specifically targets lysosomal GCase but does not affect non-lysosomal β-glucosidase. As expected, the CBE-insensitive activity was minimal, indicating that the observed GCase activity largely originated from the lysosomal enzyme[11]. Statistical Analysis: Statistical significance was evaluated using Student's t-test. A p-value lower than 0.05 was considered to be statistically significant. The single (*), double (**), triple (***) and quadruple (****) asterisks refer to p-values lower than 0.05, 0.01, 0.001 and 0.0001, respectively. Statistical analysis was performed using the GraphPad Prism 8.

a)

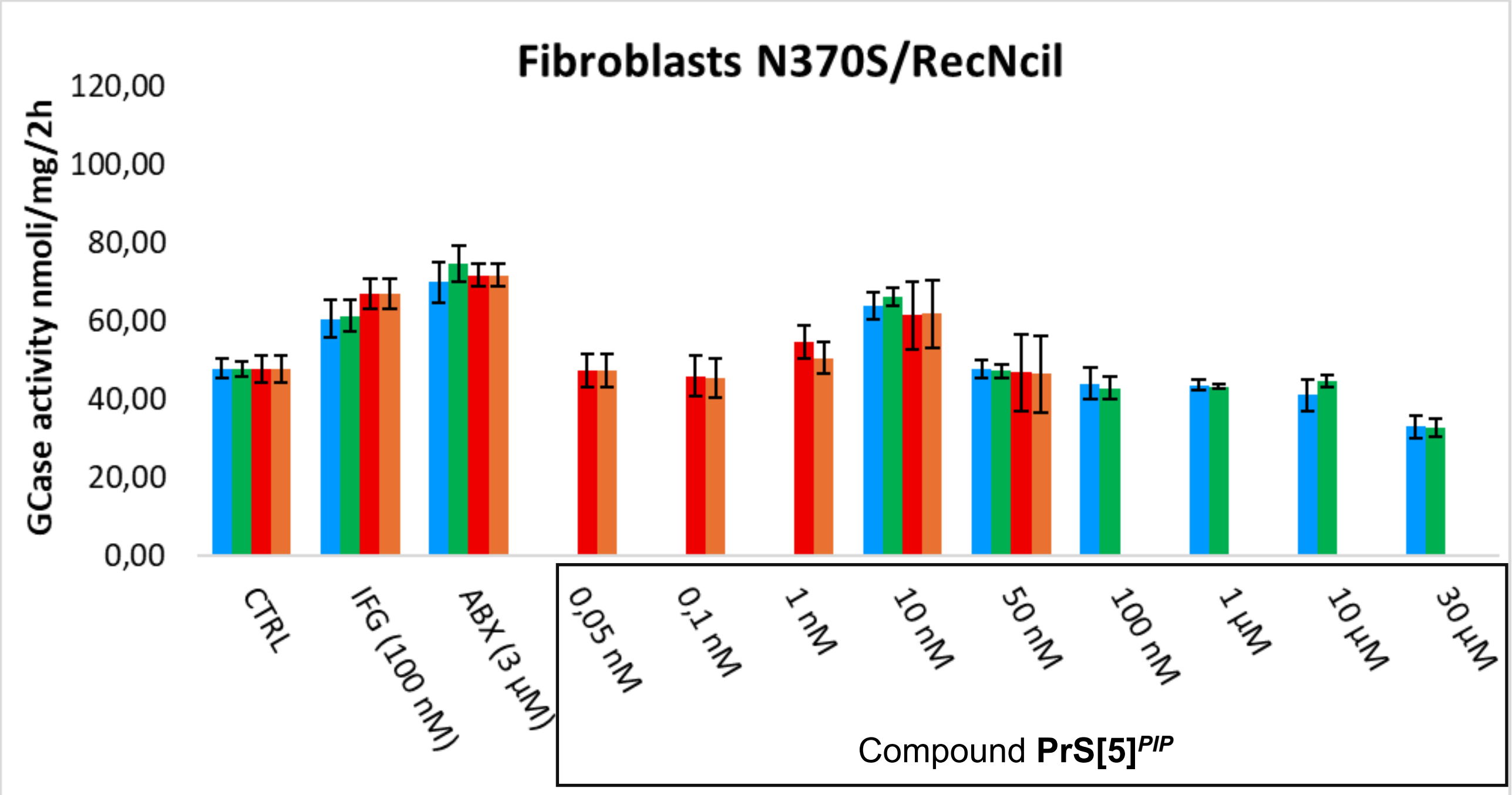

b)

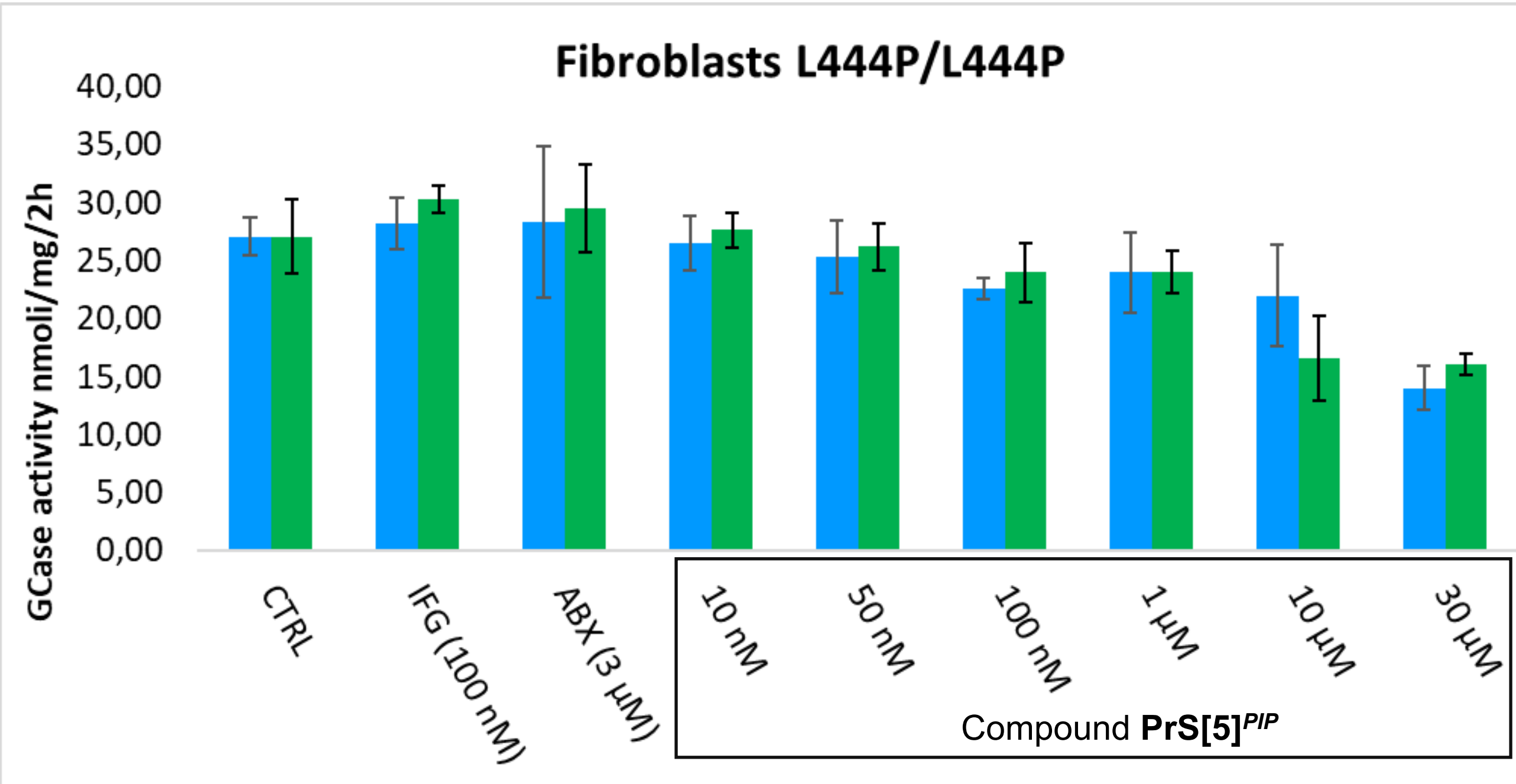

Figure S25: Fibroblasts derived from GD patients bearing a) N370S/RecNcil and b) L444P/L444P mutations were incubated without (control, CTRL) or with 6 different nominal concentrations (0.05 nM-30 µM or 10 nM -30 µM) of **PrS[5]**^{PIP} using isofagomine (IFG; 100 nM) and ambroxol (ABX; 3 µM) as control. GCase activity was determined in intact cell as described. The GCase enzyme activity data in CTRL fibroblasts were expressed as mean ± standard deviation (n=8), while in treated fibroblasts the data were expressed as mean ± standard deviation (n=5). Different colors represent independent experiments.

a)

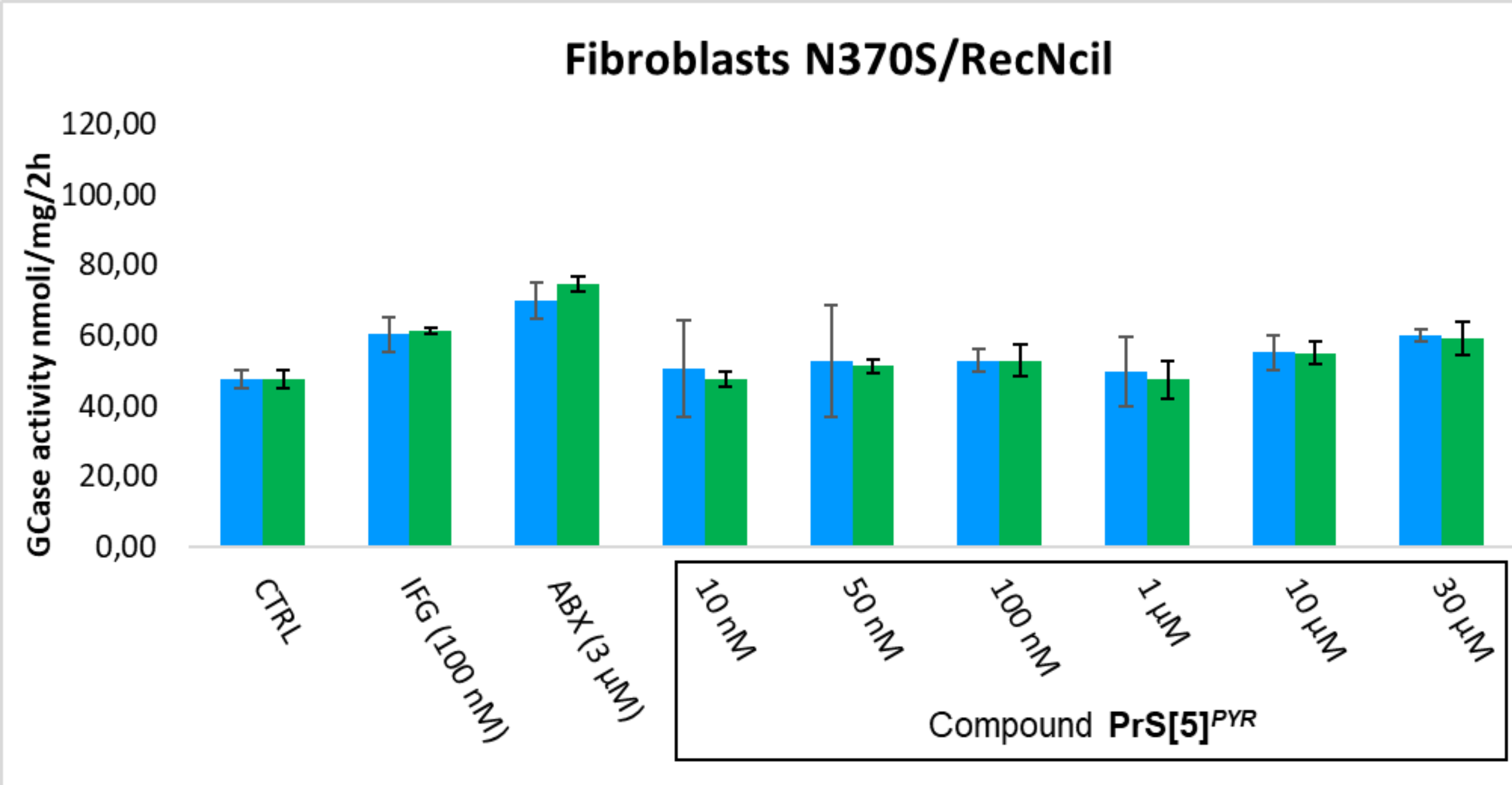

b)

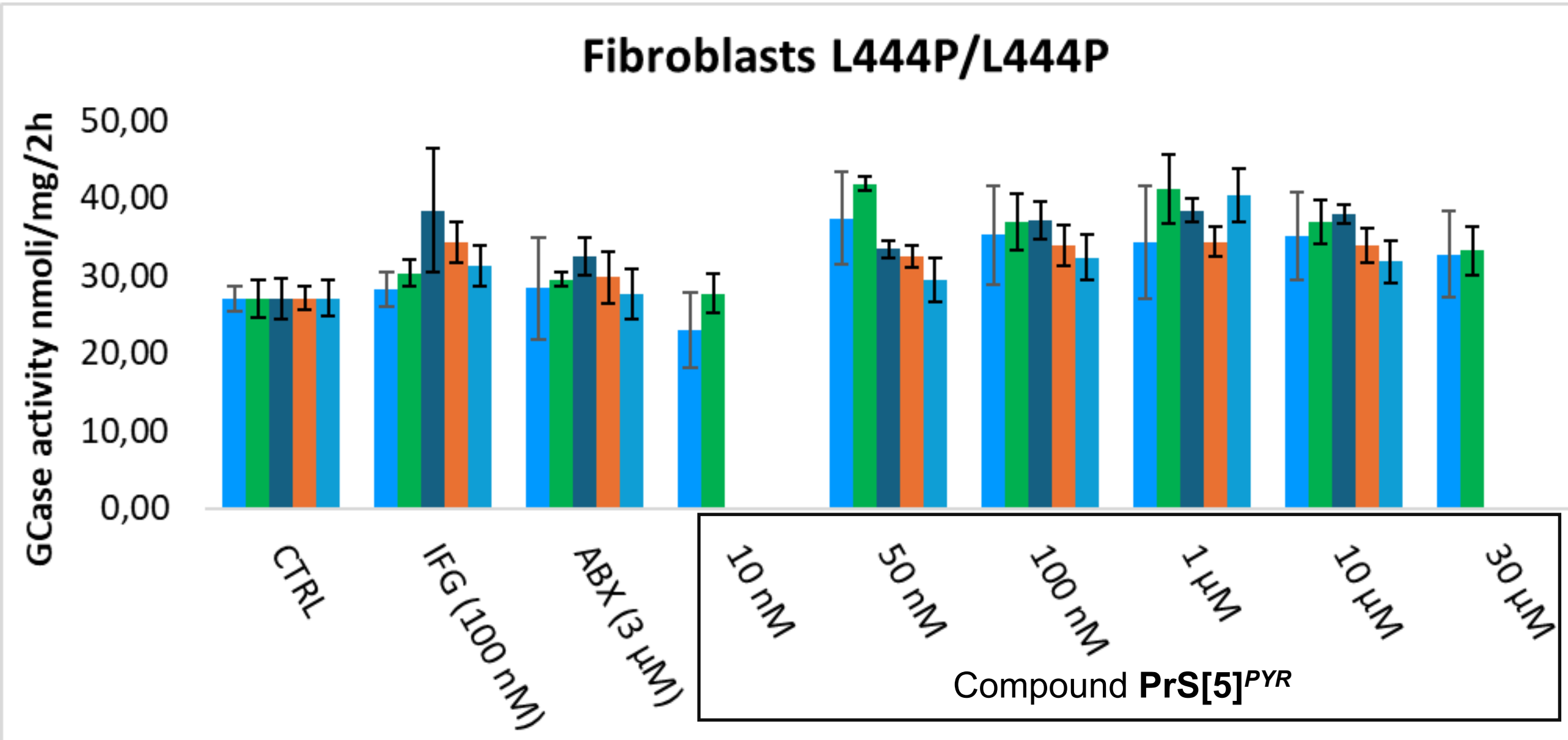

Figure S26: Fibroblasts derived from GD patients bearing a) N370S/RecNcil and b) L444P/L444P mutations were incubated without (control, CTRL) or with 6 different nominal concentrations (10 nM -30 µM) of **PrS[5]*PYR*** using isofagomine (IFG; 100 nM) and ambroxol (ABX; 3 µM) as control. GCase activity was determined in intact cell as described. The GCase enzyme activity data in CTRL fibroblasts were expressed as mean ± standard deviation (n=8), while in treated fibroblasts the data were expressed as mean ± standard deviation (n=5). Different colors represent independent experiments.

In Silico Calculation

Protein Preparation. The X-ray crystal structure of the GCase dimer (Protein Data Bank (PDB) entry 6t13)[12] was used. Hydrogen atoms were added, and ligands and crystallization solvents were removed using Hermes (version 2025.3.3).

Docking calculations were performed with the Autodock-VINA,[13] program implemented in YASARA (software version 25.9.17),[14] using AMBER14 force field. Docking studies were carried out by centering a cubic grid box (60 x 60 x 60 Å) on the indole nitrogen atom of Trp348. Fifty docking runs were performed, and the ligand was treated as fully flexible. A total of 38 and 40 poses were generated for all-p*R* and all-p*S* **PrS[5]*PIP***, respectively.

Molecular dynamics simulations were performed were performed on Intel Xeon GOLD 5118 processors. Each complex was placed in a cubic box 100 x 100 x 100 Å with periodic boundaries in water (pH: 5.2). The systems were simulated in NVT ensemble at 298 K for 10 ns. The systems were then energy minimized using first a steepest descent minimization and then by a simulated annealing minimization until convergence (<0.01 kcal/mol Å).

Non-covalent interaction (NCI) analyses were performed using the Multiwfn program,[15] based on the minimum-energy structures obtained from molecular dynamics simulations.

The resulting structure was then visualized using Chemcraft (version 1.8), generating the NCI plots from the promolecular electron density.[16]

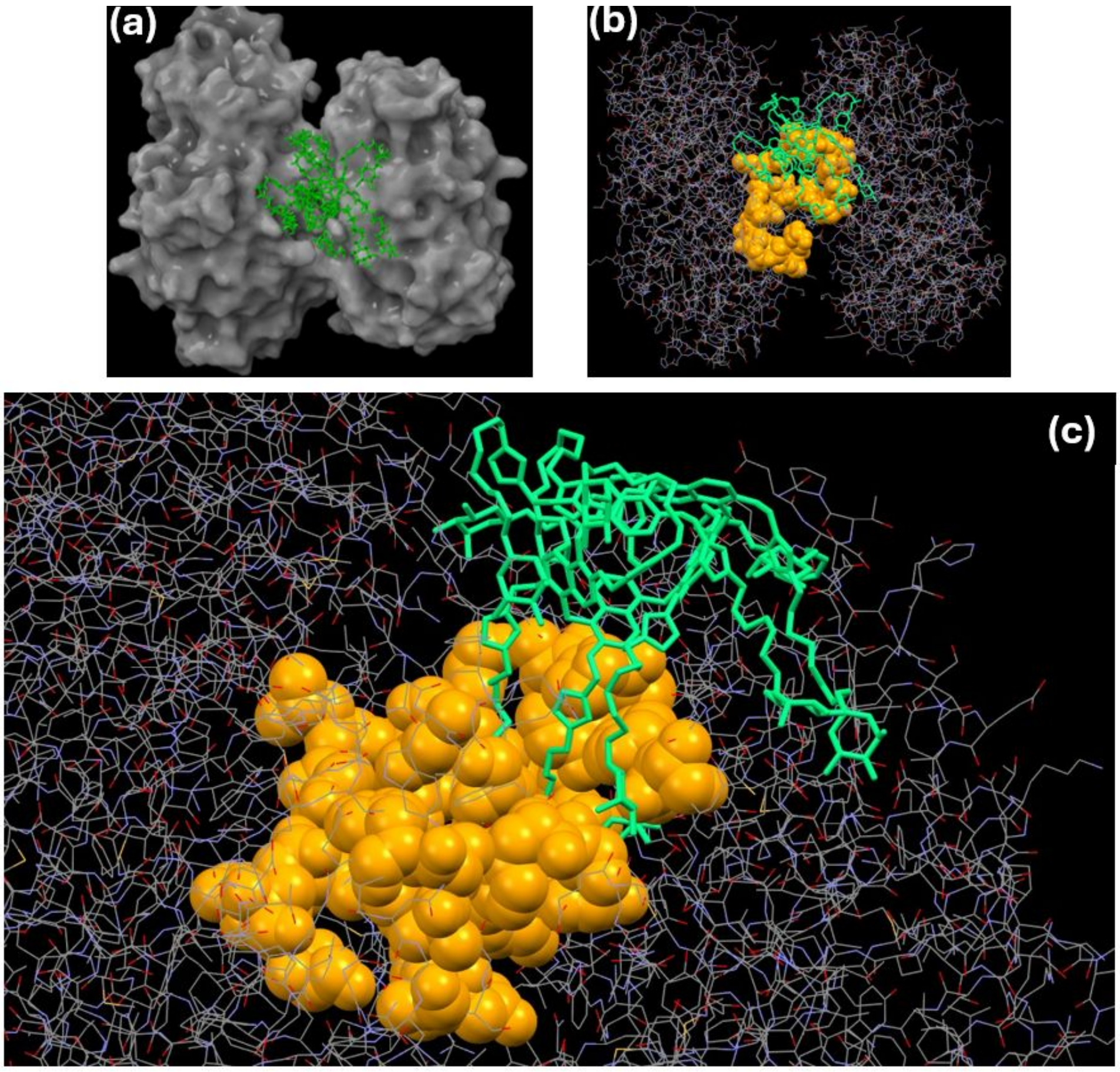

Figure S27: a-b) Global view of all-p*R* **PrS[5]*PIP*@(GC)$_2$** of the best score-ranked structure obtained from docking simulation. c) Lateral view of all-p*R* **PrS[5]*PIP*@(GC)$_2$** of best score-ranked structure obtained by docking simulation (one enzyme monomer was omitted for clarity)

Table S1. List of the best binding energy, dissociation constant, and contact surface values for all-p*R* **PrS[5]^{PIP}@(GC)$_2$** obtained from docking simulation:

**Clu**	Bind.energy spread [kcal/mol]	Dissoc. constant [pM]	Con.Surf[A^2]
**001**	07.3330+-0.0000	00000004215720.5419	2058.19
**002**	07.1650+-0.0490	00000005597791.2132	2034.74
**003**	06.9370+-0.1489	00000008225117.6351	1882.06
**004**	07.0390+-0.0820	00000006924296.6164	2009.12
**005**	06.9900+-0.0000	00000007521300.5371	1988.13
**006**	06.8360+-0.0410	00000009753839.8406	1959.50
**007**	06.8440+-0.0000	00000009623025.8634	1950.22
**008**	06.8330+-0.0000	00000009803349.2912	1818.18
**009**	06.6865+-0.1245	00000012553360.8454	1929.42
**010**	06.8070+-0.0000	00000010243128.7594	1926.54
**011**	06.7095+-0.0645	00000012075377.4329	1928.55
**012**	06.6625+-0.0565	00000013072302.4922	2056.98
**013**	06.6115+-0.0645	00000014247388.6803	2029.94
**014**	06.6510+-0.0000	00000013328508.8109	1974.02
**015**	06.6380+-0.0000	00000013624188.4144	1779.23

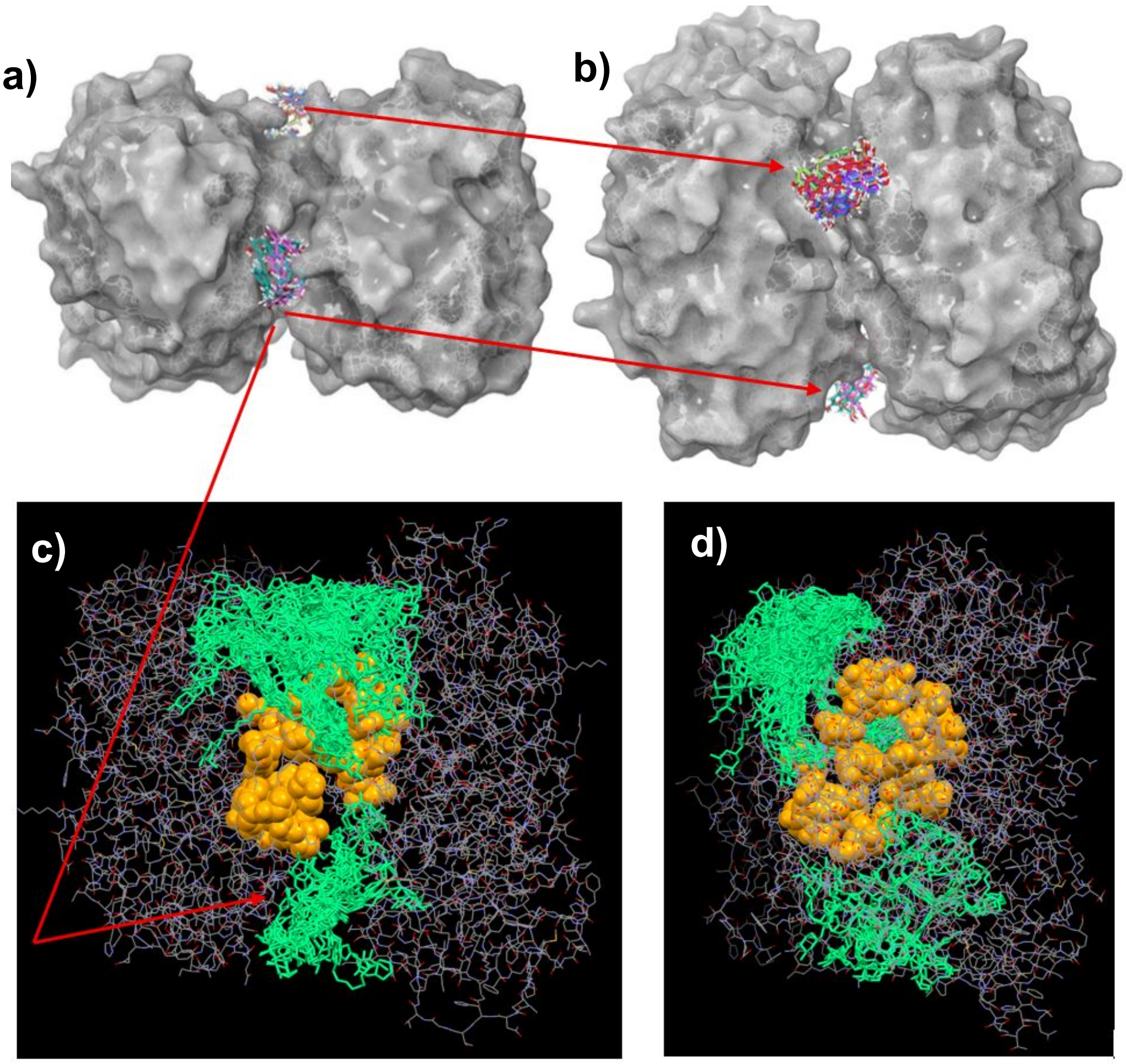

Figure S28: Overlay of the 15 best-ranked poses of all-p*R* **PrS[5]^{PIP}@(GC)$_2$ a–b)** frontal and lateral views highlighting the prismarene scaffold interacting with the enzyme surface;

c–d) views highlighting the interaction of the multivalent system within the enzyme pocket (shown in orange).

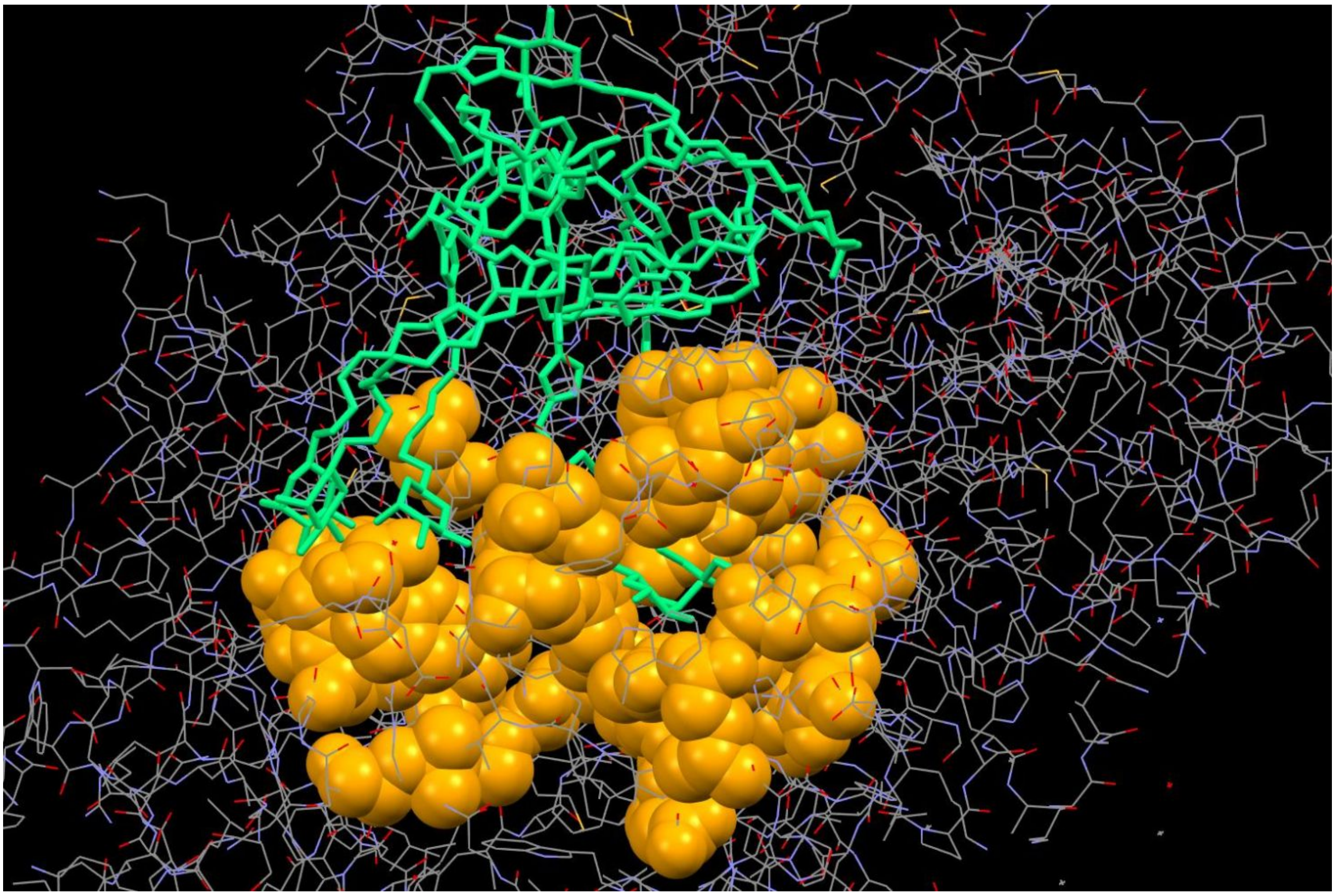

Figure S29. Lateral view of all-p*S* **PrS[5]^{PIP}@(GC)$_2$** of the best score-ranked structure obtained by docking simulation (one enzyme monomer was omitted for clarity).

Table S2. List of the best binding energy, dissociation constant, and contact surface values for all-p*S* **PrS[5]^{PIP}@(GC)$_2$** obtained by docking simulation:

**Clu**	Bind.energy spread [kcal/mol]	Dissoc. constant [pM]	Con.Surf[A^2]
**001**	06.4150+-0.2816	00000019850473.2427	1879.10
**002**	06.5095+-0.0335	00000016923953.1403	2338.05
**003**	06.3607+-0.1062	00000021756924.9547	2348.28
**004**	06.4490+-0.0000	00000018743415.9307	1742.66
**005**	06.4140+-0.0000	00000019884002.8705	1800.79
**006**	06.4070+-0.0000	00000020120317.6129	2008.41
**007**	06.3530+-0.0000	00000022040276.9270	1930.06
**008**	06.3050+-0.0000	00000023900200.7395	1757.63
**009**	06.2620+-0.0000	00000025699258.3497	2325.85
**010**	06.2600+-0.0000	00000025786149.4075	1842.26
**011**	06.2410+-0.0000	00000026626472.5308	1824.80
**012**	06.2240+-0.0000	00000027401533.6629	1609.46
**013**	06.2150+-0.0000	00000027820939.3993	2050.08
**014**	06.1375+-0.0725	00000031708798.0390	1753.91
**015**	06.1750+-0.0200	00000029764046.5651	2205.31

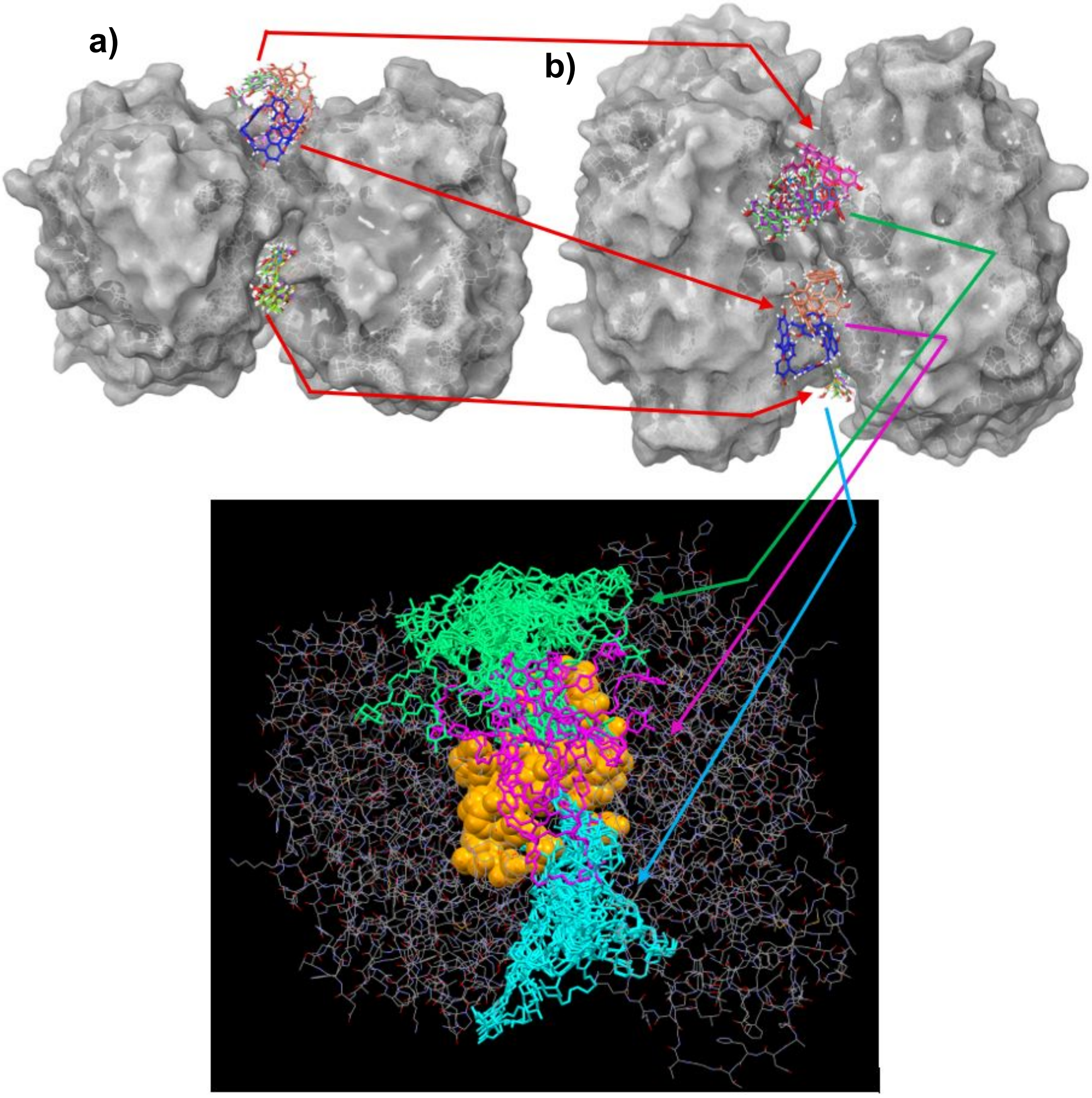

Figure S30: Overlapping of the 15 best ranked poses of all-p*S* **PrS[5]*^{PIP}*@(GC)$_2$** a–b) frontal and lateral views highlighting the prismarene scaffold interacting with the enzyme surface; c) views highlighting the interaction of the multivalent system within the enzyme pocket (shown in orange).

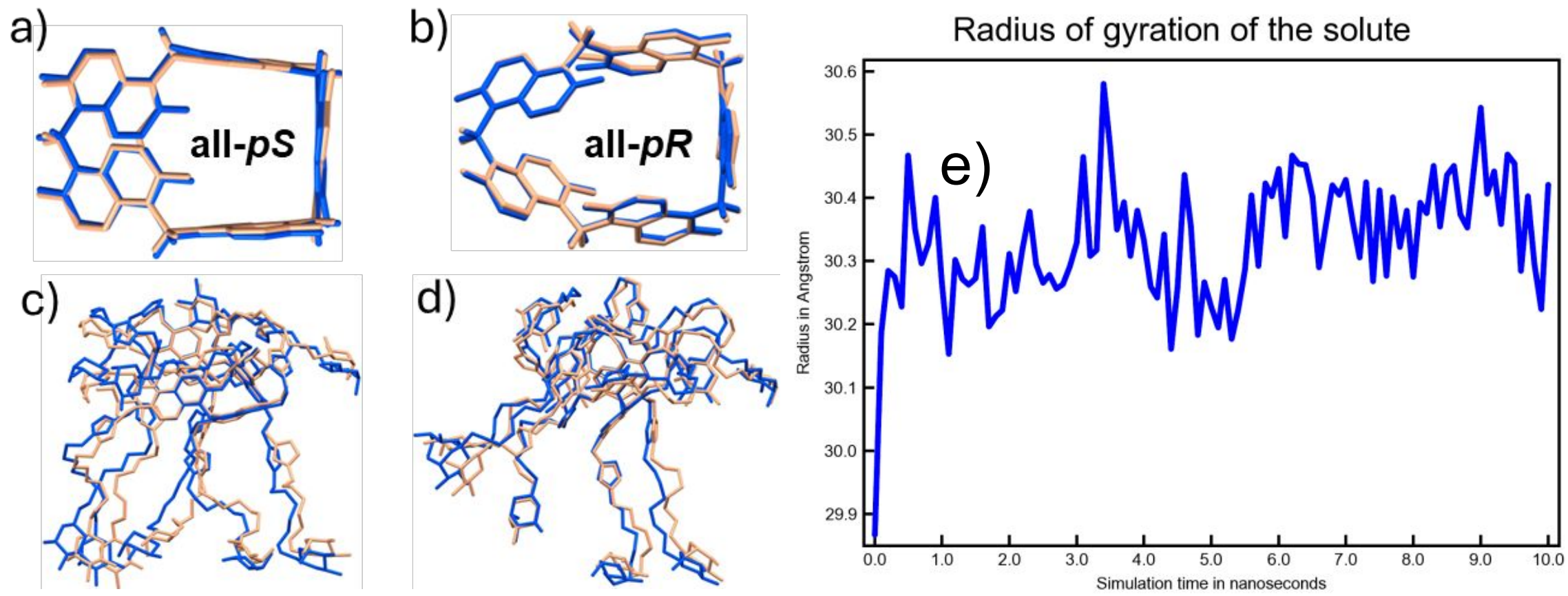

Figure S31. a-b) Overlay of only **PrS[5]*PIP*** macrocyclic scaffolds before (blue) and after (yellow) molecular dynamics simulation; c-d) Overlay of **PrS[5]*PIP*** structures before (blue) and after (yellow) molecular dynamics simulation; e) Monitoring of the radius of gyration of the all-p*R* **PrS[5]*PIP*** throughout the MD simulation.

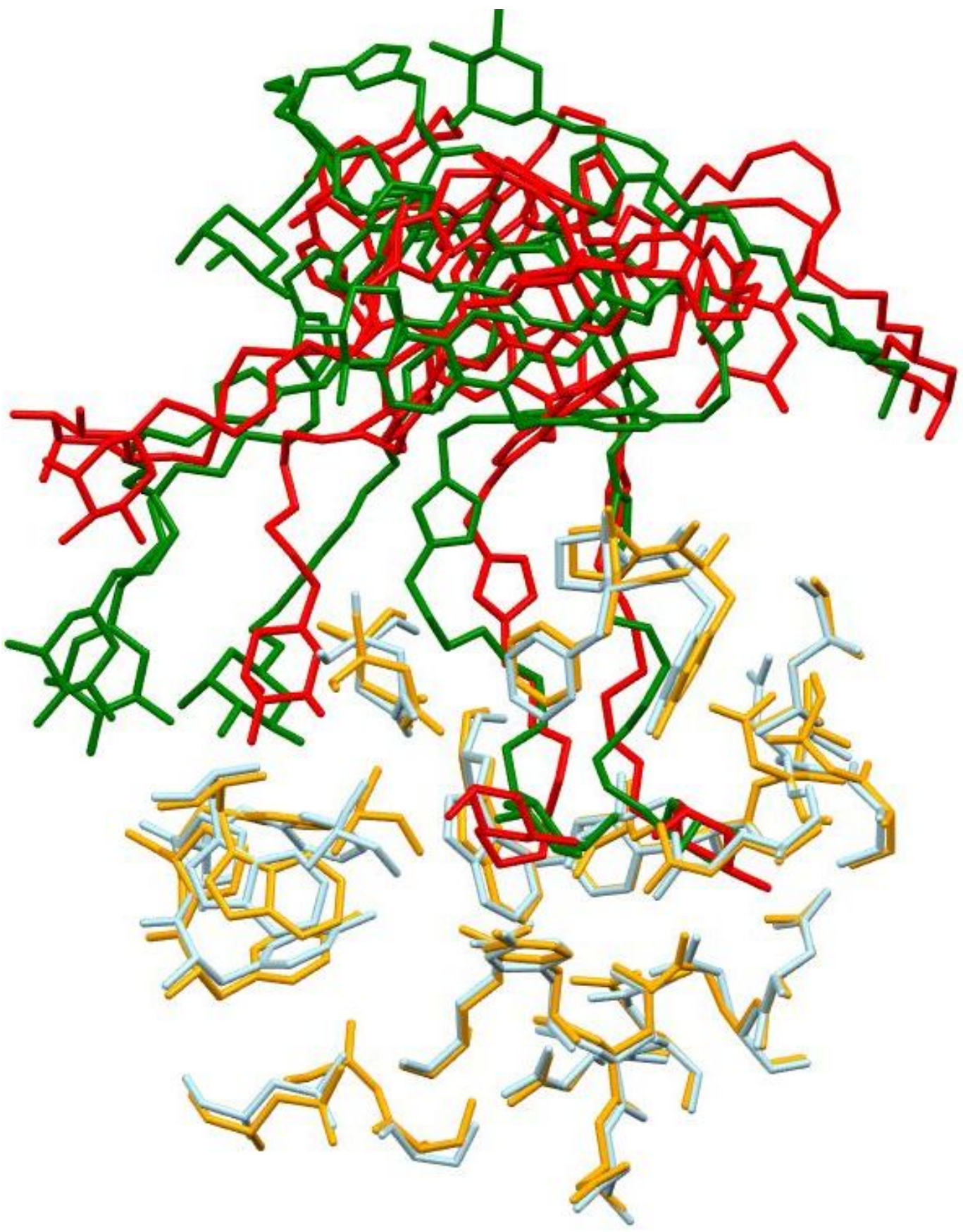

Figure S32. Lateral view of overlapped all-p*R* **PrS[5]*PIP*@(GC)₂** and **all-p*S* PrS[5]*PIP*@(GC)₂** structures respectively in red/blue and green/yellow (ligand/active site).

The number of hydrogen-bonding interactions was monitored throughout the entire simulation, including intra-ligand hydrogen bonds (blue), protein–ligand hydrogen bonds (green), ligand–water hydrogen bonds (red), and the total number of hydrogen bonds (sum of all contributions).

The number of protein–ligand hydrogen bonds varied from 10 to 26 during the first simulation, whereas in the second case a less pronounced variation was observed, together with a lower average number of hydrogen bonds (average 14 H-Bond).

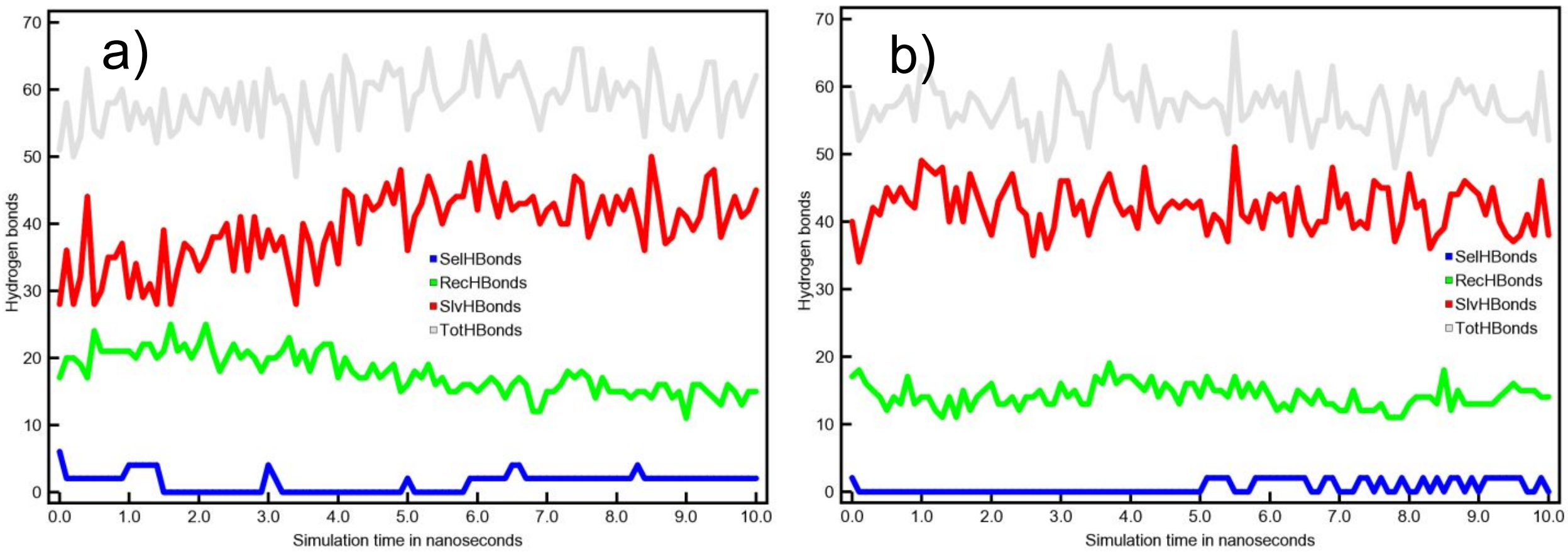

Figure S33. Monitoring of H-bond throughout the MD simulation on a) all-p*R* **PrS[5]*PIP*** and b) all-p*S* **PrS[5]*PIP***.

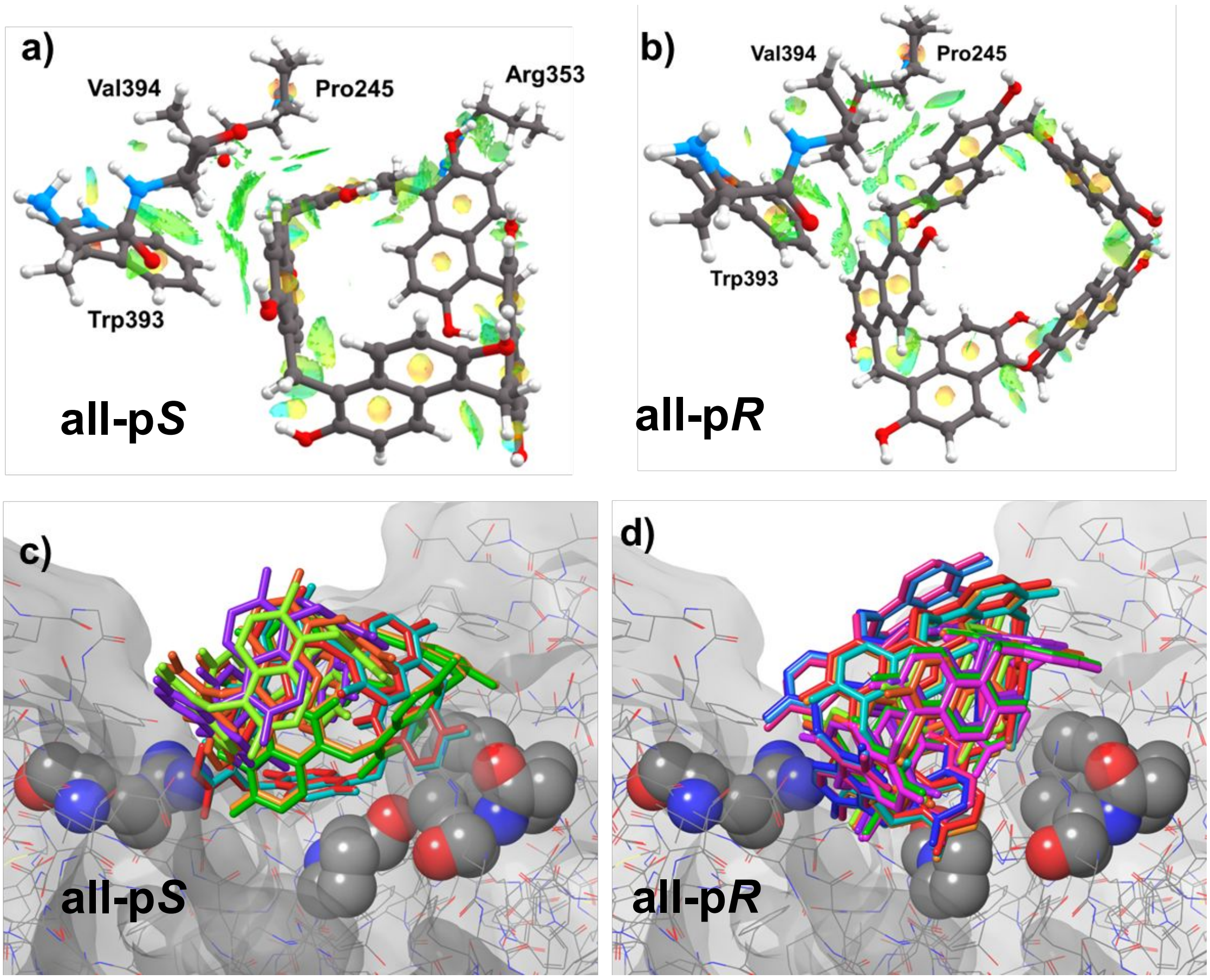

Figure S34. a-b) Gradient RDG isosurfaces (0.30) from NCI analysis based on the promolecular density, showing the interactions between the prismarene scaffold and enzyme residues responsible for stabilization above the active site (the pendants of the **PrS[5]*PIP*** is removed for clarity). c-d) Overlay of **PrS[5]*PIP*** macrocyclic scaffolds obtained from molecular docking. The CPK representation highlights the protein residues interacting with the macrocyclic framework.

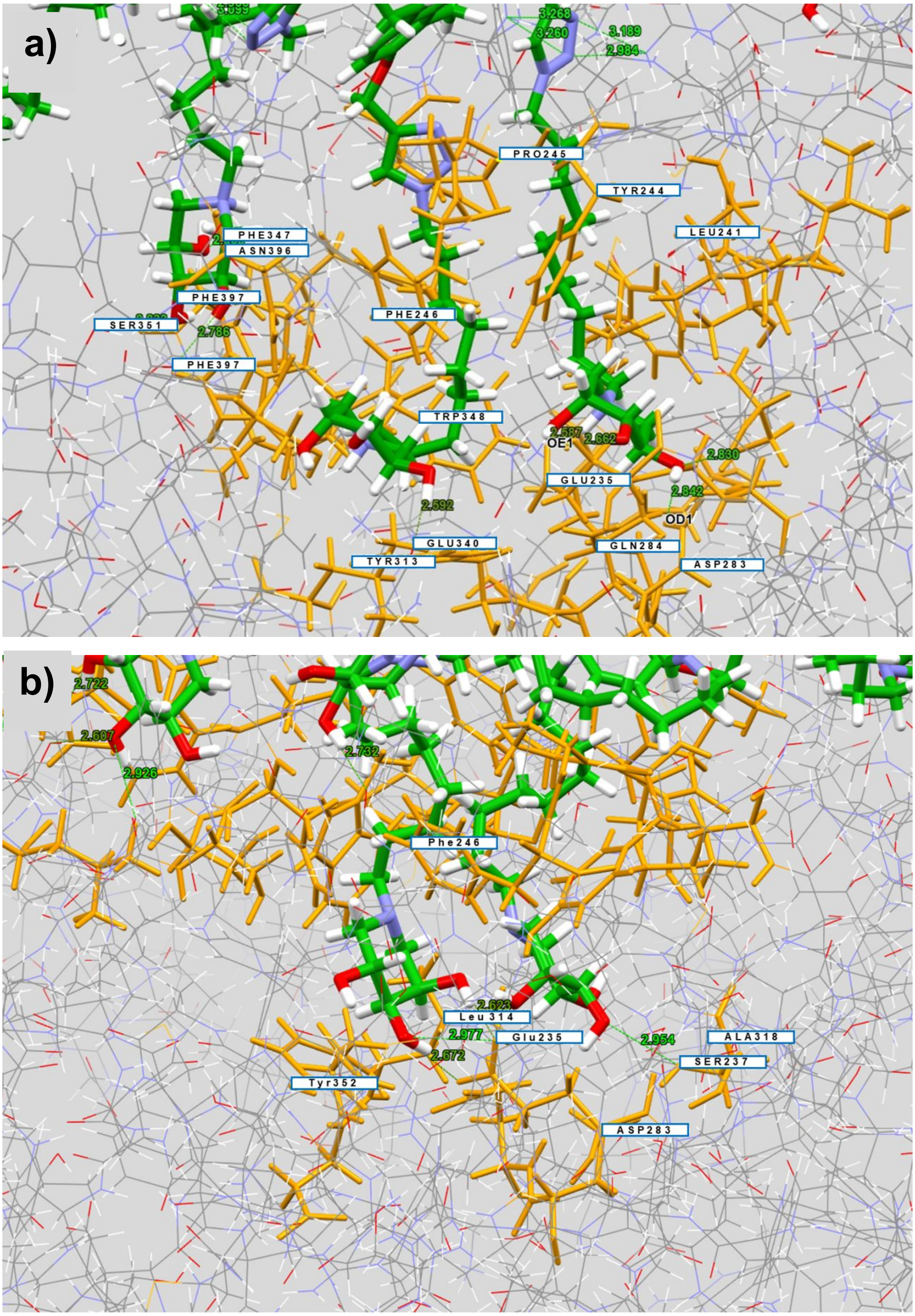

Figure S35. Detailed views of the active site of the GCase dimer (orange) interacting with a) all-p*R* **PrS[5]**^{PIP} and b) all-p*S* **PrS[5]**^{PIP}.

References

1. Fulmer, G. R.; Miller, A. J. M.; Sherden, N. H.; Gottlieb, H. E.; Nudelman, A.; Stoltz, B.M.; Bercaw, J.E.; Goldberg, K.I. NMR Chemical Shifts of Trace Impurities: Common Laboratory Solvents, Organics, and Gases in Deuterated Solvents Relevant to the Organometallic Chemist. *Organometallics* **2010**, *29*, 2176–2179.

2. Del Regno, R.; Della Sala, P.; Picariello, D.; Talotta, C.; Spinella, A.; Neri, P.; Gaeta, C. *per*-Hydroxylated Prism[n]arenes: Supramolecularly Assisted Demethylation of Methoxy-Prism[5]arene. *Org. Lett.* **2021**, *23*, 8143−8146.

3. Hoogboom, J.; Swager, T. M. Increased Alignment of Electronic Polymers in Liquid Crystals via Hydrogen Bonding Extension. *J. Am. Chem. Soc.* **2006**, *128*, 15058−15059.

4. Zámbó, G. G.; Mayr, J.; Sauer, M. J.; Schlachta, T. P.; Reich, R. M.; Kühn, F. E. The First Macrocyclic Abnormally Coordinating Tetra-1,2,3-triazole-5-ylidene Iron Complex: A Promising Candidate for Olefin Epoxidation. *Dalton Trans.* **2022**, *51*, 13591−13595.

5. Vanni, C.; Clemente, F.; Paoli, P.; Morrone, A.; Matassini, C.; Goti, A.; Cardona, F. 3,4,5-Trihydroxypiperidine Based Multivalent Glucocerebrosidase (GCase) Enhancers. *ChemBioChem.* **2022**, *23*, e202200077.

6. D'Adamio, G.; Matassini, C.; Parmeggiani, C.; Catarzi, S.; Morrone, A.; Goti, A.; Paoli, P.; Cardona, F. Evidence for a multivalent effect in inhibition of sulfatases involved in lysosomal storage disorders. *RSC. Adv.* **2016**, *6*, 64847−64851.

7. (a) Parmeggiani, C.; Catarzi, S.; Matassini, C.; D'Adamio, G.; Morrone, A.; Goti, A.; Paoli, P.; Cardona, F. Human Acid β-Glucosidase Inhibition by Carbohydrate Derived Iminosugars: Towards New Pharmacological Chaperones for Gaucher Disease. *ChemBioChem* **2015**, *16*, 2054–2064. (b) Clemente, F.; Matassini, C.; Goti, A.; Morrone, A.; Paoli, P.; Cardona, F. Stereoselective Synthesis of C-2 Alkylated Trihydroxypiperidines: Novel Pharmacological Chaperones for Gaucher Disease. *ACS Med. Chem. Lett.* **2019**, *10*, 621–626. (c) Clemente, F.; Matassini, C.; Faggi, C.; Giachetti, S.; Cresti, C.; Morrone, A.; Paoli, P.; Goti, A.; Martínez-Bailén, M.; Cardona, F. Glucocerebrosidase (GCase) Activity Modulation by 2-Alkyl Trihydroxypiperidines: Inhibition and Pharmacological Chaperoning. *Bioorg. Chem.* **2020**, *98*, 103740. (d) Urban, D. J.; Zheng, W.; Goker-Alpan, O.; Jadhav, A.; LaMarca, M. E.; Inglese, J.; Sidransky, E.; Austin, C. P. Optimization and Validation of Two Miniaturized Glucocerebrosidase Enzyme Assays for High Throughput Screening. *Comb. Chem. High Throughput Screen.* **2008**, *11*, 817–824.

8. Tonin, R.; Catarzi, S.; Caciotti, A.; Procopio, E.; Marini, C.; Guerrini, R.; Morrone, A. Progressive Myoclonus Epilepsy in Gaucher Disease due to a New Gly-Gly Mutation Causing Loss of an Exonic Splicing Enhancer. *J. Neurol.* **2019**, *266*, 92–101.

9. Urban, D. J.; Zheng, W.; Goker-Alpan, O.; Jadhav, A.; LaMarca, M. E.; Inglese, J.; Sidransky, E.; Austin, C. P. Optimization and Validation of Two Miniaturized

Glucocerebrosidase Enzyme Assays for High Throughput Screening. *Comb. Chem. High Throughput Screen.* **2008**, *11*, 817–824.

10. Li, H.-Y.; Chen, W.-A.; Lin, H.-Y.; Tsai, C.-W.; Chiu, Y.-T.; Yun, W.-Y.; Lee, N.-C.; Chien, Y.-H.; Hwu, W.-L.; Cheng, W.-C. A Practical Synthesis of Nitrone-Derived C5a-Functionalized Isofagomines as Protein Stabilizers To Treat Gaucher Disease. *Commun. Chem.* **2024**, *7*, 91.

11. Sawkar, A. R.; Cheng, W.-C.; Beutler, E.; Wong, C.-H.; Balch, W. E.; Kelly, J. W. Chemical Chaperones Increase the Cellular Activity of N370S β-Glucosidase: A Therapeutic Strategy for Gaucher Disease. *Proc. Natl. Acad. Sci. U.S.A.* **2002**, *99*, 15428–15433.

12. Benz, J.; Rufer, A. C.; Huber, S.; Ehler, A.; Hug, M.; Topp, A.; Guba, W.; Hofmann, E. C.; Jagasia, R.; Rodríguez Sarmiento, R. M. Novel β-Glucocerebrosidase Activators That Bind to a New Pocket at a Dimer Interface and Induce Dimerization. *Angew. Chem. Int. Ed*. **2021**, *60*, 5436−5442.

13. Trott, O.; Olson, A. J. AutoDock Vina: Improving the Speed and Accuracy of Docking with a New Scoring Function, Efficient Optimization, and Multithreading. *J Comput Chem* **2010**, *31*, 455.

14. Krieger, E.; Vriend, G. YASARA View-Molecular Graphics for All Devices—from Smartphones to Workstations. *Bioinformatics* **2014**, *30* (20), 2981–2982.

15. Lu, T.; Chen, F. Multiwfn: A Multifunctional Wavefunction Analyzer. *J Comput Chem* **2012**, *33* (5), 580–592.

16. Johnson, E. R.; Keinan, S.; Mori-Sánchez, P.; Contreras-García, J.; Cohen, A. J.; Yang, W. Revealing Noncovalent Interactions. *J. Am. Chem. Soc.* **2010**, *132* (18), 6498–6506.
